# Cross‐sectional relation of long‐term glucocorticoids in hair with anthropometric measurements and their possible determinants: A systematic review and meta‐analysis

**DOI:** 10.1111/obr.13376

**Published:** 2021-11-22

**Authors:** Eline van der Valk, Ozair Abawi, Mostafa Mohseni, Amir Abdelmoumen, Vincent Wester, Bibian van der Voorn, Anand Iyer, Erica van den Akker, Sanne Hoeks, Sjoerd van den Berg, Yolanda de Rijke, Tobias Stalder, Elisabeth van Rossum

**Affiliations:** ^1^ Department of Internal Medicine, Division of Endocrinology, Erasmus MC University Medical Center Rotterdam Rotterdam The Netherlands; ^2^ Obesity Center CGG, Erasmus MC University Medical Center Rotterdam Rotterdam The Netherlands; ^3^ Department of Pediatrics, division of Endocrinology, Erasmus MC‐Sophia Children's Hospital University Medical Center Rotterdam Rotterdam The Netherlands; ^4^ Department of Anesthesiology Erasmus University Medical Center Rotterdam Netherlands; ^5^ Department of Clinical Chemistry, Erasmus MC University Medical Center Rotterdam Rotterdam The Netherlands; ^6^ Department of Clinical Psychology University of Siegen Siegen Germany

**Keywords:** adiposity, hair glucocorticoids, meta‐analysis, systematic review

## Abstract

**Background:**

Long‐term glucocorticoids (HairGC) measured in scalp hair have been associated with body mass index (BMI), waist circumference (WC), and waist‐hip‐ratio (WHR) in several cross‐sectional studies. We aimed to investigate the magnitude, strength, and clinical relevance of these relations across all ages.

**Methods:**

We performed a systematic review and meta‐analysis (PROSPERO registration CRD42020205187) searching for articles relating HairGC to measures of obesity. Main outcomes were bivariate correlation coefficients and unadjusted simple linear regression coefficients relating hair cortisol (HairF) and hair cortisone (HairE) to BMI, WC, and WHR.

**Results:**

We included *k* = 146 cohorts (*n* = 34,342 individuals). HairGC were positively related to all anthropometric measurements. The strongest correlation and largest effect size were seen for HairE‐WC: pooled correlation 0.18 (95%CI 0.11–0.24; *k* = 7; *n* = 3,158; *I* ^2^ = 45.7%) and pooled regression coefficient 11.0 cm increase in WC per point increase in 10‐log‐transformed HairE (pg/mg) on liquid‐chromatography‐(tandem) mass spectrometry (LC–MS) (95%CI 10.1–11.9 cm; *k* = 6; *n* = 3,102). Pooled correlation for HairF‐BMI was 0.10 (95%CI 0.08–0.13; *k* = 122; *n* = 26,527; *I* ^2^ = 51.2%) and pooled regression coefficient 0.049 kg/m^2^ per point increase in 10‐log‐transformed HairF (pg/mg) on LC–MS (95%CI 0.045–0.054 kg/m^2^; *k* = 26; *n* = 11,635).

**Discussion:**

There is a consistent positive association between HairGC and BMI, WC, and WHR, most prominently and clinically relevant for HairE‐WC. These findings overall suggest an altered setpoint of the hypothalamic–pituitary–adrenal axis with increasing central adiposity.

## 1 BACKGROUND

The prevalence of obesity, defined in adults as a body mass index (BMI; weight in kg divided by height in meters squared) ≥ 30 kg/m^2^, has increased dramatically worldwide over the past decades.^1^ An imbalance between energy intake and expenditure is regarded as the major cause of obesity. Numerous distinct characteristics and conditions can contribute to obesity within an individual.^2^ One important contributing factor may be chronic exposure to the stress hormone cortisol, the major end‐product of the hypothalamic–pituitary–adrenal (HPA) axis. In healthy individuals, cortisol secretion and metabolism are closely linked and tightly regulated. Cortisol is converted by 11‐beta‐hydroxysteroid dehydrogenase type 2 (11β‐HSD‐2) to the biologically inactive cortisone in end‐organ tissues, but can be converted back to cortisol by 11‐beta‐hydroxysteroid dehydrogenase type 1 (11β‐HSD‐1) on tissue‐level.^3^ Exposure to very high levels of endogenous or exogenous glucocorticoids (GC), such as in Cushing's syndrome, leads to a phenotype characterized by abdominal obesity and other features of the metabolic syndrome.^4^, ^5^ It is hypothesized that even a chronic mild increase of GC, that is, in the high‐physiological range, can contribute to overweight and obesity in the general population.^2^ Despite many efforts over the last decades to explore this relation in different matrices such as blood, saliva and urine, conflicting results were found.^6^ This may be due to cortisol's circadian rhythm, its pulsatile secretion, and the daily variation following changing circumstances such as acute stress. Hence, measurements that reflect a shorter term (minutes or hours for serum and saliva, days for urine) seem less suitable to investigate this association in the general population.^7^

In the past decennium, a relatively novel technique has allowed researchers to study long‐term levels of GC by measuring cortisol and cortisone levels in scalp hair (HairF and HairE, respectively). Every centimeter of scalp hair is believed to represent the cumulative GC exposure of one month.^8^ HairGC measurements are now considered an easily applicable, noninvasive and reproducible method for assessing long‐term GC exposure.^8^ A systematic review and meta‐analysis by Stalder et al. that was conducted in September 2015 (when the number of studies that used HairGC started to increase rapidly) identified several possible influencers of HairF levels. The authors concluded that variation in HairF levels on study level could be related, among other factors, to differences in mean BMI of the study populations.^9^ Gray et al. and Ling et al. also reported that BMI and BMI standard deviation score (SDS), that is, BMI *z*‐scores adjusted for age and sex that are most often used in pediatric studies,^10^ were important determinants of HairF levels in children.^11^, ^12^ However, in the last years, many new large‐scale studies in various age categories have been published that have investigated the relation between HairGC and anthropometric features. Some of these studies showed a positive relation,^13^, ^14^ while other studies showed no relation between HairGC and anthropometric measurements.^15^, ^16^ It is unclear whether these conflicting results can be explained by differing population characteristics such as mean age, sex, and prevalence of obesity, use of corticosteroids, handling of outliers, or the various laboratory methods that were used.

Moreover, other anthropometric measurements than BMI are considered equally or even more relevant to cardiometabolic health, such as waist circumference (WC) and waist‐hip‐ratio (WHR), which both are markers of central adiposity.^17^ These deserve specific attention as GC are known to particularly induce abdominal obesity.^18^ Likewise, there are suggestions that hair cortisone might correlate stronger to obesity than hair cortisol itself.^19^ However, a meta‐analysis that summarizes all evidence considering different anthropometric parameters in association with both HairF and HairE as well as relevant moderators of these relationships is missing.

Therefore, the aim of the current systematic review and meta‐analysis was to investigate the cross‐sectional relations between HairGC levels (HairF and HairE) and anthropometric measurements (BMI, BMI SDS, WC, and WHR) and to explore the possible influence of relevant characteristics of the population and laboratory methods.

## 2 METHODS

We performed this systematic review and meta‐analysis in concordance with the Preferred Reporting Items for Systematic Reviews and Meta‐Analyses (PRISMA) statement and Meta‐analysis of Observational Studies in Epidemiology (MOOSE) checklist.^20^, ^21^ This systematic review was registered at the PROSPERO database (Registration number CRD42020205187, December 7, 2020).^22^

### 2.1 Search strategy and selection criteria

A university health sciences librarian designed a comprehensive search to identify studies and conference abstracts concerning hair cortisol and/or hair cortisone and measurements of obesity. To avoid missing potentially relevant papers we designed a broad search strategy combining the elements “hair,” “cortisol/cortisone,” and “BMI/WC/WHR/anthropometrics”, including their synonyms without any restrictions other than “studies in humans”. The search was conducted in the following databases from inception up to November 16, 2020: Medline (Ovid), Embase, Cochrane, Web of Science, Scopus, Cinahl, PsycInfo, and Google Scholar. The complete search strategy is provided in the supporting information Appendix S1. Search results were exported to reference management software (EndNote version X9, Clarivate Analytics), and duplicates were removed prior to screening.

**FIGURE 1:**
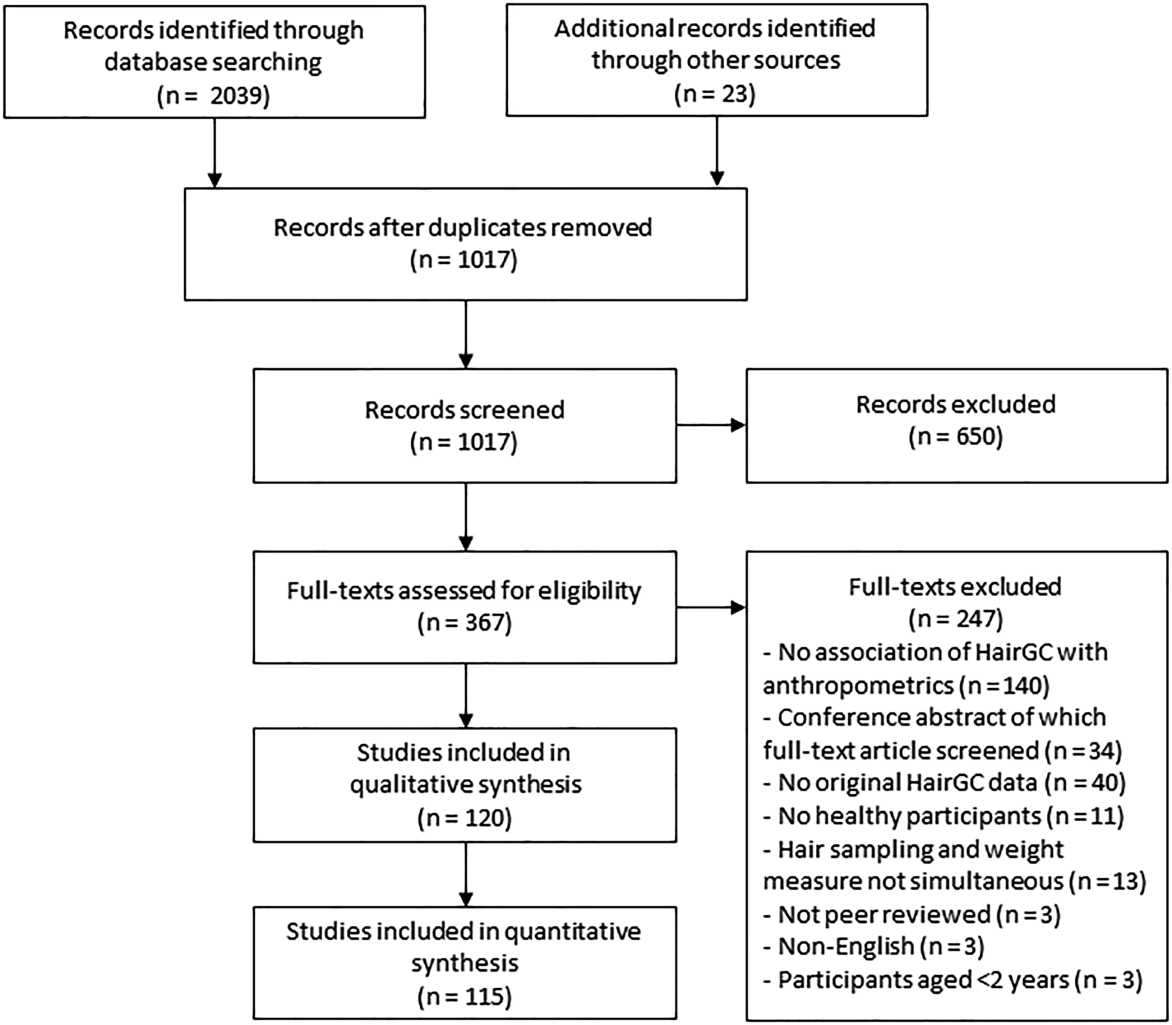
Preferred Reporting Items for Systematic Reviews and Meta‐Analyses (PRISMA) flow diagram. *HairGC*, hair glucocorticoids

All identified studies were independently screened in two stages by two physicians (EV, OA, or MM) with a background in adult (EV and MM) and pediatric (OA) endocrinology. All studies that reported original HairGC data in humans were included in the title/abstract screening stage and were subsequently assessed full text. Disagreements were solved by discussion among the first authors (EV, OA, and MM), and the senior author (EvR) until consensus was reached. Additionally, reference lists of all included studies and relevant reviews were screened systematically for potentially relevant articles.^23^ We included studies that reported cross‐sectional associations between HairGC and measurements of obesity. We excluded case reports, animal studies, review articles, non‐English or nonpeer reviewed studies, and studies in which hair sampling and weight measurements were not performed simultaneously (Figure 1). Pediatric studies that only included children younger than age 2 years were also excluded because BMI‐based definitions of obesity are not available for this age group.^10^ We contacted all corresponding authors of articles that reported both HairGC and anthropometric data but did not report an association between these two outcomes to ask if they could provide us with an association measure. Of articles that also included patients with mental or physical diseases that are known to influence the relation between GCs and obesity, we only included the separate analyses of healthy controls if available. When data of the same participants were reported in several studies, we included the study that reported a bivariate association (correlation coefficient or unstandardized simple linear regression coefficient) between HairGC and measurements of obesity. If more than one article reported a bivariate association, we included the study with the largest sample size.

### 2.2 Data extraction

Descriptive, methodological, and outcome data were extracted from all included studies by two researchers independently (EV, OA, or MM) using a predesigned standardized data extraction sheet. Discrepancies were resolved by discussion among the first authors (EV, OA, and MM) and the senior author (EvR). The following descriptive data were extracted: study population characteristics (sample size and cohort characteristics: age, sex, prevalence of obesity, mean levels of HairF and HairE in pg/mg) and laboratory methods: liquid chromatography‐(tandem) mass spectrometry based measurements (LC–MS or LC–MS/MS, in this review further collectively abbreviated as LC–MS), enzyme‐linked immunosorbent assays (ELISA), or chemiluminescent immunoassays (CLIA). The reported outcomes of interest were any cross‐sectional associations between HairGC (HairF, HairE) and measurements of obesity, that is*,* BMI, BMI SDS, WC, and WHR. In studies presenting multiple data points of the same participants (e.g., before and after an intervention), only baseline associations were extracted. When insufficient data were reported for meta‐analysis, corresponding authors were contacted twice in a 2‐week time frame. In case of nonresponse, data were extracted from previous meta‐analyses where possible.^9^, ^12^

### 2.3 Risk of bias assessment

Risk of bias was assessed by two researchers independently (EV, OA, or MM) using the Quality In Prognostic Studies (QUIPS) tool.^24^ In short, the QUIPS tool aids in the assessment of potential bias sources from the following study domains: study participation, study attrition, prognostic factor measurement, outcome measurement, confounding measurement, and statistical analysis. The subdomains on which risk of bias was assessed were the following: population selection criteria (QUIPS 1; study participation), the used laboratory methods (QUIPS 3; prognostic factor measurement), whether or not anthropometric measurements were objectively measured (QUIPS 4; outcome measurement), whether or not corticosteroid use was taken into account and whether any consideration was given to handling outliers in HairGC values (QUIPS 5; study confounding), and reporting of relevant statistics (QUIPS 6; statistical analysis and reporting). All subdomains were scored as ‘low’, ‘moderate’, or ‘high’ risk of bias on individual cohort level. We omitted the study attrition domain of the QUIPS tool (QUIPS 2) since it was not applicable to our cross‐sectional research question. Discrepancies between the researchers were solved by discussion among the first authors (EV, OA, and MM) and the senior author (EvR).

### 2.4 Qualitative synthesis

For the qualitative synthesis, we summarized all authors' conclusions regarding cross‐sectional associations between HairGC levels and obesity measurements, that is, correlation coefficients, regression coefficients, or comparison of HairGC levels and obesity measurements across categories.

### 2.5 Statistical analysis

All meta‐analyses were conducted in R version 3.6.3 with an *α* of 0.05.^25^ For all descriptive data, medians and (interquartile) ranges were converted to means and standard deviations prior to analyses.^26^ Furthermore, subgroup means from individual studies as well as the pooled means across all studies were pooled.^27^ When not originally reported, standard errors were calculated based on reported confidence intervals or *p*‐values and degrees of freedom using the *T*‐distribution.

### 2.6 Meta‐analysis of correlation coefficients

For all studies reporting bivariate correlations (correlation coefficients), Fisher's *r*‐to‐*z* transformation was applied to transform individual correlations stratified on all combinations of HairGC (HairF and HairE) and obesity measurements (BMI, BMI SDS, WC, and WHR). As several studies reported correlations within distinct subgroups, we calculated the pooled correlation coefficients, 95% confidence intervals (CIs) and prediction intervals (PIs) using multilevel random effects models.^28^, ^29^ One study was excluded for all meta‐analyses, as the reported correlation coefficient for BMI versus HairF of the total cohort was 0.91. We assume this is a typographic error, as the authors state that they only found a statistically significant correlation in the highest tertile of the polygenic susceptibility score (which was reported to be 0.269, making a correlation of 0.91 for the total cohort impossible).^30^ These authors did not respond to our contact attempts.

The *I* ^2^ statistic and Cochrane's *Q* test were used for the assessment of between‐study heterogeneity, with *I* ^2^ > 25% and *p*‐value for Cochrane's *Q* test <0.05 indicating heterogeneity. For all meta‐analyses with data from at least 10 cohorts, exploratory moderator analyses were performed using mixed‐effect models for categorical parameters (e.g., used laboratory method) and random effects models for continuous parameters (e.g., mean age of the study participants). Publication bias was assessed using contour‐enhanced funnel plots.

### 2.7 Meta‐analysis of unstandardized simple linear regression coefficients

For all studies reporting unstandardized simple linear regression coefficients between 10‐log transformed HairGC (HairF or HairE) in pg/mg as independent variable and untransformed obesity measurements (BMI, BMI SDS, WC, and WHR) as dependent variable, pooled regression coefficients and 95% CIs were calculated using the statistical approach described by Bini et al. and Becker & Wu.^31^, ^32^ In short, this approach allows pooling of linear regression coefficients using weighted least squares provided that the independent and dependent variables have been measured in the same manner across all studies. Therefore, we calculated pooled regression coefficients of 10‐log transformed HairGC on untransformed obesity measurements, stratified on laboratory method. Between‐study heterogeneity was assessed using the Q_w_‐statistic described by Bini et al.^31^

## 3 RESULTS

The literature search identified 1017 unique citation titles of which a total of 120 studies^5^, ^13^, ^14^, ^16^, ^19^, ^30^, ^33^, ^34^, ^35^, ^36^, ^37^, ^38^, ^39^, ^40^, ^41^, ^42^, ^43^, ^44^, ^45^, ^46^, ^47^, ^48^, ^49^, ^50^, ^51^, ^52^, ^53^, ^54^, ^55^, ^56^, ^57^, ^58^, ^59^, ^60^, ^61^, ^62^, ^63^, ^64^, ^65^, ^66^, ^67^, ^68^, ^69^, ^70^, ^71^, ^72^, ^73^, ^74^, ^75^, ^76^, ^77^, ^78^, ^79^, ^80^, ^81^, ^82^, ^83^, ^84^, ^85^, ^86^, ^87^, ^88^, ^89^, ^90^, ^91^, ^92^, ^93^, ^94^, ^95^, ^96^, ^97^, ^98^, ^99^, ^100^, ^101^, ^102^, ^103^, ^104^, ^105^, ^106^, ^107^, ^108^, ^109^, ^110^, ^111^, ^112^, ^113^, ^114^, ^115^, ^116^, ^117^, ^118^, ^119^, ^120^, ^121^, ^122^, ^123^, ^124^, ^125^, ^126^, ^127^, ^128^, ^129^, ^130^, ^131^, ^132^, ^133^, ^134^, ^135^, ^136^, ^137^, ^138^, ^139^, ^140^, ^141^, ^142^, ^143^, ^144^, ^145^, ^146^ comprising 146 separate cohorts were included (Figure 1). This corresponds to a total of 34,342 included participants of which 15,698 (46%) were sampled from general population‐based studies (Table 1). The remaining 18,644 (54%) participants were sampled from studies where study inclusion was based on medical criteria (e.g., individuals with obesity), occupational characteristics (e.g., health‐care workers), or socio‐economic characteristics (e.g., children from low‐income parents). The majority of participants (24,004; 70%) were sampled from studies in adults (mean age ≥18 years). Most studies analyzed participants living in Germany (32/146 cohorts, 22%), The Netherlands (23/146 cohorts, 16%), and Canada (18/146 cohorts, 12%). For 70/146 cohorts (48%), correlation coefficients and/or regression coefficients that were not reported in original papers were obtained by contacting authors.

**TABLE 1:** Overview of included cohorts

Study	*n*	Age in years	BMI in kg/m^2^ Or BMI SDS M ± SD	% male	% obesity	HairF in pg/mg	HairE in pg/mg	HairGC analysis	Risk of bias^a^	Reported bivariate correlations	Reported regression coefficients
		M ± SD				M ± SD	M ± SD				
**Adult cohorts**											
Abdulateef et al. (2019)	65	33.1 ± 10.4	26.4 ± 5.7	9.8	28.1	17.2		ELISA	23311	**A**C	AC
Abell et al. (2016)	3,634	69.8 ± 5.8	26.7 ± 4.5	68.4	19.8	12.6 ± 46.4		LC–MS	21121	**ACD**	**A**
Aguilo et al. (2018)	53	56.7 ± 12.5	25.1 ± 3.9	30.2	9.4	14.0 ± 9.0		ELISA	23321	**A**	**A**
Berger et al. (2019)—Cohort WPHC	207	40.3 ± 16.9	31.5 ± 7.2	44.4		14.2 ± 27.8		ELISA	23331	A	
Berger et al. (2019)—Cohort YPC	122	19.4 ± 3.1	25.2 ± 6.9	43.4		7.8 ± 9.3		ELISA	23331	A	
Boesch et al. (2014)	177	20.1 ± 1.1	23.6 ± 3.1	100		358.8 ± 159.1		ELISA	23131	A	
Bossé et al. (2018)	598	64.9 ± 6.8	29.3 ± 6.5	80.6	36.9	11.9 ± 26.7		CLIA	32121	**AC**	**AC**
Brianda et al. (2020)	134		24.6 ± 4.4	7.1		82.3 ± 94.3		ELISA	23311	**A**	**A**
Castro‐Vale et al. (2020)	128	49.1 ± 15.5	27.0 ± 3.9	68		4.7 ± 3.7		LC–MS	21331	A	A
Cedillo et al. (2020)	62	29.2 ± 7.5^b^	30.0 ± 7.7^b^	0	27.0	130.7 ± 124.5^b^		ELISA	23131		A
Chan et al. (2014)	57	44.5 ± 12.5^b^ ^,^ ^c^	27.6 ± 6.8^b^ ^,^ ^c^	45.6	33.3	98.8 ± 74.8^b^ ^,^ ^c^		ELISA	33121	AC	
Chen et al. (2013)	53	40.7 ± 6.6	22.4 ± 2.9	98.11		18.9 ± 13.6		LC–MS	22331	A	
Chen et al. (2015)—female adults	75	43.3 ± 8.9	30.2 ± 5.5	0		4.6 ± 3.4		LC–MS	21121	AD	
Chen et al. (2015)—male adults	10	41.6 ± 9.2	29.4 ± 1.9	100		3.1 ± 1.5		LC–MS	21121	AD	
Davison et al. (2019)	344	25.4 ± 1.5^b^ ^c^	23.7 ± 6.3	43		4.3 ± 4.9	6.3 ± 5.8	LC–MS	11121	AE	
Dettenborn et al. (2010)—employed	28	32.6 ± 9.3	22.6 ± 3.8	42.9		7.1 ± 3.0		CLIA	22321	A	
Dettenborn et al. (2010)—unemployed	31	36.7 ± 11.0	24.6 ± 6.3	3.2		10.2 ± 7.2		CLIA	22321	**A**	
Diebig et al. (2016)	129	32.3 ± 12.1	24.4 ± 4.3	24		11.6 ± 13.2		CLIA	22331	A	A
Dowlati et al. (2010)—controls	87	65.7 ± 11.1	27.5 ± 4.9	80.5		185.3 ± 131.6		ELISA	33321	A	
Enge et al. (2020)	470	38.6 ± 8.9	24.6 ± 4.9	34	8.3	6.1 ± 7.4		LC–MS	11311	**A**	**A**
Engert et al. (2018)	332	40.7 ± 9.2	23.6 ± 3.3	40.7		1.6 ± 1.0	2.5 ± 0.7	LC–MS	11131	AE	
Etwel et al. (2014)	39	23.8 ± 6.2	23.2 ± 4.8	0		257.2 ± 101.8		ELISA	23121	A	
Feeney et al. (2020)	1876	66.4 ± 8.7		25.6	31.5	18.8 ± 48.1	12.4 ± 10.3	LC–MS	11111	**AE**	**AE**
Feller et al. (2014)	654	65.8 ± 8.4	27.5 ± 4.4	46		35.1 ± 32.8		CLIA	12111	A**CD**	
Fischer et al. (2017)	139	50.6 ± 14.6	27.5 ± 6.0	28	28			ELISA	13311	A	
Gao et al. (2014)—adult control	23	41.5 ± 12.8	22.9 ± 2.7	61	0	4.3 ± 3.9		LC–MS	22321	A	
Gao et al. (2014)‐ adult earthquake survivor	20	45.5 ± 14.2	23.4 ± 2.1	60	0	46.3 ± 48.4		LC–MS	22321	A	
Garcia‐Leon et al. (2018)	62	33.0 ± 3.7	22.8 ± 2.9	0		127.9 ± 111.5		ELISA	13313	A	
Gidlow et al. (2016)	132	41.4 ± 11.4	25.1 ± 4.8	28.9		10.8 ± 9.4		ELISA	13321	A	A
Grass et al. (2015)—study I	42	24.8 ± 5.7	21.3 ± 2.9	52.4		3.5 ± 2.3		LC–MS	11311	A	
Grass et al. (2015)—study II	52	25.0 ± 4.9	22.8 ± 3.2	57.7		3.2 ± 3.8		LC–MS	11311	A	
Henley et al. (2014)	109			29.8	16.2	592.2 ± 304.8^b^		ELISA	13123	A	A
Hollenbach et al. (2018)	59	36 ± 6	32.2 ± 9.8	3.4		27.8 ± 30.8		ELISA	13321	**A**	
Hunter et al. (2020)	140	22.8 ± 6.0	27.2 ± 6.6	0	29	11.2 ± 23.5		LC–MS	21321	A	A
Jackson et al. (2017)	2,527	67.9 ± 7.3	28.2 ± 5.2	41	30.5	30.5 ± 76.7		LC–MS	11131		
Janssens et al. (2017)	111	43.4 ± 10.4	24.4 ± 3.8	60	10.8	14.9 ± 9.4^c^		LC–MS	21111	AD	AD
Kozik et al. (2015)	66	71.9 ± 5.8	25.0 ± 4.0	33.3		25.8 ± 17.2		ELISA	13321	AC	
Kuehl et al. (2015)	41	41.2	23.3 ± 3.6^b^	36.6	14.1	4.3 ± 4.2^b^	19.8 ± 21.5^b^	CLIA	22121	ACE**F**	**A**CE**G**
Lanfear et al. (2020)	41	68.1 ± 5.3		48		10.5 ± 13.6		LC–MS	11321	D	
Larsen et al. (2016)—fathers	231	40.3 ± 5.4	26.2 ± 3.7	100		177.4 ± 119.2		ELISA	23331	**A**	**A**
Larsen et al. (2016)—mothers	301	38.0 ± 4.3	26.6 ± 5.4	0		146.1 ± 102.3		ELISA	23331	**A**	**A**
Lehrer et al. (2020)	141	45.8 ± 15.2		32.6				ELISA	13122	**D**	D
Ling et al. (2020)—mothers	35	29.7 ± 5.6	32.4 ± 7.0	0	58.1	7.0 ± 8.1		ELISA	23111	**A**	A
Manenschijn et al. (2013)	283	74.8 ± 7.1^c^	27.4 ± 4.0^c^	33.9		23.2 ± 10.1^c^		ELISA	13121	AC	
Manenschijn, Koper et al. (2011)	46							ELISA	23321	**CD**	
Mazgelyte et al. (2019)	163	38.5 ± 9.3	26.6 ± 5.3^c^	100		237.8 ± 160.8^c^		LC–MS	21131	**AC**	**AC**
McLennan et al. (2016)	246	42.0 ± 11.2	26.4 ± 5.3	10.2	23.8	15.1 ± 14.6		CLIA	22311	**A**	**A**
Menning et al. (2015)—breast cancer no chemotherapy	33	52.4 ± 7.3	24.0 ± 3.8	0		23.8 ± 16.6		ELISA	33331	**A**	
Menning et al. (2015)—controls	38	50.1 ± 8.7	24.5 ± 3.5	0		27.0 ± 13.7		ELISA	23331	A	
Menning et al. (2015)—breast cancer chemotherapy	32	50.2 ± 9.2	25.8 ± 4.5	0		33.4 ± 26.2		ELISA	33331	A	
Michaud et al. (2016)	675	52.0 ± 15.2	28.2 ± 5.8	36.1		278.2 ± 553.8		ELISA	13331	**A**	**A**
Mwanza et al. (2016)	473	19.3 ± 1.4	31.4 ± 3.8^b^	61.3	7	11.4 ± 3.9^c^	35.0 ± 15.8^c^	LC–MS	12333		
Nery et al. (2018)	16	37.5 ± 5.9	31.1 ± 6.1	0	50			ELISA	33331	A	
O'Brien et al. (2013)	135	30.3 ± 12.8		35		14.5 ± 19.1		ELISA	23131	D	
Olstad et al. (2016)—women	70	43.4 ± 7.2	26.2 ± 6.0	0	18.6	123.7 ± 71.2		ELISA	23321	A	A
Ouellette et al. (2015)—high stress mothers	30	38.2 ± 3.2	25.3 ± 6.0	0		244.6 ± 449.5		ELISA	23321	A	
Ouellette et al. (2015)—low stress mothers	30	37.5 ± 5.2	29.9 ± 8.3	0		126.7 ± 165.4		ELISA	23321	A	
Pickett et al. (2020)	91	24.6 ± 6.5	30.1 ± 7.7	0	42	68.0 ± 161.9		ELISA	13131	AC	AC
Pittner et al. (2020)—adults	171	44.5 ± 14.8	25.8 ± 4.9	25.1	19.3	3.5 ± 5.7	8.6 ± 6.9	LC–MS	21321	AE	AE
Pulopulos et al. (2014)	54	64.8 ± 4.2	26.3 ± 3.5^b^	24.6	11.1	2.4 ± 2.2^b^		LC–MS	21111	**A**	**A**
Qi et al. (2014)	39	30.2 ± 6.1^c^	21.5 ± 2.4	0		24.9 ± 20.0^c^		LC–MS	31311	A	
Radin et al. (2019)	166	42.4 ± 5.1	25.5 ± 5.2	0	17.1	52.9 ± 24.3^b^		ELISA	23121	ACD	ACD
Saleem et al. (2013)—completers	56	66 ± 11	27.3 ± 4.2	85.7		233.2 ± 173.0		ELISA	33111	A	
Saleem et al. (2013)—noncompleters	43	61 ± 11	28.5 ± 5.0	70		153.5 ± 110.5		ELISA	33111	A	
Schalinski et al. (2015)—healthy controls	12	31.9 ± 7.5	22.5 ± 4.1	0	9.1	12.6 ± 11.0		CLIA	12321	A	A
Schalinski et al. (2019)—healthy controls	75	25.4 ± 6.7	23.4 ± 3.6	54.7	5.3	7.3 ± 5.4^b^		CLIA	22111	A	A
Serwinski et al. (2016)	164	43.6 ± 9.8	24.1 ± 4.4	0	10.8	8.4 ± 6.3		LC–MS	21111	A	A
Skoluda et al. (2012)—controls	70	36.6 ± 11.5	23.0 ± 2.5	17.1				CLIA	22321	A	
Skoluda et al. (2012)—endurance athletes	304	38.3 ± 11.6	22.7 ± 2.3	41.1				CLIA	22321	A	
Smith, L. et al. (2019)	3,741	68.4 ± 8.0	28.3 ± 5.3	33.6		26.2 ± 68.8		LC–MS	21131		**A**
Stalder et al. (2010)—nonalcoholic controls	20	43.7 ± 11.2	26.5 ± 3.6	80				CLIA	32331	A	
Stalder et al. (2013)	1,258	39.6 ± 7.3^c^	27.1 ± 3.5^c^	84.8		22.5 ± 11.7^c^	38.5 ± 16.3^c^	LC–MS	21111	**ACDEFG**	
Stalder et al. (2014)—caregivers	20	71.2 ± 6.1	26.7 ± 3.8	5				CLIA	22311	A	
Stalder et al. (2014)—controls	20	72.2 ± 6.4	25.1 ± 3.9	15				CLIA	12311	A	
Stalder, Steudte et al. (2012)—study I	155	24.1 ± 4.2	22.2 ± 3.4	26.5	3.9	17.7 ± 10.6		CLIA	22311	**A**	
Stalder, Steudte et al. (2012)—study II	58	30.5 ± 12.1	24.0 ± 4.9	32.8	12.1	21.6 ± 16.0		CLIA	22311	**A**	
Staufenbiel et al. (2015)	1,425	45.9 ± 13.8		28.2		3.6 ± 2.5^c^	11.1 ± 5.8^c^	LC–MS	11111	**ACEF**	**ACEG**
Steudte et al. (2013)—nontraumatized controls	28	37.6 ± 14.1	23.4 ± 3.05	10.7				LC–MS	11311	A	
Steudte et al. (2013)—traumatized controls	25	41.7 ± 12.3	23.8 ± 3.9	8				LC–MS	21311	A	
Steudte, Kolassa et al. (2011)	17	20.1 ± 5.7	21.4 ± 2.3	64.7				CLIA	22331	A	
Steudte, Stalder et al. (2011)	15	35.7 ± 9.3	22.9 ± 3.5	13.3				CLIA	12311	A	
Steudte‐Schmiedgen et al. (2015)—nontraumatized soldiers	129	26.2 ± 5.2	24.6 ± 2.7	100				LC–MS	21311	A	
Steudte‐Schmiedgen et al. (2017)	17	31.3 ± 9.4^b^	25.4 ± 5.0	11.8		14.1 ± 16.3	19.9 ± 11.3	LC–MS	21321	AE	
Suijker et al. (2018)	15	45.2 ± 15.4	24.9 ± 4.7	43.8	12.5	21.7 ± 14.9		ELISA	31321	A	A
Van Aken et al. (2018)	61	34.8 ± 6.7^b^	25.3 ± 4.6^b^	0		44.4 ± 36.2^b^		ELISA	23321	A	
Van den Heuvel, Stalder et al. (2020)	216	43.8 ± 13.3^b^	31.6 ± 8.1^b^	0	53.7	6.3 ± 5.2^b^ ^,^ ^c^		LC–MS	21111	ACD	ACD
Van den Heuvel, Acker et al. (2020)	164	46.5 ± 15.0	30.5 ± 7.3^c^	0	50.9	6.2 ± 6.4		LC–MS	11111	ACD	ACD
Van den Heuvel, Du Plessis et al. (2020)	56	59.6 ± 8.7	29.5 ± 5.9	0	46.4	5.0 ± 4.5^b^	8.5 ± 6.2	LC–MS	31111	ACDEF**G**	ACDEG
Van der Valk et al. (2020)	51	40.7 ± 12.6	39.7 ± 5.6	27.5	100	5.8 ± 5.3	17.8 ± 13.8	LC–MS	31121	ACEF	ACE**G**
Van Holland et al. (2012)	27	46.2 ± 10.6	26 ± 4	81				ELISA	23331	A	
Van Manen et al. (2019)	32	47.8 ± 8.5^c^	27.8 ± 4.6	43.8	28.1	10.9 ± 11.7	23.9 ± 15.9	LC–MS	31121	ACEF	ACEG
Walther et al. (2016)	271	57.1 ± 10.7	25.4 ± 3.4	100		8.0 ± 6.3	24.6 ± 16.4	LC–MS	11333	**A**D**EG**	
Walton et al. (2013)	10	28 ± 13	27.1 ± 3.6	30				ELISA	33311	A	
Wang et al. (2019)	68	32.5 ± 6.1		0	15	6.3 ± 6.5^c^		LC–MS	21323	A	
Wells et al. (2014)	324	41.9 ± 15.8	27.0 ± 6.5	28.1	24.7	274.4 ± 222.0		ELISA	23311	**A**	**A**
Wester et al. (2014)	47	45 ± 11.3^c^		23.4	100			ELISA	33123	AC	
Wester et al. (2017)	295	46.8 ± 11.7^c^	25.9 ± 4.3	25.4	19.32			LC–MS	11131	AC	A**C**EG
Wester et al. (2017)—healthy controls	174	36.3 ± 8.4^c^	26.8 ± 4.9	42.5				ELISA	23331	A	
Wu et al. (2019)	160	45.7 ± 9.8	26.6 ± 3.1	55.4	31	23.4 ± 30.5		ELISA	33121	A	A
Younge et al. (2015)	151	41.3 ± 14.2	25.5 ± 4.9	37.1				ELISA	33111	**A**	**A**
Zai et al. (2017)	248								13333	**A**	
Zekas et al. (2019)	81	36.5 ± 6.2		100				LC–MS	21131	**C**	
**Pediatric cohorts**											
Bryson et al. (2020)	297	3.1 ± 0.1	16.8 ± 1.8	39.4	22.9	8.5 ± 7.8		ELISA	23131	A	A
Chen et al. (2015)—female adolescents	47	15.8 ± 3.1	24.3 ± 5.2	0		3.4 ± 1.9		LC–MS	21121	AD	
Chen et al. (2015)—male adolescents	32	15.0 ± 2.1	21.7 ± 4.5	100		4.0 ± 2.5		LC–MS	21121	AD	
Condon et al. (2019)	45	6.8 ± 2.1	0.7 ± 1.2		22.2	57.3 ± 112.7		ELISA	23131	B	B
De Kruijff et al. (2020)	278	10.8 ± 4.6	−0.1 ± 1.0	51.1	0.8	3.1 ± 3.1		LC–MS	21311	**AB**	**AB**
Distel et al. (2019)	52	8.4 ± 1.3	20.8 ± 4.4	39	29.3	20.6 ± 63.4		ELISA	23121	**A**	**A**
Evans et al. (2019)	92	10.1 ± 0.3	17.3 ± 2.1	34.8		3.0 ± 4.5	10.1 ± 12.0	LC–MS	11121	AE	A
Föcker et al. (2016)	20	17.3 ± 1.0	−0.3 ± 1.1	0		12.6 ± 9.7		CLIA	12121	B	
Frisch et al. (2020)	18	7.4 ± 1.0	15.8 ± 2.4	44	0	2.8 ± 2.4		ELISA	33321	A	
Gao et al. (2014)—young male control	29	16.7 ± 0.6	21.4 ± 2.4	100	0	13.9 ± 10.9		LC–MS	22321	A	
Gao et al. (2014)—young male earthquake survivor	20	16.8 ± 0.8	21.7 ± 2.4	100	0	25.3 ± 17.1		LC–MS	22321	A	
Genitsaridi et al. (2019)	300	10.5 ± 2.6	25.7 ± 5.4^b^	25.3	46.7	8.9 ± 1.0^b^		CLIA	32131	ACD	
Gerber et al. (2017)	318	7.3 ± 3.5	16.3 ± 2.2	46.9	8	12.2 ± 9.7		CLIA	12111	**AC**	**AC**
Golub et al. (2019)	137	7.6 ± 0.6	16.1 ± 1.8	47.5	0			ELISA	13113	A	
Grunau et al. (2013)—full term	42	7.8 ± 0.8	16.8 ± 3.2	35.7		416.2 ± 873.0		ELISA	33311	A	
Grunau et al. (2013)—pre‐term	91	7.7 ± 0.3	15.7 ± 2.4	46.2		301.2 ± 560.8		ELISA	33311	A	
Hu et al. (2017)	1,263	8.0 ± 0.8		47.3		11.8 ± 1.9^b^		ELISA	13123	**A**	
Ilg et al. (2020)	134	12.0 ± 4.0	18.6 ± 3.7	57		3.7 ± 2.3	13.4 ± 7.2	LC–MS	31323	**A**E	
Ince‐Askan et al. (2019)	117	9.8 ± 2.4^b^	0.4 ± 1.1^b^	59.8	7.7	1.3 ± 1.0^b^	7.4 ± 3.5^b^	LC–MS	21131	AE	ABCDEFG
Kamps et al. (2014)	10	10.5 ± 1.3	0.1 ± 1.0	50	10	4.8 ± 4.0		LC–MS	11121	AB	AB
Larsen et al. (2016)—children	363	5.4 ± 1.07	16.1 ± 1.2	55		146.5 ± 179.0		ELISA	23131	A	BCD
Lehto et al. (2018)	599	4.7 ± 0.9	15.9 ± 1.4	52	2.1	41 ± 77		CLIA	12131	C	C
Ling et al. (2020)—children	35	4.7 ± 0.8	0.7 ± 1.0	51.4	20	32.0 ± 45.4		ELISA	23111	B	AB
Michels et al. (2017)	81	12.7 ± 1.7	−0.03 ± 0.9	53.6	0	25 ± 5		LC–MS	11121	A	AB
Murray et al. (2016)	54	9.5 ± 0.3	17.6 ± 2.3	20.3	0	3.2 ± 2.9^b^		ELISA	33131	A	A
Olstad et al. (2016)—children	30	14.3 ± 3.9	0.3 ± 1.1	56.7	10	96.6 ± 49.6		ELISA	23121	B	B
Ouellet‐Morin et al. (2016)	34	17		26.5		33.0 ± 24.5		ELISA	13331	A	A
Ouellette et al. (2015)—high stress daughters	30	7.5 ± 0.7	15.3 ± 2.2	0		89.9 ± 235.1		ELISA	23321	A	
Ouellette et al. (2015)—low stress daughters	30	7.7 ± 0.7	15.6 ± 2.8	0		104.4 ± 218.3		ELISA	23321	A	
Panter‐Brick et al. (2019)	203	14.4 ± 1.7	0.0 ± 1.0	56.9	5.3	9.5 ± 10.0		ELISA	23121	**A**	**A**
Papafotiou et al. (2017)—normal weight	25	7.8 ± 1.2	−0.03 ± 0.6	0		1.2 ± 0.6		LC–MS	11121	AB	A
Papafotiou et al. (2017)—obesity	25	7.4 ± 1.3	2.9 ± 1.4	0	100	4.1 ± 5.0		LC–MS	31121	**A**	A**B**
Petimar et al. (2020)—mid‐childhood	599	7.9 ± 0.8	0.3 ± 1.0	45.9	8.8	1.3 ± 1.5^c^		LC–MS	11121	**AC**	**ABC**
Pittner et al. (2020)—children	61	12.4 ± 3.2	0.2 ± 1.0	42.6	3.3	1.6 ± 1.5	5.7 ± 2.9	LC–MS	21321	BE	BE
Pyle Hennessey et al. (2020)	100	5.8 ± 0.3	15.6 ± 1.7	48	3.1	6.8 ± 6.9		ELISA	23121	A	A
Schloss et al. (2018)	75	4.6 ± 0.3		41.3				ELISA	23312	A	
Slopen et al. (2018)	344	2.1 ± 0.1	17.5 ± 1.7	43.2	11.1	19.0 ± 42.4		ELISA	13121	A	A
Smith, J. et al. (2019)	114	8.5 ± 0.3	17.0 ± 2.6	42.1		4.2 ± 4.1		ELISA	23311	AC	
Sun et al. (2018)	1,000	9.0 ± 0.9	18.6 ± 3.2	42.1		12.0 ± 2.0^c^		ELISA	13131	**A**	**A**
Van Dammen et al. (2020)	181	15.7 ± 2.0	20.3 ± 3.2	38.9	1.6	3.5 ± 2.1		LC–MS	12331	**A**B	**A**B
Vehmeijer et al. (2020)	2042	6.1 ± 0.6	0.2 ± 0.9	47.5	3.6	1.9 ± 1.4	9.6 ± 7.4	LC–MS	11121	**AE**	**A**
Vepsäläinen et al. (2021)	565	4.8 ± 0.9	15.9 ± 1.5	37.9	2.1	40.9 ± 77.1		CLIA	12131	A	A
Wagner et al. (2019)	434	12.0		38.5	9.9			LC–MS	11112		B
White et al. (2017)	537	10.0 ± 3.1^b^	0.0 ± 0.8	49.3				CLIA	22322	**B**	

*Note*: Reported bivariate correlation/regression coefficient: A, HairF versus BMI; B, HairF versus BMI SDS; C, HairF versus WC; D, HairF versus WHR; E, HairE versus BMI; F, HairE versus WC; G, HairE versus WHR. Significant associations are represented in bold.

Abbreviations: CI, confidence interval; HairF, hair cortisol; HairE, hair cortisone; SDS, standard deviation score; WC, waist circumference; WHR, waist‐to‐hip ratio; LC–MS, liquid chromatography‐(tandem) mass spectrometry; ELISA, enzyme‐linked immunosorbent assay; CLIA, chemiluminescent immunoassay.

^a^ Risk of bias: 1, low risk of bias; 2, moderate risk of bias; 3, high risk of bias. Each number represents the assessed QUIPS domains. The following definitions were used for low, moderate of high risk of bias: QUIPS 1 (study participation), 1: population‐based sampling, 2: population selection on nonmedical or social conditions, 3: population selection on medical conditions not evidently related to disturbances of the HPA‐axis; QUIPS 2 (study attrition), not applicable and therefore not scored; QUIPS 3 (prognostic factor measurement), 1: HairGC analysis using LC–MS, 2: HairGC analysis using CLIA, 3: HairGC analysis using ELISA; QUIPS 4 (outcome measurement), 1: anthropometric measurements objectively measured, 2: anthropometric measurements self‐reported; QUIPS 5 (study confounding), 1: both outliers and corticosteroid use taken into account, 2: only outliers or only corticosteroid use taken into account, 3: outliers and corticosteroid use both not taken into account; QUIPS 6 (statistical analysis and reporting), 1: relevant statistics fully reported, 2: relevant statistics partly reported, 3: relevant statistics not reported.

^b^ Pooled subgroup means.

^c^ Means calculated from either median and interquartile range, or from median and range.

### 3.1 Description of study characteristics

The weighted mean age of cohorts involving adults (available for *n* = 23,467) was 53.3 ± 18.4 years and weighted mean BMI (*n* = 19,653) was 27.0 ± 5.4 kg/m^2^. For studies involving children, weighted mean age (*n* = 9904) was 7.8 ± 3.3 years and weighted mean BMI SDS (*n* = 4108) was 0.2 ± 1.0. Forty‐three of the 146 cohorts (29%) included children (mean age <18 years). The majority of the cohorts had a population that was predominantly female (104 cohorts had >50% females), although the proportion of females within all included subjects was 44%.

Of the 43 pediatric cohorts, two specifically included only children with obesity,^63^, ^99^ whereas the other 41 cohorts either had no criteria regarding weight status or included only children with normal weight. In adults, 2 of the 103 cohorts exclusively included adults with obesity (BMI ≥ 30 kg/m^2^),^131^, ^141^ whereas the other 101 cohorts either had no criteria regarding weight status or included only adults with normal weight or overweight. In 12 of the 103 adult cohorts (12%), the mean BMI of the included population was 30 kg/m^2^ or higher. Details on the mean BMI of the studies can be found in Table 1.

BMI was the most commonly reported obesity measurement in 138/146 cohorts (95%), followed by WC in 30/146 cohorts (21%), WHR in 20/146 cohorts (14%), and BMI SDS in 16/43 pediatric cohorts (37%).

For 145 cohorts (99%) the used laboratory method was reported, which were ELISA (63/145 cohorts, 43%), LC–MS or LC–MS/MS (56/145 cohorts, 39%), or CLIA (26/145 cohorts, 18%). In all cohorts HairF was reported, whereas HairE was additionally reported in 19/146 cohorts (13%).

Mean crude HairGC concentrations across the studies varied widely with reported means ranging from 1.2–592.2 pg/mg for HairF and 2.45–38.48 pg/mg for HairE. Mean HairF concentrations were higher in studies that used an ELISA (weighted mean 95.6 ± 236.4 pg/mg) compared with studies that used CLIA (24.0 ± 45.1 pg/mg) or LC–MS (mean 13.4 ± 13.4 pg/mg and mean 12.2 ± 39.5 pg/mg in a sensitivity analysis without Mazgelyte et al*.,* ^86^ which was a significant outlier in mean HairF level). All HairE analyses except for one^78^ were performed using LC–MS. In the studies that reported both HairE and HairF concentrations, HairE levels in most cases were higher than HairF levels (Table 1).

### 3.2 Risk of bias

Risks of bias assessments on cohort level are presented in Table 1. With respect to the selection of the population domain (QUIPS 1), 25 (17%) cohorts had a high, 75 (52%) medium, and 46 (31%) low risk of bias. Regarding the prognostic factor (HairGC) measurement domain (QUIPS 3), 65 (45%) cohorts had a high, 31 (21%) medium, and 50 (34%) low risk of bias. For the outcome measurement domain (QUIPS 4), 75 (51%) cohorts had a moderate and 71 (49%) a low risk of bias. In the domain of accounting for possible confounders (QUIPS 5), 37 (25%) cohorts had a high, 64 (44%) medium, and 45 (31%) low risk of bias. With regard to the statistical domain (QUIPS 6), 10 (7%) cohorts had a high, 4 (3%) medium, and 132 (89%) low risk of bias.

### 3.3 Qualitative synthesis

An overview of all outcomes reporting any relation between HairGC and obesity measurements is shown in the supporting information Table S1.

### 3.4 Quantitative synthesis

#### 3.4.1 Meta‐analysis of correlation coefficients

In total, 140/146 cohorts (96%) from 115 unique studies were included in the meta‐analyses of correlations, comprising data of 28,830 participants. The pooled correlation coefficients ranged from 0.10–0.18 (all *p* < 0.0001). The strongest pooled correlation was found for HairE versus WC (pooled *r* = 0.18; Table 2 and supporting information Figures S1–S6). Meta‐regressions and subgroup analyses were possible for the associations between HairF versus BMI, BMI SDS, WC, and WHR and HairE versus BMI. In subgroup analyses, neither applied laboratory methods nor population‐based sampling moderated the correlations between HairGC and obesity measurements (all *p*‐values >0.05, Table 3). Subgroup analyses on all QUIPS domains showed no moderation by risk of bias categories except for QUIPS domain 4 (assessment of outcome, that is, self‐reported BMI vs. measured): studies with self‐reported BMI showed stronger correlations with HairF than studies with measured BMI (pooled *r* of 0.15 vs. 0.07, respectively; *Q* = 14.34, *p* < 0.0001).

**TABLE 2:** Pooled correlation coefficients

	*k* cohorts	*n* participants	Pooled *r*	95% CI	95% PI	*P*‐value	Between‐study heterogeneity		
							*I* ^2^ (%)	*Q*	*P*‐value
HairF versus BMI	122	26,527	0.10	0.08; 0.13	−0.04; 0.24	<0.0001	51.2	221.4	<0.0001
HairF versus BMI SDS	11	1,247	0.12	0.06; 0.18	0.06; 0.18	<0.0001	0.0	11.8	0.30
HairF versus WC	24	11,006	0.11	0.07; 0.15	−0.03; 0.26	<0.0001	68.3	59.7	<0.0001
HairF versus WHR	16	6,786	0.11	0.07; 0.15	0.03; 0.19	<0.0001	28.4	22.3	0.10
HairE versus BMI	16	8,210	0.11	0.07; 0.15	0.00; 0.21	<0.0001	52.7	31.0	0.01
HairE versus WC	7	3,158	0.18	0.11; 0.24	0.06; 0.29	<0.0001	45.7	9.6	0.14
HairE versus WHR	2	1,314	NA^a^	NA	NA	NA	NA	NA	NA

Abbreviations: CI, confidence interval; HairF, hair cortisol; HairE, hair cortisone; NA, not applicable; PI, prediction interval; SDS, standard deviation score; WC, waist circumference; WHR, waist‐to‐hip ratio.

^a^ Meta‐analysis not performed due to small number of cohorts.

**TABLE 3:** Results of subgroup analyses in the meta‐analyses of correlation coefficients

	Moderator	*k* cohorts	*I* ^2^ (%)	Pooled *r*	95% CI	*Q*_between_	*P*‐value
**HairF versus BMI**	QUIPS 1: Study participation (population‐based sampling)					0.34	0.55
	Yes	34	51	0.10	0.07; 0.13		
	No	88	51	0.11	0.08; 0.14		
	QUIPS 3: Prognostic factor measurement (HairGC analysis method)					0.05	0.98
	LC–MS	47	51	0.10	0.07; 0.14		
	ELISA	52	35	0.10	0.07; 0.14		
	CLIA	21	66	0.11	0.05; 0.17		
	**QUIPS 4: Outcome (anthropometric) measurement**					**14.34**	**<0.001**
	**Self‐reported**	**67**	**22**	**0.15**	**0.12; 0.18**		
	**Objectively measured**	**55**	**62**	**0.07**	**0.04; 0.10**		
	QUIPS 5: Study confounding					2.74	0.43
	CS use and outliers handled	39	62	0.13	0.09; 0.17		
	Only outliers handled	22	29	0.09	0.05; 0.13		
	Only CS use handled	33	30	0.10	0.06; 0.15		
	Neither handled	28	51	0.08	0.03; 0.12		
	QUIPS 6: Statistical analysis (Relevant statistics fully reported)					0.01	0.93
	Yes	118	50	0.10	0.08; 0.13		
	No	4	65	0.10	−0.04; 0.23		
**HairF versus BMI SDS**	QUIPS 1: Study participation (population‐based sampling)					0.12	0.73
	Yes	4	0	0.14	0.01; 0.27		
	No	7	0	0.12	0.05; 0.18		
	QUIPS 3: Prognostic factor measurement (HairGC analysis method)					0.63	0.73
	LC–MS	6	70.7	0.06	−0.13; 0.25		
	ELISA	3	0	0.07	−0.13; 0.26		
	CLIA	2	0	0.13	0.04; 0.21		
	QUIPS 4: Outcome (anthropometric) measurement					2.11	0.15
	Self‐reported	4	0	0.14	0.08; 0.20		
	Objectively measured	7	32.1	−0.01	−0.19; 0.18		
	QUIPS 5: Study confounding					0.86	0.83
	Both handled	2	0	0.13	0.03; 0.24		
	Only outliers handled	2	0	0.13	0.04; 0.21		
	Only CS use handled	5	60.8	−0.01	−0.31; 0.28		
	Neither handled	2	0	0.13	0.00; 0.26		
**HairF versus WC**	QUIPS 1: Study participation (population‐based sampling)					**3.95**	**0.05**
	Yes	9	65	0.07	0.02; 0.13		
	No	15	60	0.15	0.09; 0.20		
	QUIPS 3: Prognostic factor measurement (HairGC analysis method)					0.17	0.92
	LC–MS	12	78	0.11	0.05; 0.18		
	ELISA	7	4	0.10	0.02; 0.17		
	CLIA	5	77	0.11	0.02; 0.21		
	QUIPS 4: Outcome (anthropometric) measurement					0.67	0.41
	Self‐reported	3	40	0.18	0.01; 0.35		
	Objectively measured	21	71	0.11	0.06; 0.15		
	QUIPS 5: Study confounding					5.90	0.12
	Both handled	9	68	0.08	0.02; 0.15		
	Only outliers handled	3	0	0.16	0.13; 0.19		
	Only CS use handled	7	33	0.13	0.05; 0.21		
	Neither handled	5	77	0.10	−0.03; 0.23		
**HairF versus WHR**	QUIPS 1: Study participation (population‐based sampling)					0.56	0.46
	Yes	4	57	0.15	0.03; 0.26		
	No	12	36	0.10	0.04; 0.15		
	QUIPS 3: Prognostic factor measurement (HairGC analysis method)					0.34	0.56
	LC–MS	11	33	0.10	0.05; 0.15		
	ELISA	4	76	0.16	−0.03; 0.34		
	**QUIPS 4: Outcome (anthropometric) measurement**					**5.79**	**0.02**
	**Self‐reported**	**2**	**0**	**0.36**	**0.16; 0.53**		
	**Objectively measured**	**14**	**36**	**0.10**	**0.06; 0.14**		
	QUIPS 5: Study confounding					2.85	0.24
	Both handled	6	53	0.09	0.02; 0.15		
	Only outliers handled	5	0	0.13	0.10; 0.16		
	Only CS use handled	4	57	0.23	0.06; 0.38		
	Neither handled	1	NA	NA	NA		
**HairE versus BMI**	QUIPS 1: Study participation (population‐based sampling)					0.02	0.89
	Yes	6	40	0.11	0.07; 0.15		
	No	9	46	0.12	0.02; 0.21		
	QUIPS 4: Outcome (anthropometric) measurement					0.24	0.62
	Self‐reported	3	78	0.22	−0.20; 0.57		
	Objectively measured	12	59	0.12	0.07; 0.16		
	**QUIPS 5: Study confounding**					**8.08**	**0.04**
	Both handled	4	55	0.16	0.11; 0.21		
	Only outliers handled	4	0	0.07	0.04; 0.11		
	Only CS use handled	5	0	0.09	−0.02; 0.20		
	Neither handled	2	61	0.05	−0.12; 0.21		

*Note*: Subgroup analyses were only performed when data of at least 2 cohorts were available within a subgroup and 10 cohorts across all subgroups. Bold text indicates statistically significant effect (*P*‐value < 0.05).

Abbreviations: CI, confidence interval; HairF, hair cortisol; SDS, standard deviation score; WC, waist circumference; WHR, waist‐to‐hip ratio; LC–MS, liquid chromatography‐(tandem) mass spectrometry; ELISA, enzyme‐linked immunosorbent assay; CLIA, chemiluminescent immunoassay.

In meta‐regressions, we found that studies that included larger proportions of males showed stronger correlations between HairF and WC (estimated slope 0.0022 per percentage point increase in proportion of males, 95% CI 0.0010 to 0.0033, *p* = 0.0002) and HairF and WHR (estimated slope 0.0011 per percentage point increase in proportion of males, 95% CI 0.0001 to 0.0021, *p* = 0.02; Table 4 and supporting information Figures S7 and S8). Furthermore, studies including more participants with obesity showed weaker correlations between HairF and BMI (estimated slope −0.0029 per percentage point increase in proportion of participants with obesity, 95% CI −0.0049 to −0.0010, *p* = 0.0028), and studies with higher BMI SDS showed weaker correlations between HairF and BMI SDS (Table 4 and supporting information Figure S9). Mean age and mean HairF concentration of the study population did not moderate the correlations between HairGC and obesity measurements (all *p*‐values >0.05, Table 4). In contrast, higher mean HairE was associated with stronger positive correlations (estimated slope 0.0046 per point increase in mean HairE on study level, 95% CI 0.0025–0.0068, *p* < 0.0001). Visual inspection of the funnel plots showed no evidence for publication bias; that is, no systematic trends were found between standard error (as proxy for study sample size) and magnitude and direction of the reported correlation coefficients (supporting information Figures S10–S15).

**TABLE 4:** Results of meta‐regressions in the meta‐analyses of correlation coefficients

	Moderator	*k* cohorts	% Between‐study heterogeneity explained	Estimate (slope)	95% CI	*Q*_*m*_	*P*‐value
**HairF versus BMI**	Mean age	120	0.3	0.0006	−0.0005; 0.0017	1.32	0.25
	Mean BMI	113	0.7	0.0003	−0.0050; 0.0057	0.01	0.90
	Adults only	84	0.7	−0.0082	−0.0180; 0.0016	2.70	0.10
	Mean HairF	115	0.002	0.0000	−0.0002; 0.0003	0.10	0.76
	LC–MS	44	2.1	0.0008	−0.0042; 0.0057	0.09	0.76
	CLIA	23	7.4	−0.0025	−0.0092; 0.0041	0.55	0.46
	ELISA	47	0.03	0.0000	−0.0003; 0.0003	0.02	0.88
	**% obesity**	**57**	**11.9**	**−0.0029**	**−0.0049; −0.0010**	**8.95**	**0.0028**
	% males	122	2.5	0.0003	−0.0006; 0.0011	0.38	0.54
**HairF versus BMI SDS**	Mean age	11	11.0	0.0127	−0.0091; 0.0344	1.30	0.25
	% males	10	18.6	0.0037	−0.0012; 0.0087	2.18	0.14
	**Mean BMI SDS**	**10**	**86.4**	**−0.2108**	**−0.3408; −0.0807**	**10.09**	**0.0015**
	Mean HairF	10	1.03	−0.0006	−0.0040; 0.0028	0.12	0.73
**HairF versus WC**	Mean age	23	21.9	0.0011	−0.0007; 0.0028	1.46	0.23
	Mean BMI	20	9.3	0.0013	−0.0081; 0.0106	0.07	0.79
	Adults only	17	18.3	−0.0080	−0.0267; 0.0108	0.69	0.41
	Mean HairF	21	0.002	0.0003	−0.0006; 0.0012	0.46	0.50
	% obesity	16	0.03	−0.0002	−0.0030; 0.0027	0.02	0.89
	**% males**	**23**	**39.5**	**0.0022**	**0.0010; 0.0033**	**14.29**	**0.0002**
**HairF versus WHR**	Mean age	15	10.7	0.0024	−0.0006; 0.0055	2.10	0.12
	Mean BMI	12	13.3	−0.0120	−0.0315; 0.0074	1.47	0.23
	Adults only	10	54.4	−0.0170	−0.0377; 0.0037	2.59	0.11
	Mean HairF	14	4.0	0.0014	−0.0020; 0.0047	0.65	0.42
	LC–MS	11	25.2	0.0056	−0.0013; 0.0126	2.53	0.11
	**% males**	**15**	**28.7**	**0.0011**	**0.0001; 0.0021**	**5.07**	**0.02**
**HairE versus BMI**	Mean age	15	27.9	0.0016	−0.0004; 0.0035	2.56	0.11
	Mean BMI	12	47.2	0.0096	−0.0006; 0.0197	3.41	0.07
	**Mean HairE**	**13**	**65.2**	**0.0046**	**0.0025; 0.0068**	**17.96**	**<.0001**
	% males	15	12.2	0.0010	−0.0010; 0.0030	0.99	0.32

*Note*: Meta‐regressions were only performed when data of at least 10 cohorts were available. Bold text indicates statistically significant effect (*P*‐value < 0.05).

Abbreviations: CI, confidence interval; Q_m_, Cochrane's Q for the moderator; HairF, hair cortisol; SDS, standard deviation score; WC, waist circumference; WHR, waist‐to‐hip ratio; LC–MS, liquid chromatography‐(tandem) mass spectrometry; ELISA, enzyme‐linked immunosorbent assay; CLIA, chemiluminescent immunoassay; NA, not available or not applicable.

#### 3.4.2 Meta‐analysis of regression coefficients

The pooled regression coefficients stratified on analysis method are presented in Table 5. The pooled regression coefficient for 10‐log transformed HairF as independent variable on BMI as dependent variable measured for LC–MS‐based measurements was based on the largest number of cohorts (*k* = 26 cohorts comprising 11,635 individuals). The pooled regression coefficient for LC–MS‐based measurements was 0.049 kg/m^2^ (95% CI 0.045–0.054; Table 5). This indicates that for LC–MS‐based measurements, 1 point increase in 10‐log HairF was associated with 0.049 kg/m^2^ higher BMI. One point increase in 10‐log HairE was associated with 1.15 kg/m^2^ higher BMI (95% CI 0.987–1.310 kg/m^2^). The highest pooled regression coefficient was found for HairE on dependent variable WC, where 1 point increase in 10‐log HairE was associated with 11.0 cm larger WC (95% CI 10.1–11.9 cm) on LC–MS. There was no significant between‐study heterogeneity (all p‐values >0.05, Table 5).

**TABLE 5:** Pooled regression coefficients

	*k* cohorts	*n* participants	Analysis method	Pooled beta	95% CI	Between‐study heterogeneity	
						*Q*_*w*_	*P*‐value
HairF independent—BMI dependent	8	1,984	**CLIA**	**0.02**	**0.016; 0.03**	0.26	>0.05
	26	11,635	**LC–MS**	**0.05**	**0.045; 0.054**	0.50	>0.05
HairF independent—BMI SDS dependent	‐	‐	CLIA	‐	‐		
	6	998	**LC–MS**	**0.20**	**0.14; 0.27**	0.11	>0.05
HairF independent—WC dependent	4	1,556	**CLIA**	**0.02**	**0.02; 0.03**	0.13	>0.05
	10	4,259	**LC–MS**	**1.26**	**1.08; 1.44**	0.15	>0.05
HairF independent—WHR dependent	‐	‐	CLIA	‐	‐		
	5	1,805	LC–MS	−0.01	−0.01; −0.00	0.00	>0.05
HairE independent—BMI dependent			CLIA	‐	‐		
	9	5,266	**LC–MS**	**1.15**	**0.98; 1.31**	0.08	>0.05
HairE independent—WC dependent			CLIA	‐	‐		
	6	3,102	**LC–MS**	**11.0**	**10.1; 11.9**	0.05	>0.05

*Note*: ‐, meta‐analysis not performed due to insufficient number of cohorts. Bold text indicates statistically significant effect (*P*‐value < 0.05).

Abbreviations: CI, confidence interval; HairF, hair cortisol; HairE, hair cortisone; SDS, standard deviation score; WC, waist circumference; WHR, waist‐to‐hip ratio; LC–MS, liquid chromatography‐(tandem) mass spectrometry; CLIA, chemiluminescent immunoassay.

## 4 DISCUSSION

In the current systematic review including 34,342 unique subjects, HairGC levels showed a significant positive relation with anthropometric measurements. In the meta‐analyses, pooled correlation coefficients ranged between 0.10 for hair cortisol versus BMI and 0.18 for hair cortisone versus WC. The largest effect size was found for the relation between hair cortisone and WC: one point increase in 10‐log‐transformed hair cortisone concentration (e.g., an increase from 1 pg/mg to 10 pg/mg) on LC–MS‐based assays was associated with 11 cm larger WC. For the outcome BMI, an increase of 1.15 kg/m^2^ per one point increase in 10‐log transformed hair cortisone on LC–MS‐based assays was found. Moderator analysis in the meta‐analyses of correlation coefficients showed that a higher percentage of male participants was associated with stronger correlations in the relations between hair cortisol versus WC and hair cortisol versus WHR. A higher percentage of participants with obesity of the included cohorts was associated with less strong correlations in the relation hair cortisol versus BMI. Interestingly, no evidence was found for a moderating influence on study level of other important covariates that are known to influence either HairGC or obesity measurements in individual persons, namely age, laboratory methods, and handling of outliers and exogenous corticosteroid use.

In the largest of our meta‐analyses, for HairF versus BMI (*n* = 26,527 participants), we confirmed the modest positive relations in exploratory analyses of Stalder et al. and Ling et al. between HairF and BMI/BMI SDS.^9^, ^12^ Evidently, there is a relation between measures of obesity and long‐term glucocorticoid levels, a relation that has been controversial for measurement of GC levels in other matrices that reflect shorter time periods.^6^ As GC are known to contribute to central adiposity, for example, in Cushing's syndrome, it might be possible that in the study of a gradually developing disease such as obesity, long‐term GC measurements offer a different and perhaps more appropriate perspective to the role of the HPA‐axis.

The current study indicates that this relation is strongest (i.e., the highest correlation coefficient and the largest effect size) for cortisone, the inactive form of cortisol, and WC. Although the pooled correlation coefficients and pooled regression coefficients for the most frequently studied outcome HairF versus BMI were statistically significant (pooled correlation coefficient 0.10, pooled regression coefficient 0.049 kg/m^2^ increase in BMI per 1 point increase in 10‐log transformed HairF on LC–MS), the small effect size here seems to have less clinical relevance compared with the large effect size we found for the relation HairE versus WC. We believe that the consistency of our findings across all studied outcomes is indicative of an altered setpoint of the HPA‐axis in obesity. This may induce or aggravate obesity, although causality cannot be proven by our study because of its limitation to cross‐sectional associations. Yet, the fact that HairGC apparently relate strongly to measures of abdominal obesity matches the paradigm that chronic exposure to higher levels of GCs specifically induce abdominal obesity.^18^ Importantly, specifically abdominal obesity increases mortality, for example, by compromising cardiometabolic health and increasing the risk of many chronic diseases.^147^

Previous meta‐analyses already demonstrated an overall relation between HairF and BMI. However, this was investigated in smaller groups that also included individuals with psychosocial or biological factors affecting the HPA‐axis such as post‐traumatic stress disorder,^9^ or limited to children only.^12^ Therefore, another important aim of our study was to identify moderators and subgroups within this relation on study level. This could improve the eventual applicability of HairGC measurements in the context of weight variability and additionally increase our understanding of the underlying biological mechanisms.

Strikingly, the pooled correlations between parameters of obesity and cortisone, the inactive form of cortisol, tended to be stronger than the relations with cortisol itself. The equilibrium between cortisol and cortisone is controlled by the enzymes 11β‐hydroxysteroid dehydrogenase types 1 and 2 both in the circulation (which is mostly determined by hepatic enzyme activity) as well as at tissue level, differing per tissue type.^148^ With regard to scalp hair, it has been suggested that human hair follicles display a functional equivalent of the HPA‐axis and can synthesize cortisol,^149^ although this finding has until now not been confirmed by others. However, there are currently no reports regarding balance between cortisol and cortisone at the shaft level. Therefore, it is believed that at least HairF represents cumulative circulating levels of cortisol,^150^ which presumably also holds true for HairE and cortisone. Perhaps this more stable circulating “reservoir” of inactive cortisol can be seen as a better indicator of chronic hypercortisolism related to adiposity, considering the stronger relations that we found for HairE. Moreover, this matches previous findings that HairE has a better diagnostic efficacy than HairF in the diagnostic screening for endogenous hypercortisolism.^4^

Furthermore, in contrast to Ling et al.,^12^ our meta‐analyses did not indicate that LC–MS‐based cortisol measurements had a stronger relation to obesity than ELISA or CLIA‐based measurements. In principle, the LC–MS‐based method has a higher specificity than the ELISA method because it mostly lacks the interference from other steroid compounds.^151^ The finding that LC–MS‐based studies did not show a higher correlation for cortisol and obesity measurements than ELISA‐based studies could also point towards an actual biological effect that in obesity, there is a more general activation of the HPA‐axis. This general activation could lead to increased levels of other steroid hormones such as cortisone, which could potentially reduce issues associated with cross‐reactivity in this context.

The percentage of males included was a significant influencer of the relation between WC and HairF, with a similar trend for WHR and HairF, but not for HairF and BMI. For both WC and WHR, cut‐off values are sex‐specific, with males generally having a larger WC and WHR than females. This might contribute to the stronger associations between HairGC and anthropometric measurements in studies that contain more males. Unfortunately, lack of raw data hampered stratification for sex.

We also observed that studies that had a high percentage of participants with obesity found less strong associations between HairF and BMI. Although HairGC levels may explain less of the weight variability in cohorts with individuals with obesity compared with cohorts that include wider weight ranges, it has clearly been established that individuals with obesity in general have higher HairGC than individuals without obesity,^14^, ^141^, ^152^ an observation that is confirmed by our current analyses. It might be possible that within individuals with obesity, HairGC relate more to metabolic health than to anthropometrics per se. Another explanation could be the presence of a certain “tipping point,” perhaps the development of hepatic steatosis, that may interfere with cortisol‐metabolizing enzymes, leading to or maintaining the state of hypercortisolism.

In contrast to our expectations, we found that studies using self‐reported BMI reported stronger correlations to HairGC levels than studies using objective anthropometric features (*r* = 0.15 and *r* = 0.07, respectively, for HairF‐BMI). One possible explanation for this finding could include higher perceived weight stigma in individuals with obesity. Weight stigma is associated with adverse psychological consequences, such as anxiety, lower self‐esteem, poor quality of life, as well as with higher HairF levels.^153^ When perceived weight stigma would cause individuals with obesity to overestimate their own weight, this could result in stronger correlations between BMI and HairGC levels, although this is highly speculative. Other possible areas of bias, for example, the selection of participants (whether or not the participant selection was population‐based or based on medical, occupational or socio‐economic characteristics), the consideration of possible confounders (outliers of HairGC measurements and corticosteroid use), and the statistical reporting all did not affect the outcomes.

As expected, given the large number of included studies, we observed a relatively high between‐study heterogeneity in our meta‐analyses of correlation coefficients, up to an *I* ^2^ of 68% for HairF versus WC. Although some of our studied moderators could explain part of this heterogeneity, the majority is still unexplained. Hence, there may be a role for other factors that are known to influence HairGC levels and/or obesity that we did not account for in the current report. For example, a recent meta‐analysis demonstrated that adversity also relates to long‐term GC levels, although this relation is complex and depends on the type and timing of adversity and on the studied population.^154^ Adversity and stressful conditions can have similar complex relations to obesity.^155^ We did not include these factors as possible moderators in our analyses due to a lack of universally accepted definitions that we could apply to all studies. However, we do not suspect a major influence of stressful conditions on our results as sensitivity analyses focusing on population‐based cohorts were comparable with the analyses based on all data.

A major strength of the current study was our comprehensive search in which we included all studies that reported any association between measures of adiposity and HairGC levels, including studies that did not primarily aim to investigate these associations. To minimize the risk of publication bias due to incomplete reporting of results based on statistical significance, we contacted corresponding authors of all included studies for additional information. In addition, we contacted all corresponding authors of studies that reported anthropometric measurements and HairGC but not an association. This yielded additional information for 70 cohorts (48%). This limits the risk of publication bias, which was also confirmed by our funnel plots (supporting information Figures S10–S15). Moreover, an important addition of our work compared with the two systematic reviews and meta‐analyses that have already been published on this topic was that we studied both the active form cortisol and the inactive form cortisone, their relations to different measures of adiposity, and also investigated effect sizes complementary to correlations. This has yielded the valuable conclusion that both the strongest correlation as well as the strongest, clinically relevant effect size are actually seen for HairE versus WC, instead of the most commonly studied association HairF versus BMI. Another strength of our study is that we focused on studies that did not include participants with severe diseases affecting GC levels, which have therefore not disturbed our findings.

A limitation of our study was that we obtained data that are related to full cohorts instead of individual person‐data. This restricts our conclusions to comparisons across cohorts instead of across individuals. However, by pooling regression coefficients, we could provide an effect size that is applicable on individual level. Other limitations relate to the lack of standardization of HairGC analysis methods and the usefulness of HairGC itself, as there are still numerous issues unsolved. For example, the ubiquitously reported growth speed of scalp hair, 1 cm per month, may vary considerably by ethnicity and season.^8^ Other issues represent the high prevalence of overall CS use (which may influence basal cortisol levels and were found to be used by 11% of the Dutch population, a number that may be even higher in other countries^140^, ^156^), hair characteristics such as color, treatment and washing frequency,^157^ and the unresolved issue of how to handle HairGC outliers.^158^, ^159^ These characteristics were often not reported in the included studies, which prevented comparison across studies. Then again, the results of our analyses in the subgroup of studies that accounted for outliers and corticosteroid use, the two issues that are most likely related to obesity, did not differ significantly from the results in the subgroup of studies that did not account for outliers, corticosteroid use, or neither. It should however be noted that we only assessed whether studies handled outliers at all and that the exact manner of handling outliers in (psycho)endocrine research is still a separate topic of discussion.^159^ Lastly, this review only included cross‐sectional associations while any conclusion on the prognostic or predictive value of HairGC for future obesity should come from studies investigating longitudinal relations, which have however until now only been performed scarcely.^15^, ^134^

Altogether, we confirmed a consistent positive association between anthropometric measurements and hair glucocorticoids. This relation was most often studied for hair cortisol and BMI but showed the strongest correlation and largest effect size for hair cortisone and WC. These relations were not influenced by mean age, mean BMI, or mean HairGC levels nor by the used laboratory methods of the studies. However, the percentage of males, the percentage of participants with obesity, and objective measurement of weight instead of self‐reported weight represented important features to take into account when assessing hair glucocorticoids in cohorts. Although causality is not yet proven, our results suggest that higher long‐term glucocorticoid levels measured in scalp hair, especially cortisone, may contribute to or reflect the state of specifically central adiposity. Future longitudinal studies should investigate whether higher hair glucocorticoid levels can have clinical relevance in predicting the development or deterioration of obesity. Our results emphasize the importance of accounting for BMI and/or WC or WHR when interpreting hair glucocorticoid levels in individuals or on a group level.

## FUNDING INFORMATION

OA, BvdV, EvdA, and EvR are supported by the Elisabeth Foundation, a nonprofit organization supporting academic obesity research. EvR is supported by the Netherlands Organization of Scientific Research NWO, ZonMW Vidi Grant/Award Number: 91716453.

## CONFLICT OF INTEREST

The authors declare that there are no conflicts of interest for all authors.

## AUTHOR CONTRIBUTIONS

EvdV, OA, and MM: conceptualization, data curation, formal analysis, investigation, methodology, validation, visualization, and writing‐original draft. AA: data curation, formal analysis, investigation, visualization, and writing‐review and editing. VW, AI, EvdA, YdR, and BvdV: formal analysis, investigation, methodology, supervision, validation, and writing‐review and editing. TS: data curation, formal analysis, investigation, supervision, validation, and writing‐review and editing. SH: conceptualization, formal analysis, investigation, methodology, supervision, validation, visualization, writing‐review and editing. EvR: conceptualization, formal analysis, funding acquisition, investigation, methodology, project administration, resources, supervision, validation, visualization, writing‐review and editing.

## Supporting information


**Appendix S1.** Search strategy.
**Table S1.** Qualitative synthesis.
**Figure S1.** Forest plot for the meta‐analysis of correlation coefficients between HairF and BMI.
**Figure S2.** Forest plot for the meta‐analysis of correlation coefficients between HairF and BMI SDS.
**Figure S3.** Forest plot for the meta‐analysis of correlation coefficients between HairF and WC.
**Figure S4.** Forest plot for the meta‐analysis of correlation coefficients between HairF and WHR.
**Figure S5.** Forest plot for the meta‐analysis of correlation coefficients between HairE and BMI.
**Figure S6.** Forest plot for the meta‐analysis of correlation coefficients between HairE and WC.
**Figure S7.** Bubble plot for the meta‐regression on proportion of males in the meta‐analysis of correlation coefficients between HairF and WC.
**Figure S8.** Bubble plot for the meta‐regression on proportion of males in the meta‐analysis of correlations between HairF and WHR.
**Figure S9.** Bubble plot for the meta‐regression on proportion of individuals with obesity in the meta‐analysis of correlations between HairF and BMI.
**Figure S10.** Funnel plot for the meta‐analysis of correlation coefficients between HairF and BMI.
**Figure S11.** Funnel plot for the meta‐analysis of correlation coefficients between HairF and BMI SDS.
**Figure S12.** Funnel plot for the meta‐analysis of correlation coefficients between HairF and WC.
**Figure S13.** Funnel plot for the meta‐analysis of correlation coefficients between HairF and WHR.
**Figure S14.** Funnel plot for the meta‐analysis of correlation coefficients between HairE and BMI.
**Figure S15.** Funnel plot for the meta‐analysis of correlation coefficients between HairE and WC.
