# Supplementary material for: Cross‐sectional relation of long‐term glucocorticoids in hair with anthropometric measurements and their possible determinants: A systematic review and meta‐analysis
Source: Obes Rev. 2021 Nov 22;23(3):e13376. doi: 10.1111/obr.13376 (PMC9285618; doi:10.1111/obr.13376)
Supplement: Supplementary file 1 — Appendix S1. Search strategy. Table S1. Qualitative synthesis. Figure S1. Forest plot for the meta‐analysis of correlation coefficients between HairF and BMI. Figure S2. Forest plot for the meta‐analysis of correlation coefficients between HairF and BMI SDS. Figure S3. Forest plot for the meta‐analysis of correlation coefficients between HairF and WC. Figure S4. Forest plot for the meta‐analysis of correlation coefficients between HairF and WHR. Figure S5. Forest plot for the meta‐analysis of correlation coefficients between HairE and BMI. Figure S6. Forest plot for the meta‐analysis of correlation coefficients between HairE and WC. Figure S7. Bubble plot for the meta‐regression on proportion of males in the meta‐analysis of correlation coefficients between HairF and WC. Figure S8. Bubble plot for the meta‐regression on proportion of males in the meta‐analysis of correlations between HairF and WHR. Figure S9. Bubble plot for the meta‐regression on proportion of individuals with obesity in the meta‐analysis of correlations between HairF and BMI. Figure S10. Funnel plot for the meta‐analysis of correlation coefficients between HairF and BMI. Figure S11. Funnel plot for the meta‐analysis of correlation coefficients between HairF and BMI SDS. Figure S12. Funnel plot for the meta‐analysis of correlation coefficients between HairF and WC. Figure S13. Funnel plot for the meta‐analysis of correlation coefficients between HairF and WHR. Figure S14. Funnel plot for the meta‐analysis of correlation coefficients between HairE and BMI. Figure S15. Funnel plot for the meta‐analysis of correlation coefficients between HairE and WC. [file OBR-23-0-s001.zip › obr13376-sup-0001-supporting information.pdf]

Supplementary appendix to: "Cross-sectional relation of long-term glucocorticoids in hair with anthropometric measurements and their possible determinants: a systematic review and meta-analysis"

E.S. van der Valk[1,2*]; O. Abawi[2,3*]; M. Mohseni[1,2*]; A. Abdelmoumen[1,2]; V.L. Wester[1,2]; B. van der Voorn[1,2,3], A.M. Iyer[1,2]; E.L.T. van den Akker[2,3]; S.E. Hoeks[4]; S.A.A. van den Berg[1,5]; Y.B. de Rijke[5]; T. Stalder[6]; E.F.C. van Rossum[1,2]

1. Department of Internal Medicine, Erasmus MC, University Medical Center Rotterdam, Rotterdam, The Netherlands
2. Obesity Center CGG, Erasmus MC, University Medical Center Rotterdam, Rotterdam, The Netherlands
3. Department of Pediatrics, division of Endocrinology, Erasmus MC-Sophia Children's Hospital, University Medical Center Rotterdam, Rotterdam, The Netherlands
4. Department of Anesthesiology, Erasmus University Medical Center, Rotterdam, Netherlands.
5. Department of Clinical Chemistry, Erasmus MC, University Medical Center Rotterdam, Rotterdam, The Netherlands
6. Department of Clinical Psychology, University of Siegen, Siegen, Germany

* shared first authorship

Corresponding Author: Elisabeth F.C. van Rossum, MD PhD, internist-endocrinologist/professor of Medicine, Department of Internal Medicine and Obesity Center CGG (Centrum Gezond Gewicht), Erasmus MC, University Medical Center Rotterdam, Rotterdam, The Netherlands, Room Rg-5. P.O. Box 2400, 3000 CA Rotterdam, The Netherlands. Email: e.vanrossum@erasmusmc.nl

Table of contents

Appendix 1. Search strategy.

Supplementary Table S1. Qualitative synthesis

Supplemental Figure S1. Forest plot for the meta-analysis of correlation coefficients between **HairF and BMI**.
Supplemental Figure S2. Forest plot for the meta-analysis of correlation coefficients between **HairF and BMI SDS**.
Supplemental Figure S3. Forest plot for the meta-analysis of correlation coefficients between **HairF and WC**.
Supplemental Figure S4. Forest plot for the meta-analysis of correlation coefficients between **HairF and WHR**.
Supplemental Figure S5. Forest plot for the meta-analysis of correlation coefficients between **HairE and BMI**.
Supplemental Figure S6. Forest plot for the meta-analysis of correlation coefficients between **HairE and WC**.
Supplemental Figure S7. Bubble plot for the meta-regression on proportion of males in the meta-analysis of correlation coefficients between **HairF and WC**.

Supplemental Figure S8. Bubble plot for the meta-regression on proportion of males in the meta-analysis of correlations between **HairF and WHR**.
Supplemental Figure S9. Bubble plot for the meta-regression on proportion of individuals with obesity in the meta-analysis of correlations between **HairF and BMI**.
Supplemental Figure S10. Funnel plot for the meta-analysis of correlation coefficients between **HairF and BMI**.
Supplemental Figure S11. Funnel plot for the meta-analysis of correlation coefficients between **HairF and BMI SDS**.
Supplemental Figure S12. Funnel plot for the meta-analysis of correlation coefficients between **HairF and WC**.
Supplemental Figure S13. Funnel plot for the meta-analysis of correlation coefficients between **HairF and WHR**.
Supplemental Figure S14. Funnel plot for the meta-analysis of correlation coefficients between **HairE and BMI**.
Supplemental Figure S15. Funnel plot for the meta-analysis of correlation coefficients between **HairE and WC**.

Supplementary appendix references

Appendix 1. Search strategy.

Search date: 16 November 2020

Embase

('hydrocortisone'/exp OR (cortisol* OR cortison* OR hypocortisol* OR hypercortisol*):ab,ti,kw) AND (hair/de OR 'scalp hair'/de OR 'hair level'/exp OR 'hair analysis'/exp OR (hair OR hairs):ab,ti,kw) AND ('body mass'/exp OR 'waist circumference'/de OR 'waist hip ratio'/exp OR 'body weight'/exp OR obesity/exp OR 'anthropometric parameters'/de OR anthropometry/de OR 'birth weight'/exp OR weight/de OR 'cardiometabolic risk'/exp OR 'skinfold thickness'/de OR 'body fat'/de OR 'health status'/de OR 'general health status assessment'/exp OR ((body NEAR/3 mass*) OR weight OR 'birth weight' OR birthweight OR bmi OR (waist NEAR/3 (circumferen* OR hip)) OR obes* OR (metabol* NEAR/3 syndrom*) OR overweight* OR anthropometr* OR (physiological* NEAR/3 measure*) OR (cardiometabol* NEAR/3 risk) OR 'body fat' OR (fat NEAR/3 percentage*) OR ((health OR functional*) NEAR/3 (measure* OR status* OR state OR general*))):ab,ti,kw) NOT ([animals]/lim NOT [humans]/lim)

Medline Ovid

("hydrocortisone"/ OR (cortisol* OR cortison* OR hypocortisol* OR hypercortisol*).ab,ti,kf.) AND (hair/ OR (hair OR hairs).ab,ti,kf.) AND ("Body Weights and Measures"/ OR "Body Mass Index"/ OR exp "Body Weight"/ OR exp "waist circumference"/ OR "Waist-Hip Ratio"/ OR "Skinfold Thickness"/ OR "body weight"/ OR obesity/ OR Anthropometry/ OR exp "birth weight"/ OR exp "Health Status"/ OR ((body ADJ3 mass*) OR weight OR "birth weight" OR birthweight OR bmi OR (waist ADJ3 (circumferen* OR hip)) OR obes* OR (metabol* ADJ3 syndrom*) OR overweight* OR anthropometr* OR (physiological* ADJ3 measure*) OR (cardiometabol* ADJ3 risk) OR "body fat" OR (fat ADJ3 percentage*) OR ((health OR functional*) ADJ3 (measure* OR status* OR state OR general*))).ab,ti,kf.) NOT (exp animals/ NOT humans/)

Cochrane

((cortisol* OR cortison* OR hypocortisol* OR hypercortisol*):ab,ti) AND ((hair OR hairs):ab,ti) AND ((body NEAR/3 mass*) OR weight OR 'birth weight' OR birthweight OR bmi OR (waist NEAR/3 (circumferen* OR hip)) OR obes* OR (metabol* NEAR/3 syndrom*) OR overweight* OR anthropometr* OR (physiological* NEAR/3 measure*) OR (cardiometabol* NEAR/3 risk) OR 'body fat' OR (fat NEAR/3 percentage*) OR ((health OR functional*) NEAR/3 (measure* OR status* OR state OR general*))):ab,ti

Web of science

TS=(((cortisol* OR cortison* OR hypocortisol* OR hypercortisol*)) AND ((hair OR hairs)) AND ((((body NEAR/2 (mass*)) OR weight OR "birth weight" OR birthweight OR bmi OR (waist NEAR/2 (circumferen* OR hip)) OR obes* OR (metabol* NEAR/2 syndrom*) OR overweight* OR anthropometr* OR (physiological* NEAR/2 measure*) OR (cardiometabol* NEAR/2 risk)) OR "body fat" OR (fat NEAR/2 percentage*)) OR ((health OR functional*) NEAR/2 (measure* OR status* OR state OR general*))))

Scopus

TITLE-ABS-KEY(((cortisol* OR cortison* OR hypocortisol* OR hypercortisol*)) AND ((hair OR hairs)) AND ((((body W/2 (mass*)) OR weight OR "birth weight" OR birthweight OR bmi OR (waist W/2 (circumferen* OR hip)) OR obes* OR (metabol* W/2 syndrom*) OR overweight* OR anthropometr* OR (physiological* W/2 measure*) OR (cardiometabol* W/2 risk)) OR "body fat" OR (fat W/2 percentage*)) OR ((health OR functional*) W/2 (measure* OR status* OR state OR general*))))

Google scholar

First 100:

Cortisol hair|hairs "body mass |weight"|"birth weight"|birthweight|bmi|"waist circumferen|hip"|obesity|obese|"metabolic syndrome"|overweight|anthropometric|anthropometry|"body fat"|"fat percentage"|"health status"

allintitle: 21

Cortisol hair|hairs "body mass |weight"|"birth weight"|birthweight|bmi|"waist circumferen|hip"|obesity|obese|"metabolic syndrome"|overweight|anthropometric|anthropometry|"body fat"|"fat percentage"|"health status"

Cinahl

(MH "hydrocortisone" OR TI(cortisol* OR cortison* OR hypocortisol* OR hypercortisol*) OR AB(cortisol* OR cortison* OR hypocortisol* OR hypercortisol*)) AND (MH "Hair+" OR TI(hair OR hairs) OR AB(hair OR hairs)) AND (MH "Body Weights and Measures" OR MH "Body Mass Index" OR MH "Body Weight+" OR MH "waist circumference+" OR MH "Waist-Hip Ratio" OR MH "Skinfold Thickness" OR MH "obesity" OR MH "Anthropometry" OR MH "birth weight+" OR MH "Health Status+" OR TI((body N2 mass*) OR weight OR "birth weight" OR birthweight OR bmi OR (waist N2 (circumferen* OR hip)) OR obes* OR (metabol* N2 syndrom*) OR overweight* OR anthropometr* OR (physiological* N2 measure*) OR (cardiometabol* N2 risk) OR "body fat" OR (fat N2 percentage*) OR ((health OR functional*) N2 (measure* OR status* OR state OR general*))) OR AB((body N2 mass*) OR weight OR "birth weight" OR birthweight OR bmi OR (waist N2 (circumferen* OR hip)) OR obes* OR (metabol* N2 syndrom*) OR overweight* OR anthropometr* OR (physiological* N2 measure*) OR (cardiometabol* N2 risk) OR "body fat" OR (fat N2 percentage*) OR ((health OR functional*) N2 (measure* OR status* OR state OR general*)))) NOT (MH "animals+" NOT MH "human")

PsycInfo

(exp hydrocortisone/ OR (cortisol* OR cortison* OR hypocortisol* OR hypercortisol*).ab,ti.) AND (hair/ OR (hair OR hairs).ab,ti.) AND (exp Body Weight/ OR **Body Mass Index**/ OR Obesity/ OR Anthropometry/ OR Health Status/ OR ((body ADJ3 mass*) OR weight OR "birth weight" OR birthweight OR bmi OR (waist ADJ3 (circumferen* OR hip)) OR obes* OR (metabol* ADJ3 syndrom*) OR overweight* OR anthropometr* OR (physiological* ADJ3 measure*) OR (cardiometabol* ADJ3

risk) OR "body fat" OR (fat ADJ3 percentage*) OR ((health OR functional*) ADJ3 (measure* OR status* OR state OR general*))).ab,ti.) NOT (exp animals/ NOT humans/)

Supplementary Table S1. Qualitative overview of reported associations between obesity measurements and HairGC

	BMI	WC	WHR	BMI SDS
HairF- categorical	n= 13,209. 15/26 cohorts show positive relation, i.e., higher HairF levels in individuals with obesity/overweight (1-15); 3 cohorts negative relation, i.e., lower HairF levels in individuals with obesity/overweight (16-18); 1 cohort shows lower BMI in individuals with high HairF levels(19); 7 cohorts found no relation between HairF and BMI(20-26)	n=2,778 2/4 cohorts show higher HairF in individuals with higher WC levels(9, 10); 1 cohort found higher WC in the high HairF group(22); 1 cohort found no relation between HairF and WC(24)	n=271 0/1 cohorts show relation(13)	n=50 1/1 cohort show higher HairF in children with obesity versus those without obesity(12)
HairF- bivariate correlation	n=27,861 34/129 cohorts show positive relation(1, 4, 7, 8, 10, 11, 13, 27-50), 2 cohorts show negative relation(12, 19), 93 cohorts show no relation(2, 3, 5, 6, 12, 14-18, 20, 22-24, 26, 31, 36, 38, 51-108).	n=11,419 11/27 cohorts show positive relation(1, 10, 28, 34, 40, 43, 62, 107, 109-111); 16 cohorts show no relation(3, 15, 16, 18, 24, 51, 72-74, 81, 85, 92, 101-103, 112)	n=7,357 5/18 cohorts show positive relation(1, 62, 109, 110, 113); 13 cohorts show no relation(13, 16-18, 55, 85, 101, 102, 114, 115)	n=1,247 3/11 cohorts show positive relation(30, 116); 1 cohort shows negative relation(12); 8 cohorts show no relation(12, 19, 45, 71, 78, 82, 117, 118)
HairF- partial correlation	n=2,527 1/1 cohort shows positive relation(9)	n=2,527 0/1 cohorts show relation(9)	NA	NA
HairF independent - simple regression	n=18,953 21/54 cohorts show positive relation(1, 4, 10, 11, 27, 28, 30, 32-34, 36, 37, 39, 41, 43, 45-47, 73, 110); 1 cohort shows negative relation(40); 32 cohorts show no relation(2, 12, 17-20, 24, 51, 54, 58, 61, 70, 71, 75, 76, 78, 81-83, 85-87, 89, 91, 99, 101-103, 105, 107)	n=6,500 7/18 cohorts show positive relation(10, 28, 34, 40, 43, 107, 110); 11 cohorts show no relation(18, 24, 36, 51, 73, 81, 85, 101-103, 112)	n=2,334 1/7 cohorts show relation(110); 6 cohorts show no relation(17, 18, 36, 85, 101, 102)	n=1,734 4/11 cohorts show relation(30, 40, 82, 117); 1 cohort shows negative relation(12); 6 cohorts show no relation(12, 20, 36, 45, 71, 78)
HairF dependent - simple regression	n=2,729 5/20 cohorts show positive relation(29, 30, 33, 39, 45); 15 cohorts show no relation(18, 24, 26, 51, 58, 61, 66, 71, 79, 86, 99, 101-103, 107)	n=715 1/6 cohorts show positive relation(107); 5 cohorts show no relation(24, 51, 101-103)	n=389 0/3 cohorts show relation(20, 101, 102)	n=984 2/5 cohorts show positive relation(25, 30); 3 cohorts show no relation(45, 71, 75)
HairF independent - multiple regression	n=1,109 1/2 cohorts show negative relation(44); 1 cohort shows no relation(6)	NA	NA	n=35 0/1 cohorts show relation(19)
HairF dependent - multiple regression	n=3,803 1/2 cohorts show positive relation(48, 119); 1 cohort shows no relation(120)	n=117 0/1 cohorts show relation(20)	n=141 0/1 cohorts show relation(113)	n=117 0/1 cohorts show relation(20)
HairE- categorical	n=2,769 3/5 cohorts show positive relation between HairE and BMI, i.e., higher hairE in individuals with obesity(4,	n=32 0/1 cohorts show relation(24)	n=271 1/1 cohort shows positive relation(13)	NA

	13, 21); 2 cohorts show no relation between HairE and BMI(20, 24)			
HairE- bivariate correlation	n=8,615<br>5/18 cohorts show positive relation(4, 13, 43, 46, 110); 13 cohorts show no relation(8, 20, 23, 24, 50, 57, 61, 73, 82, 101, 103, 107)	n=3,158<br>4/7 cohorts show positive relation(43, 73, 103, 110); 3 cohorts show no relation(24, 101, 107)	n=1,585<br>3/3 cohorts show positive relation(13, 101, 110)	NA
HairE- partial correlation	NA	NA	NA	NA
HairE independent- simple regression	n=5,327<br>3/10 cohorts show positive relation(4, 43, 110); 7 cohorts show no relation(20, 24, 73, 82, 103, 107)	n=3,102<br>4/6 cohorts show positive relation(43, 73, 103, 110); 2 cohorts show no relation(24, 107)	NA	NA
HairE dependent- simple regression	n=434<br>0/4 cohorts show relation(24, 101, 103, 107)	n=434<br>1/4 cohorts show positive relation(103); 3 cohorts show no relation(24, 101, 107)	NA	NA
HairE independent- multiple regression	NA	NA	NA	n=117<br>0/1 cohorts show relation(20)
HairE dependent- multiple regression	NA	n=117<br>0/1 cohorts show relation(20)	NA	

Abbreviations: CI, confidence interval; HairF, hair cortisol; HairE, hair cortisone; SDS, standard deviation score; WC, waist circumference; WHR, waist-to-hip ratio; NA, not available

Supplemental Figure S1. Forest plot for the meta-analysis of correlation coefficients between **HairF and BMI**.

Author, Year, Subgroup	n total	Weight (%)	Corr. [95% CI]
Abdulateef 2019	65	0.58%	0.11 [-0.13, 0.35]
Abell 2016	3634	2.31%	0.14 [0.11, 0.17]
Aguilo 2018	53	0.49%	0.27 [0.01, 0.51]
Berger 2019 - Cohort WPHC	207	1.24%	0.09 [-0.04, 0.23]
Berger 2019 - Cohort YPC	122	0.92%	0.01 [-0.17, 0.18]
Boesch 2014	177	1.14%	0.13 [-0.01, 0.28]
Bossé 2018	598	1.83%	0.12 [0.04, 0.20]
Brianda 2020	134	0.97%	0.26 [0.09, 0.41]
Bryson 2020	297	1.46%	-0.07 [-0.18, 0.04]
Castro-Vale 2020	128	0.95%	0.11 [-0.06, 0.28]
Chan 2014	57	0.52%	0.25 [-0.01, 0.48]
Chen 2013	53	0.49%	0.13 [-0.15, 0.39]
Chen 2015 - female adolescents	47	0.44%	-0.01 [-0.30, 0.27]
Chen 2015 - female adults	75	0.65%	0.21 [-0.02, 0.41]
Chen 2015 - male adolescents	32	0.31%	0.03 [-0.32, 0.37]
Chen 2015 - male adults	10	0.08%	0.32 [-0.39, 0.79]
Davison 2019	344	1.54%	0.01 [-0.10, 0.12]
De Kruijff 2020	278	1.42%	0.28 [0.17, 0.39]
Dettenborn 2010 - employed	28	0.27%	-0.11 [-0.46, 0.28]
Dettenborn 2010 - unemployed	31	0.30%	0.46 [0.13, 0.70]
Diebig 2016	129	0.95%	0.07 [-0.10, 0.24]
Distel 2019	52	0.49%	0.33 [0.06, 0.55]
Dowlati 2010 - controls	87	0.73%	-0.04 [-0.25, 0.17]
Enge 2020	470	1.71%	0.21 [0.12, 0.29]
Engert 2018	332	1.52%	0.17 [0.06, 0.27]
Etwel 2014	39	0.38%	0.05 [-0.27, 0.36]
Evans 2019	92	0.76%	0.01 [-0.20, 0.21]
Feeney 2020	1876	2.20%	0.07 [0.02, 0.11]
Feller 2014	654	1.87%	0.07 [-0.01, 0.15]
Fischer 2017	139	0.99%	0.10 [-0.07, 0.26]
Frisch 2020	18	0.17%	0.29 [-0.20, 0.67]
Gao 2014 - adult control	23	0.22%	0.17 [-0.26, 0.54]
Gao 2014- adult earthquake survivor	20	0.19%	0.11 [-0.35, 0.53]
Gao 2014 - young male control	29	0.28%	0.12 [-0.26, 0.47]
Gao 2014 - young male earthquake survivor	20	0.19%	0.14 [-0.32, 0.55]
Genitsaridi 2019	300	1.46%	-0.05 [-0.17, 0.06]
Gerber 2017	318	1.50%	0.15 [0.04, 0.26]
Gidlow 2016	132	0.96%	0.10 [-0.07, 0.27]
Grass 2015 - study I	42	0.40%	-0.08 [-0.37, 0.23]
Grass 2015 - study II	52	0.49%	0.14 [-0.13, 0.40]
Grunau 2013 - full term	42	0.40%	0.27 [-0.04, 0.53]
Grunau 2013 - pre-term	91	0.75%	0.16 [-0.05, 0.35]
Henley 2014	109	0.85%	-0.06 [-0.24, 0.13]
Hollenbach 2018	59	0.54%	0.33 [0.08, 0.54]
Hu 2017	1263	2.11%	0.21 [0.15, 0.26]
Hunter 2020	140	1.00%	0.09 [-0.08, 0.25]
Ince-Askan 2019	117	0.89%	-0.13 [-0.30, 0.06]
Janssens 2017	111	0.86%	-0.01 [-0.20, 0.17]
Kamps 2014	10	0.08%	0.16 [-0.52, 0.72]
Kozik 2015	66	0.59%	0.20 [-0.04, 0.42]
Kuehl 2015	41	0.39%	0.23 [-0.08, 0.50]
Larsen 2016 - children	363	1.57%	0.05 [-0.05, 0.15]
Larsen 2016 - fathers	231	1.31%	0.17 [0.04, 0.29]
Larsen 2016 - mothers	301	1.47%	0.16 [0.05, 0.27]
Ling 2020 - mothers	35	0.34%	-0.38 [-0.63, -0.05]
Manenschijn 2013	283	1.43%	0.06 [-0.06, 0.18]
Mazgelyte 2019	163	1.09%	0.22 [0.07, 0.36]
McLennan 2016	246	1.34%	0.32 [0.20, 0.43]
Menning 2015 - breast cancer no chemotherapy	33	0.32%	0.50 [0.19, 0.72]
Menning 2015 - controls	38	0.37%	-0.09 [-0.40, 0.24]
Menning 2015- breast cancer chemotherapy	32	0.31%	0.34 [-0.01, 0.62]
Michaud 2016	675	1.88%	0.13 [0.06, 0.20]
Michels 2017	81	0.69%	-0.00 [-0.22, 0.22]
Murray 2016	54	0.50%	-0.26 [-0.49, 0.01]
Nery 2018	16	0.15%	-0.31 [-0.70, 0.22]
Olstad 2016 - women	70	0.62%	-0.03 [-0.26, 0.21]
Ouellet-Morin 2016	34	0.33%	0.26 [-0.09, 0.55]
Ouellette 2015 - high stress daughters	30	0.29%	0.11 [-0.26, 0.45]
Ouellette 2015 - high stress mothers	30	0.29%	0.31 [-0.06, 0.60]
Ouellette 2015 - low stress daughters	30	0.29%	0.09 [-0.28, 0.44]
Ouellette 2015 - low stress mothers	30	0.29%	0.09 [-0.28, 0.44]
Panter-Brick 2019	203	1.23%	0.10 [-0.04, 0.23]
Papafotiou 2017 - normal weight	25	0.24%	0.16 [-0.26, 0.52]
Papafotiou 2017 - obesity	25	0.24%	-0.45 [-0.71, -0.06]
Petimar 2020 - mid-childhood	599	1.83%	0.10 [0.02, 0.18]
Pickett 2020	91	0.75%	0.02 [-0.18, 0.23]
Pittner 2020 - adults	171	1.12%	0.05 [-0.10, 0.20]
Pulopulos 2014	54	0.50%	0.42 [0.17, 0.62]
Pyle Hennessey 2020	100	0.80%	0.07 [-0.13, 0.26]
Qi 2014	39	0.38%	0.24 [-0.08, 0.52]
Radin 2019	166	1.10%	-0.03 [-0.18, 0.12]
Saleem 2013 - completers	56	0.52%	0.12 [-0.15, 0.37]
Saleem 2013 - non-completers	43	0.41%	-0.02 [-0.31, 0.29]
Schalinski 2015 - healthy controls	12	0.11%	-0.10 [-0.64, 0.50]
Schalinski 2019 - healthy controls	75	0.65%	0.01 [-0.22, 0.23]
Schloss 2018	75	0.65%	0.00 [-0.23, 0.23]
Serwinski 2016	164	1.10%	0.12 [-0.03, 0.27]
Skoluda 2012 - controls	70	0.62%	0.19 [-0.05, 0.40]
Skoluda 2012 - endurance athletes	304	1.47%	-0.01 [-0.12, 0.10]
Slopen 2018	344	1.54%	0.02 [-0.09, 0.12]
Smith, J 2019	114	0.88%	0.12 [-0.06, 0.30]
Stalder & Steudte 2012 - study I	155	1.06%	0.33 [0.18, 0.46]
Stalder & Steudte 2012 - study II	58	0.53%	0.28 [0.02, 0.50]
Stalder 2010 - non-alcoholic controls	20	0.19%	0.23 [-0.23, 0.61]
Stalder 2013	1258	2.10%	0.16 [0.11, 0.21]
Stalder 2014 - caregivers	20	0.19%	0.29 [-0.17, 0.65]
Stalder 2014 - controls	20	0.19%	-0.26 [-0.63, 0.20]
Staufenbiel 2015	1425	2.14%	0.13 [0.08, 0.18]
Steudte 2013 - non-traumatized controls	28	0.27%	0.13 [-0.26, 0.48]
Steudte 2013 - traumatized controls	25	0.24%	0.22 [-0.19, 0.57]
Steudte, Kolassa 2011	17	0.16%	0.13 [-0.38, 0.57]
Steudte, Stalder 2011	15	0.14%	0.20 [-0.35, 0.64]
Steudte-Schmiedgen 2015 - non-traumatized soldiers	129	0.95%	0.08 [-0.09, 0.25]
Steudte-Schmiedgen 2017	17	0.16%	0.36 [-0.15, 0.71]
Suijker 2018	15	0.14%	0.46 [-0.06, 0.79]
Van Aken 2018	61	0.55%	0.05 [-0.20, 0.30]
Van Dammen 2020	181	1.16%	0.20 [0.06, 0.34]
Van den Heuvel & Acker 2020	164	1.10%	-0.10 [-0.25, 0.06]
Van den Heuvel & Du Plessis 2020	56	0.52%	-0.01 [-0.27, 0.26]
Van den Heuvel & Stalder 2020	216	1.27%	-0.03 [-0.16, 0.10]
Van der Valk 2020	51	0.48%	0.09 [-0.19, 0.36]
Van Holland 2012	27	0.26%	0.21 [-0.18, 0.55]
Van Manen 2019	32	0.31%	-0.09 [-0.42, 0.27]
Vehmeijer 2020	2042	2.22%	0.12 [0.08, 0.17]
Vepsäläinen 2021	565	1.80%	0.01 [-0.08, 0.09]
Walton 2013	10	0.08%	0.33 [-0.38, 0.79]
Wang 2019	68	0.60%	0.16 [-0.08, 0.39]
Wells 2014	324	1.51%	0.15 [0.04, 0.25]
Wester 2017	295	1.45%	0.09 [-0.03, 0.20]
Wester 2017 - healthy controls	174	1.13%	0.11 [-0.04, 0.26]
Wu 2019	160	1.08%	-0.04 [-0.20, 0.11]
Younge 2015	151	1.04%	0.20 [0.04, 0.34]
Random effects model (Q = 221.36, df = 121, p < 0.0001, I^2 = 51.2%)		100.00%	0.10 [0.08, 0.13]

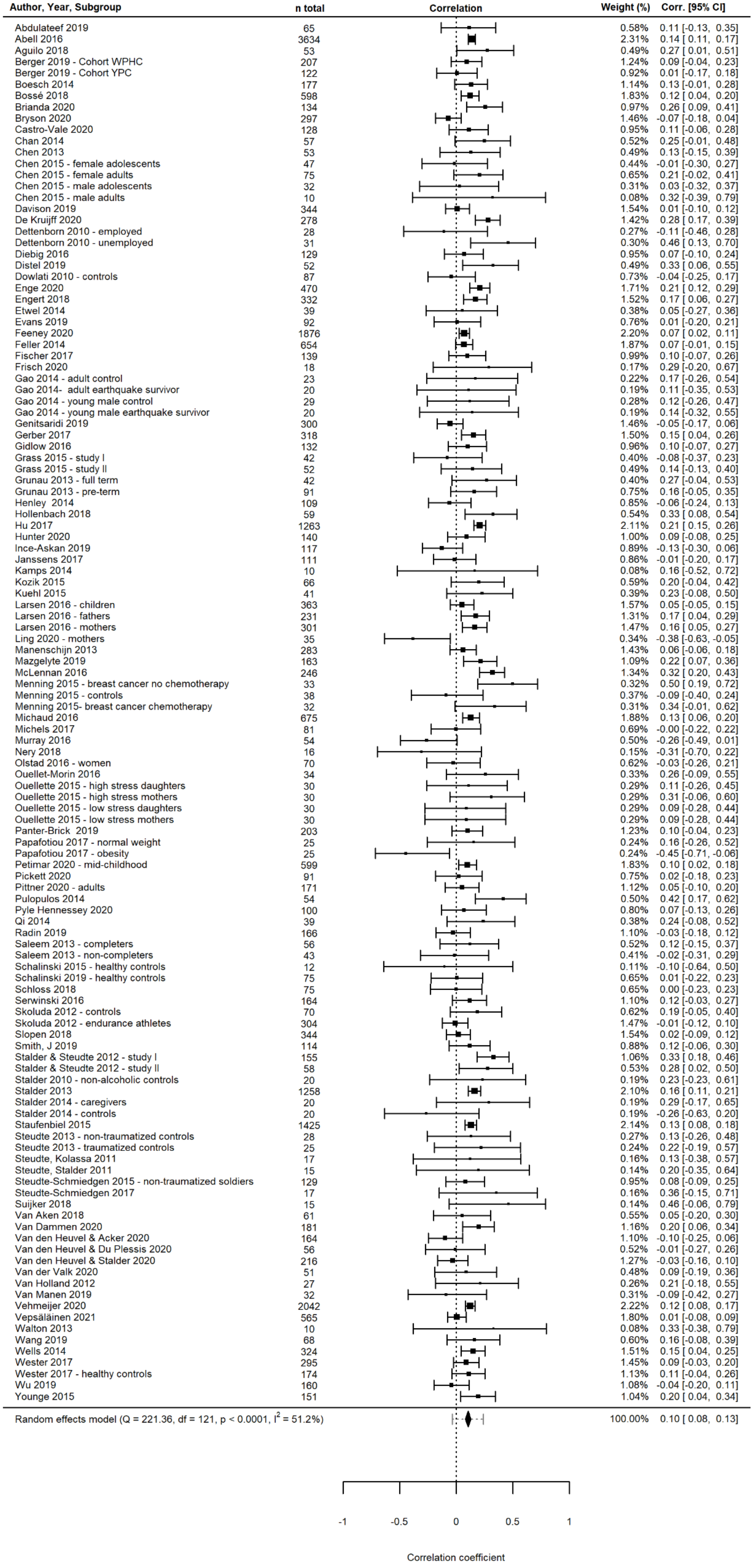

Supplemental Figure S2. Forest plot for the meta-analysis of correlation coefficients between **HairF and BMI SDS**.

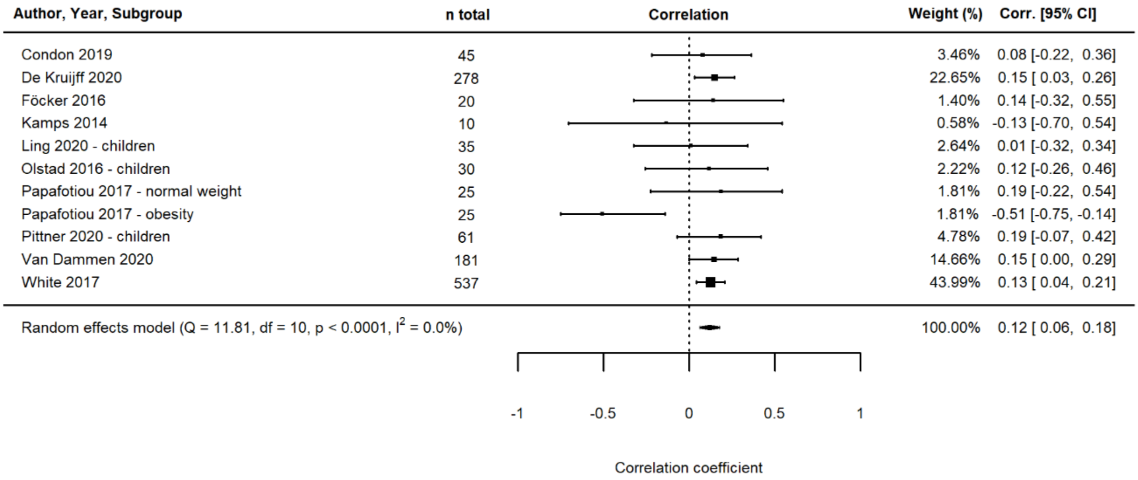

Supplemental Figure S3. Forest plot for the meta-analysis of correlation coefficients between **HairF and WC**.

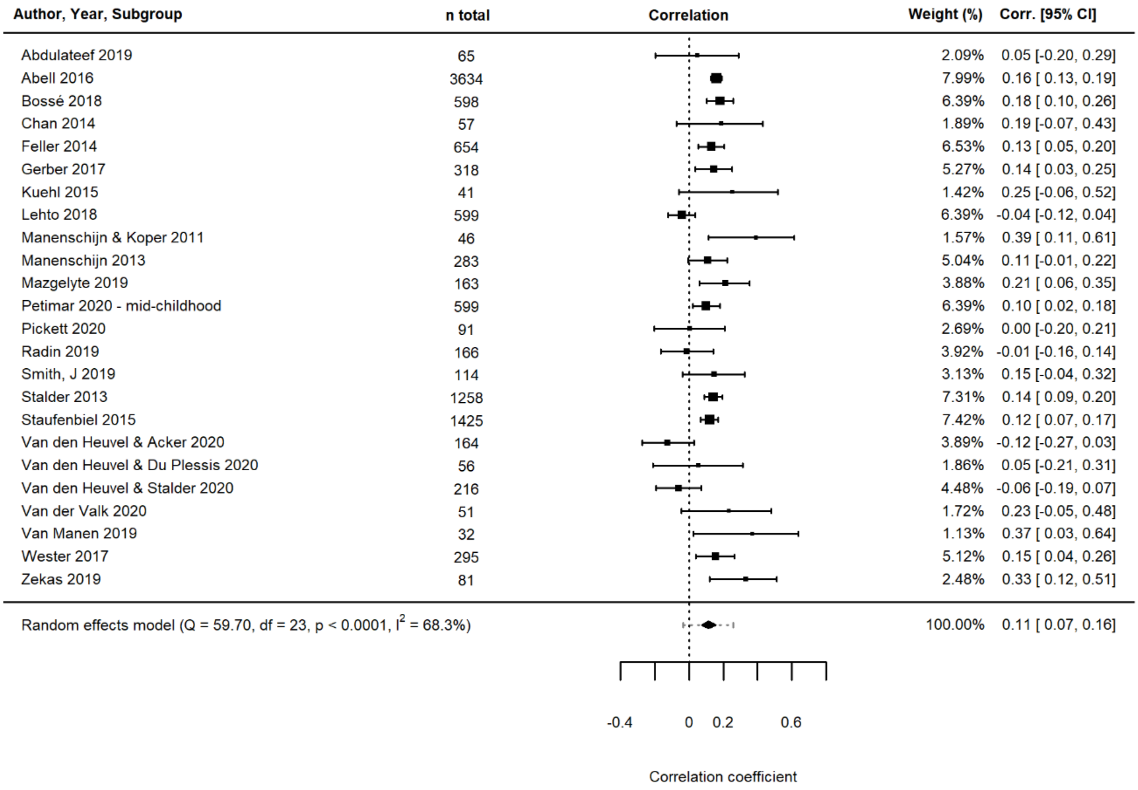

Supplemental Figure S4. Forest plot for the meta-analysis of correlation coefficients between **HairF and WHR**.

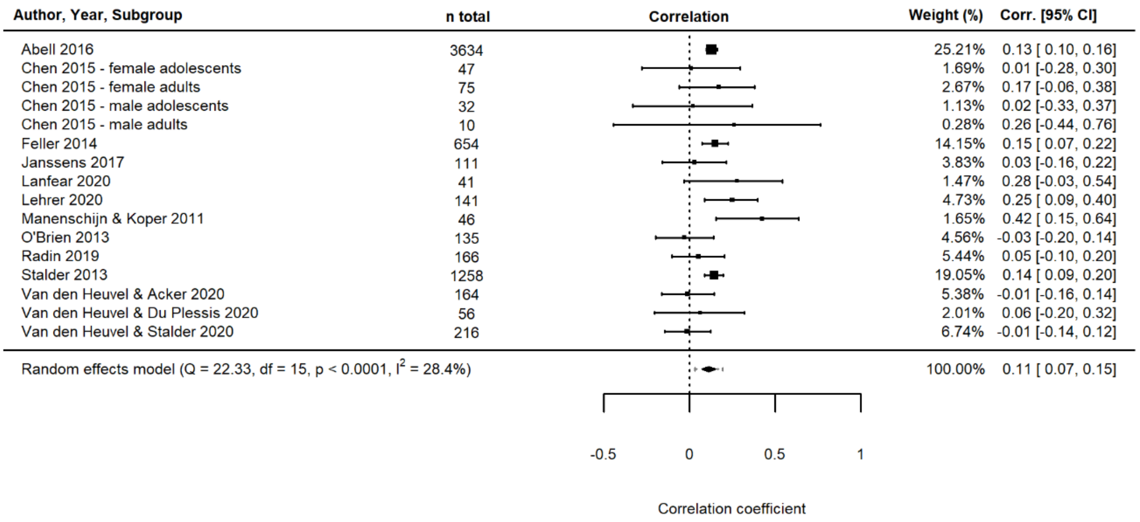

Supplemental Figure S5. Forest plot for the meta-analysis of correlation coefficients between **HairE and BMI**.

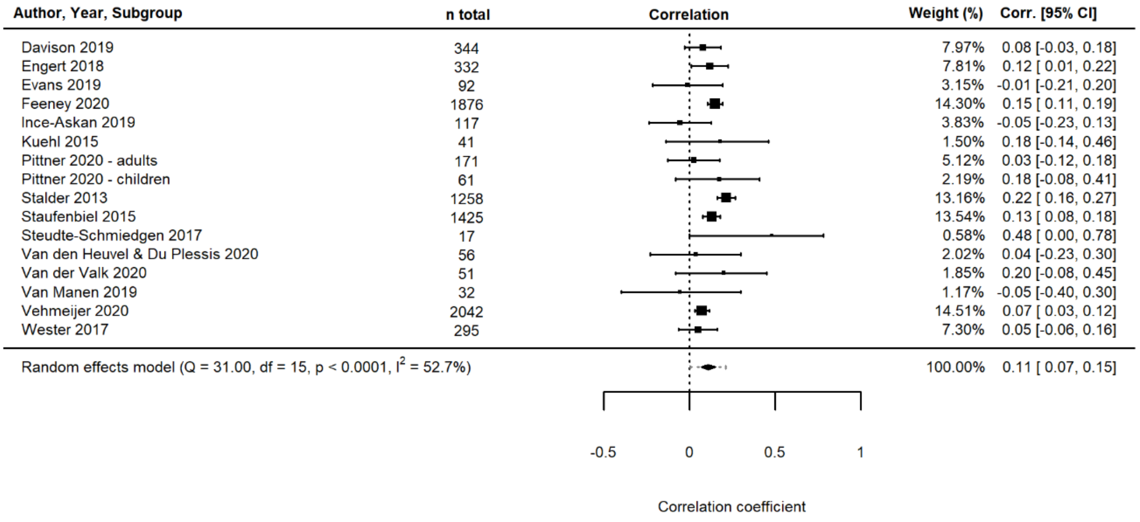

Supplemental Figure S6. Forest plot for the meta-analysis of correlation coefficients between **HairE and WC**.

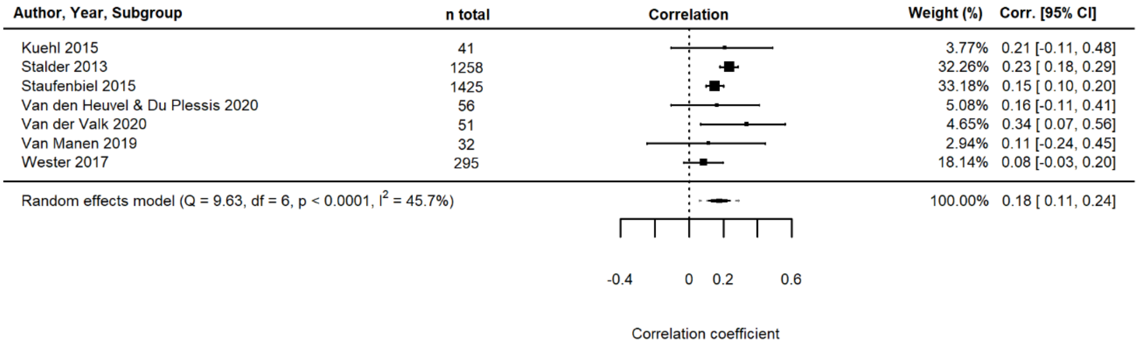

Supplemental Figure S7. Bubble plot for the meta-regression on proportion of males in the meta-analysis of correlation coefficients between **HairF and WC**. The size of the dots represents the study sample size. The dashed lines represent the 95% confidence interval.

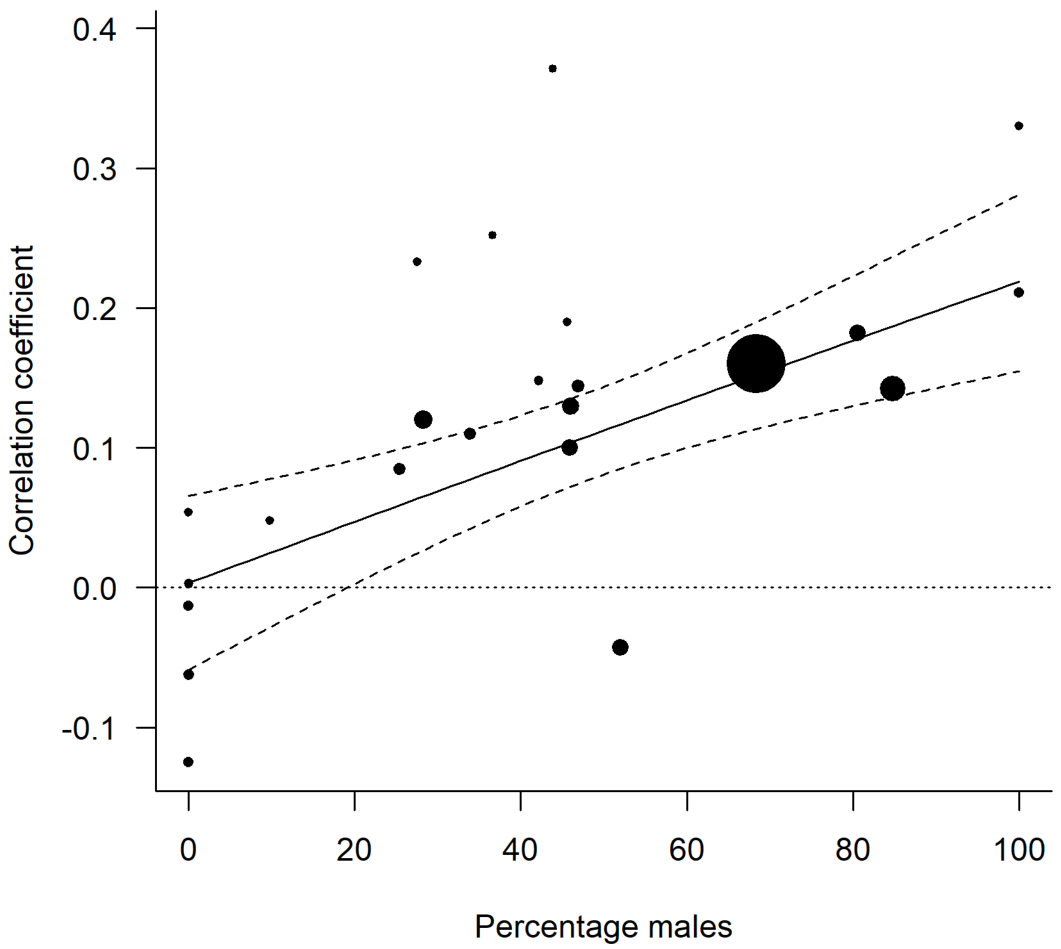

Supplemental Figure S8. Bubble plot for the meta-regression on proportion of males in the meta-analysis of correlations between **HairF and WHR**. The size of the dots represents the study sample size. The dashed lines represent the 95% confidence interval.

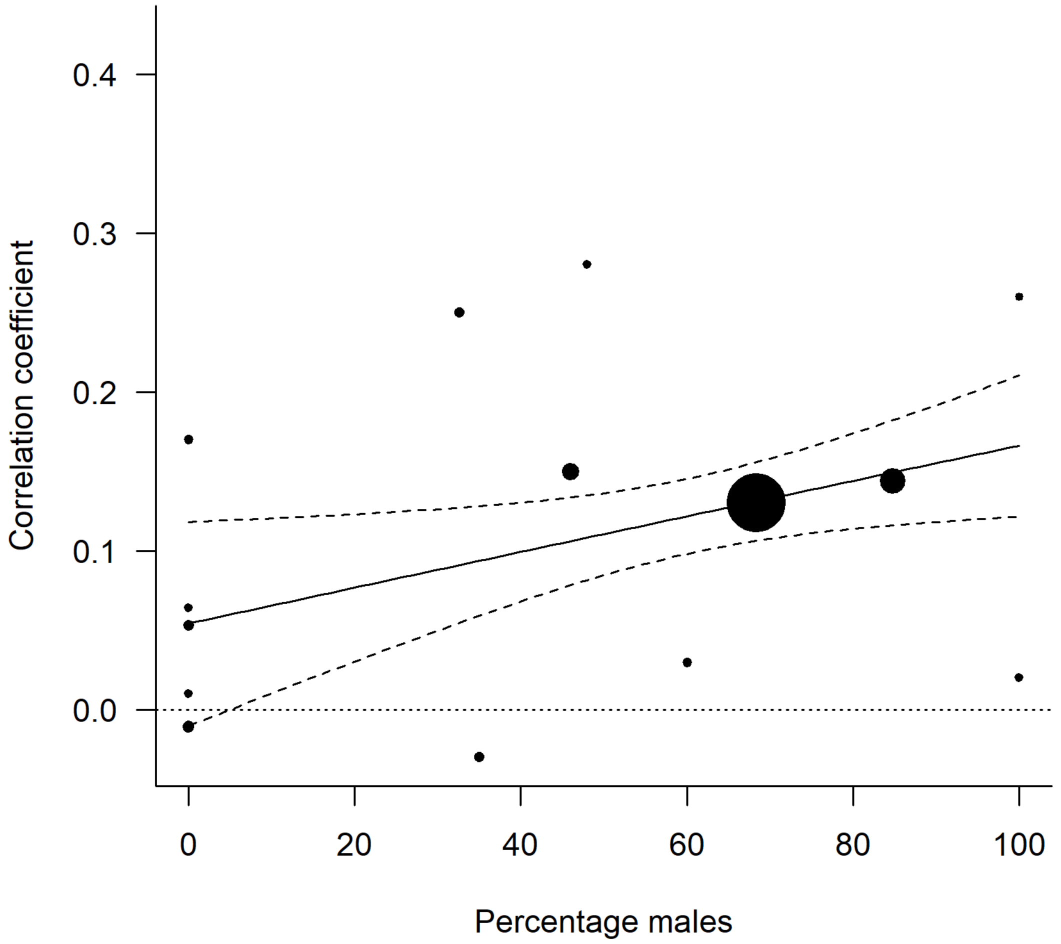

Supplemental Figure S9. Bubble plot for the meta-regression on proportion of individuals with obesity in the meta-analysis of correlations between **HairF and BMI**. The size of the dots represents the study sample size. The dashed lines represent the 95% confidence interval.

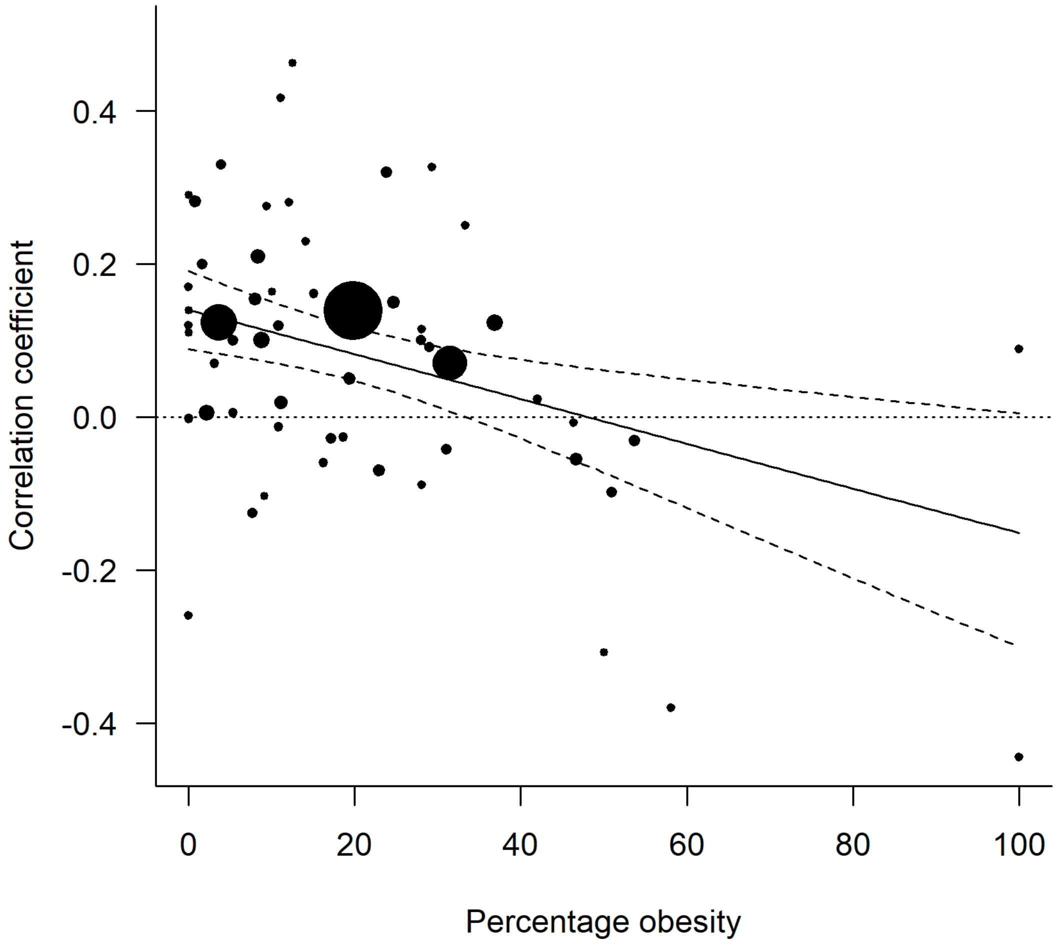

Supplemental Figure S10. Funnel plot for the meta-analysis of correlation coefficients between **HairF and BMI**.

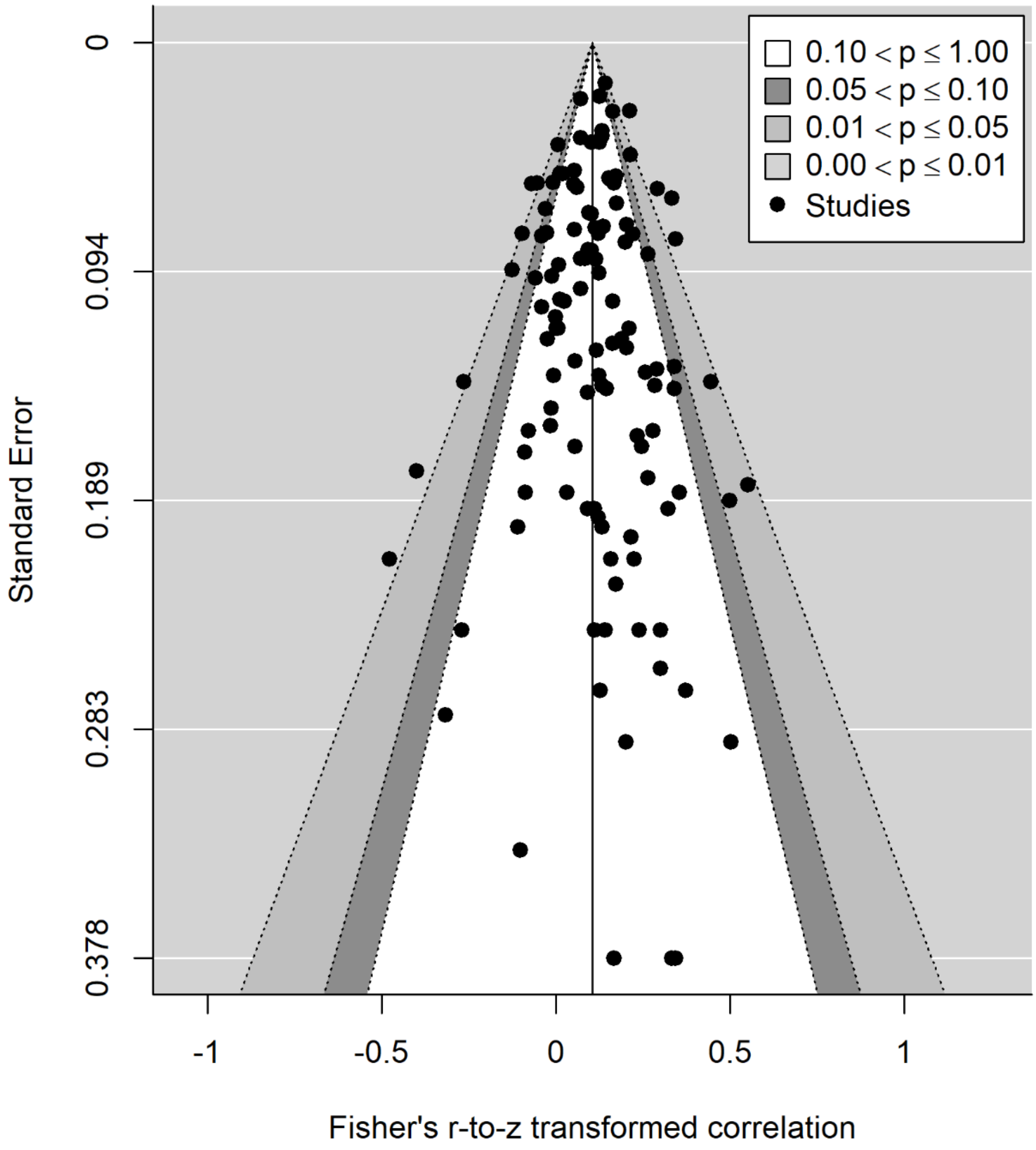

Supplemental Figure S11. Funnel plot for the meta-analysis of correlation coefficients between **HairF and BMI SDS**.

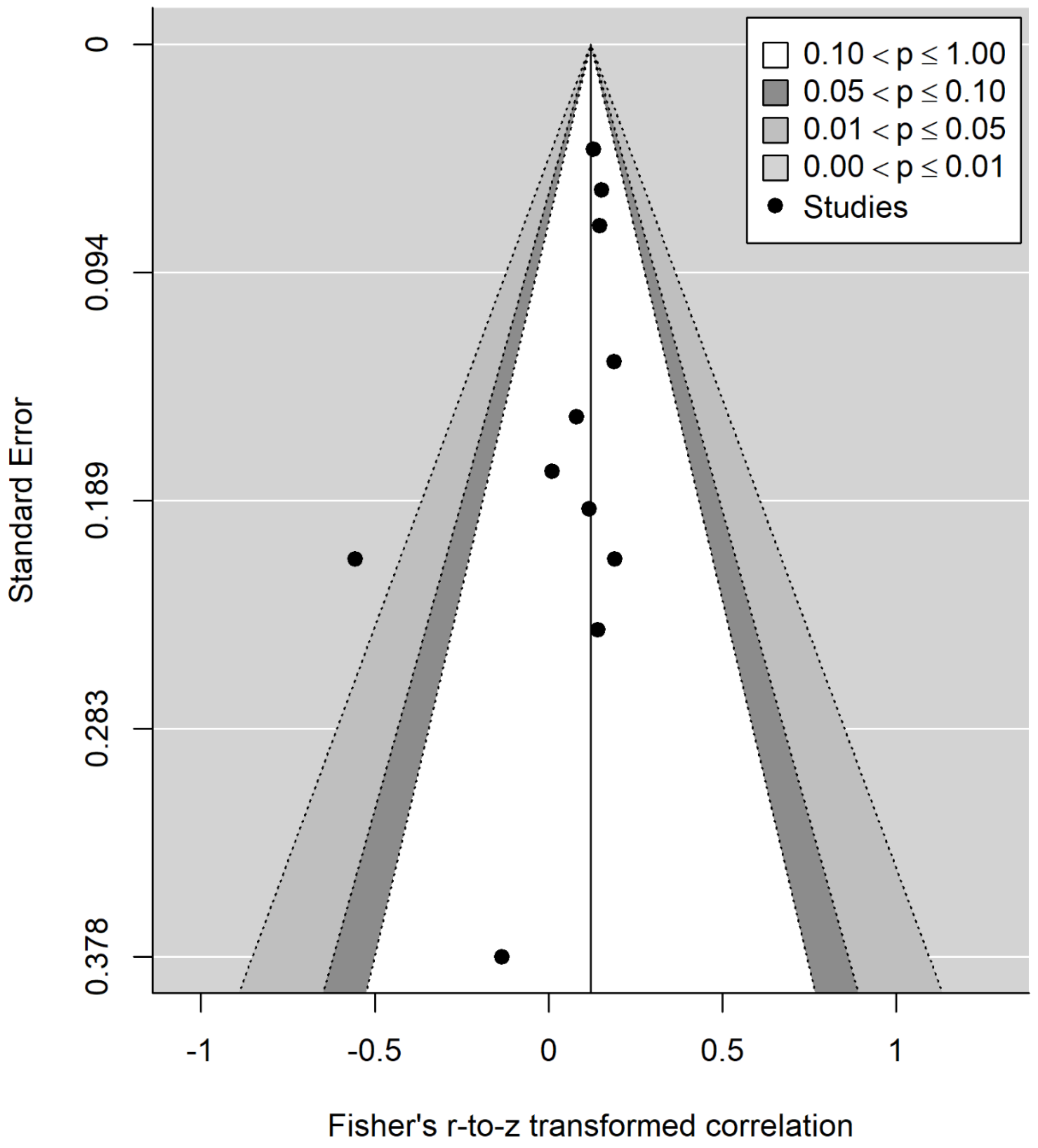

Supplemental Figure S12. Funnel plot for the meta-analysis of correlation coefficients between **HairF and WC**.

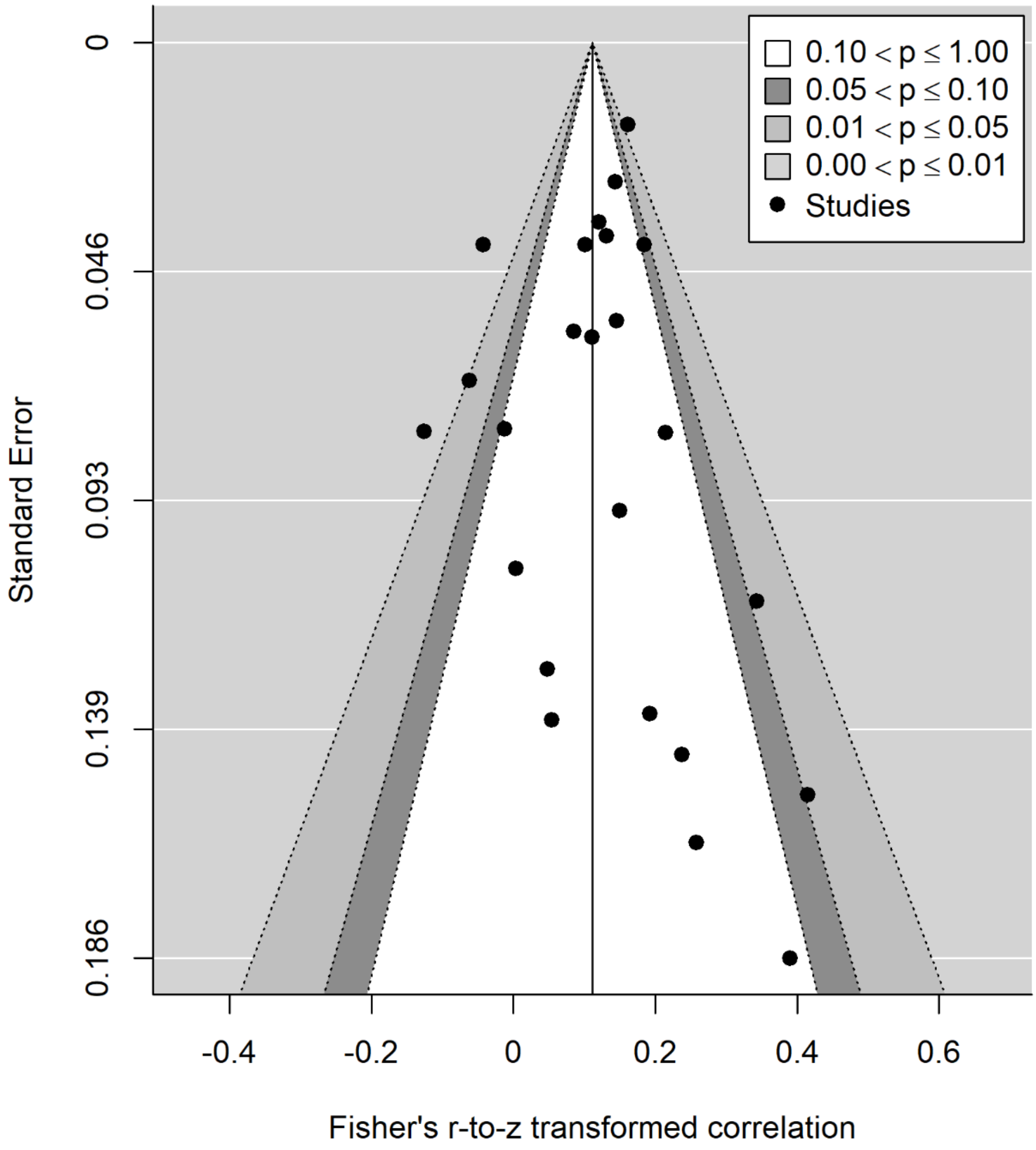

Supplemental Figure S13. Funnel plot for the meta-analysis of correlation coefficients between **HairF and WHR**.

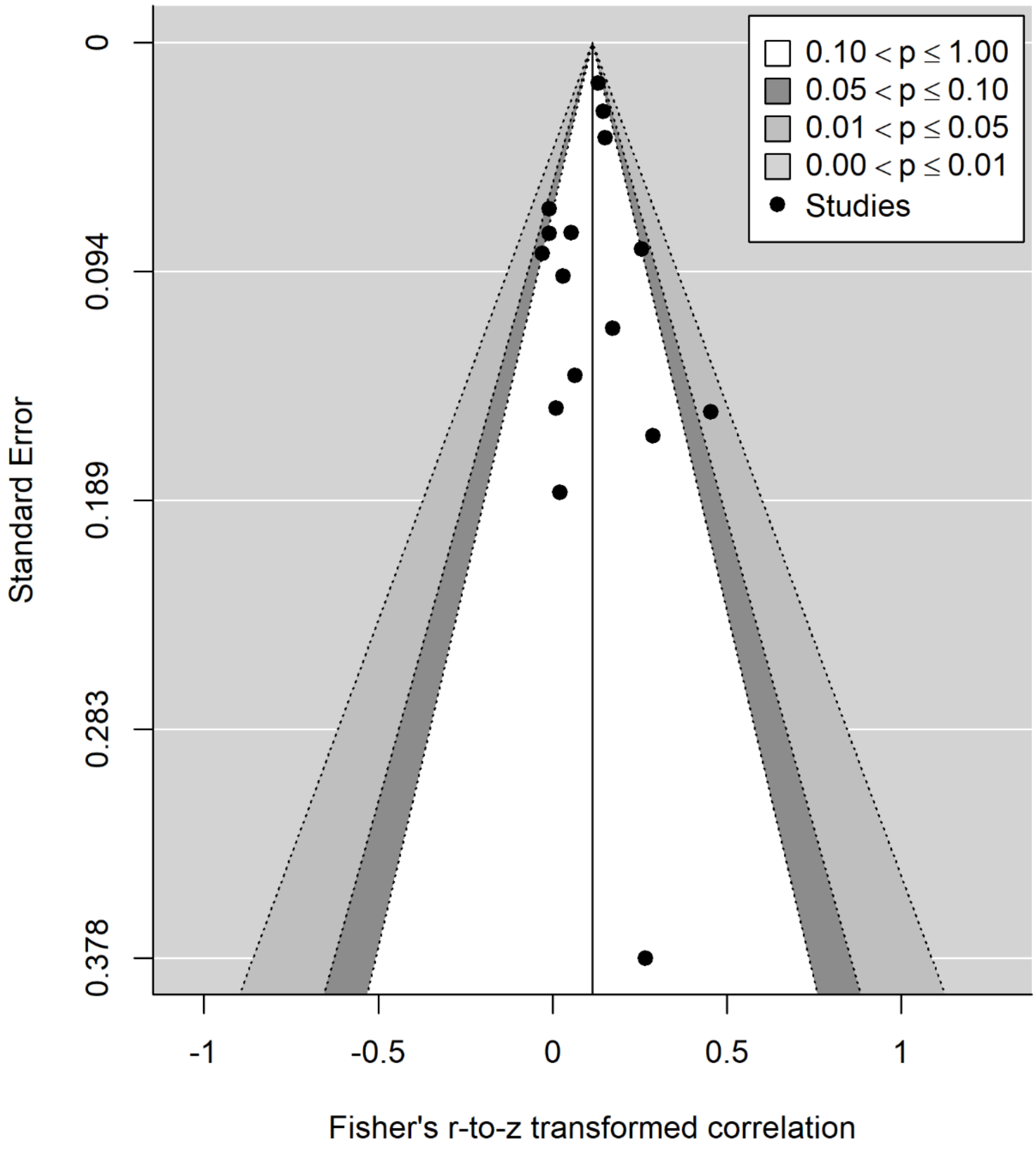

Supplemental Figure S14. Funnel plot for the meta-analysis of correlation coefficients between **HairE and BMI**.

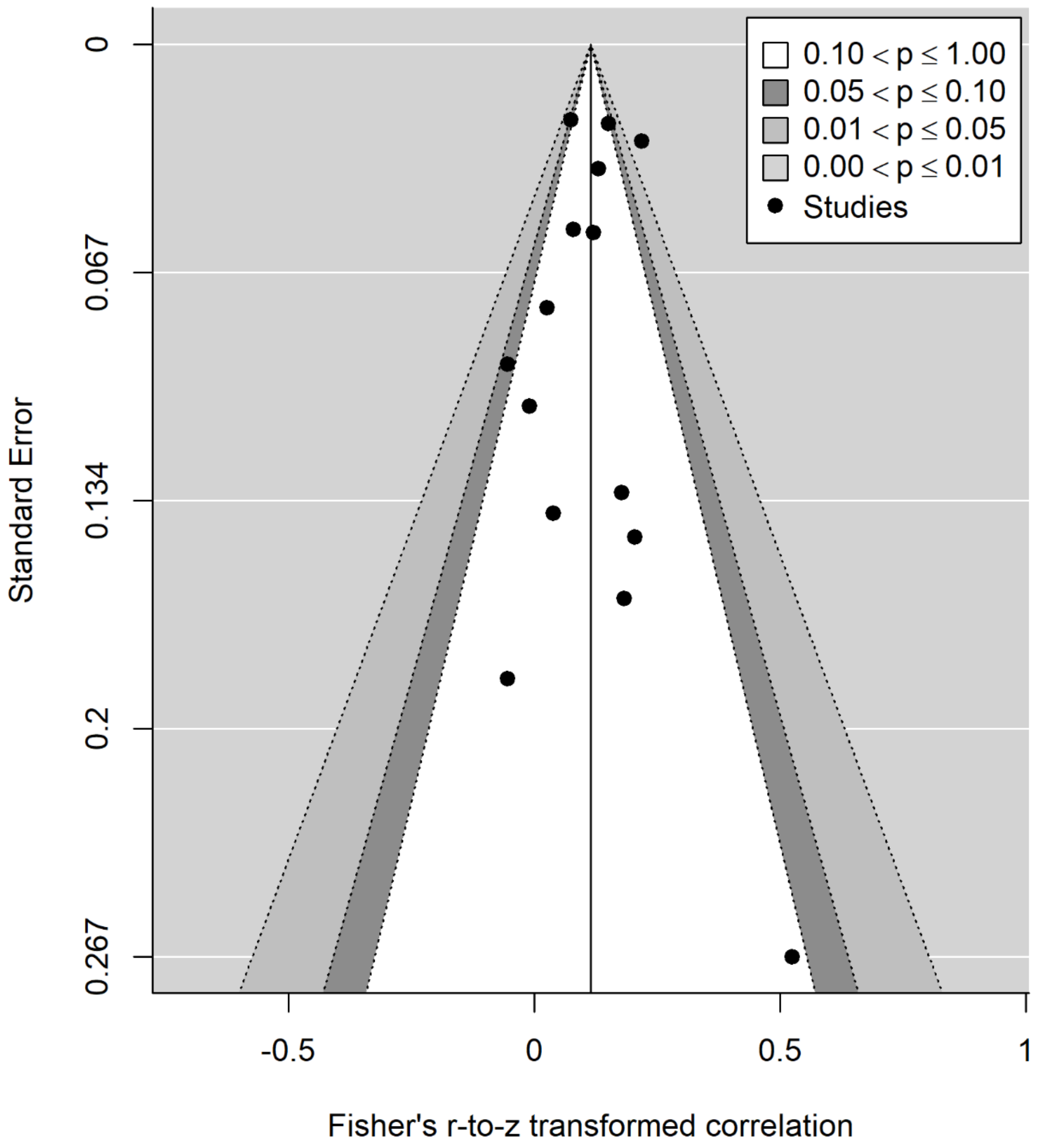

Supplemental Figure S15. Funnel plot for the meta-analysis of correlation coefficients between **HairE and WC**.

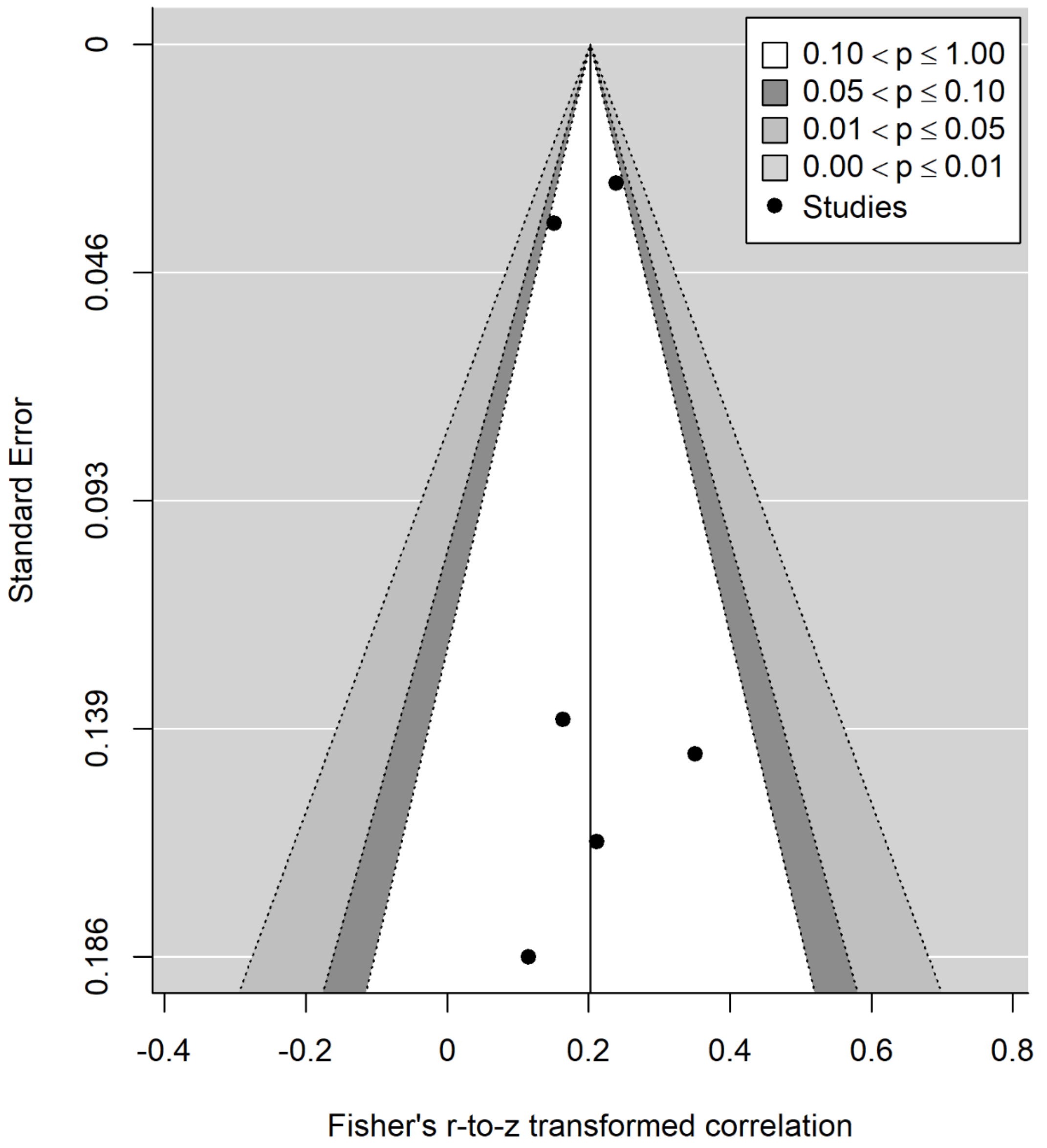

Supplementary appendix references

1. Abell JG, Stalder T, Ferrie JE, Shipley MJ, Kirschbaum C, Kivimäki M, et al. Assessing cortisol from hair samples in a large observational cohort: The Whitehall II study. Psychoneuroendocrinology. 2016;73:148-56.
2. Bryson HE, Mensah F, Goldfeld S, Price AMH. Using Hair Cortisol to Examine the Role of Stress in Children's Health Inequalities at 3 Years. Acad Pediatr. 2019;20(2):193-202.
3. Chan J, Sauvé B, Tokmakejian S, Koren G, Van Uum S. Measurement of cortisol and testosterone in hair of obese and non-obese human subjects. Exp Clin Endocrinol Diabetes. 2014;122(6):356-62.
4. Feeney JC, O'Halloran AM, Kenny RA. The Association Between Hair Cortisol, Hair Cortisone, and Cognitive Function in a Population-Based Cohort of Older Adults: Results From The Irish Longitudinal Study on Ageing. J Gerontol A Biol Sci Med Sci. 2020;75(2):257-65.
5. Fischer S, Duncko R, Hatch SL, Papadopoulos A, Goodwin L, Frissa S, et al. Sociodemographic, lifestyle, and psychosocial determinants of hair cortisol in a South London community sample. Psychoneuroendocrinology. 2017;76:144-53.
6. Henley P, Lowthers M, Koren G, Fedha PT, Russell E, Vanuum S, et al. Cultural and socio-economic conditions as factors contributing to chronic stress in sub-saharan African communities. Can J Physiol Pharmacol. 2014;92(9):725-32.
7. Hu JJ, Duan XN, Fang J, Xu N, Wan YH, Su PY, et al. Association between hair cortisol concentration and overweight and obesity in 6-9 years old childhood. Chung Hua Yu Fang I Hsueh Tsa Chih. 2017;51(12):1065-8.
8. Ilg L, Kirschbaum C, Li SC, Wimberger P, Nitzsche K, Rosenlocher F, et al. No Association of Antenatal Synthetic Glucocorticoid Exposure and Hair Steroid Levels in Children and Adolescents. J Clin Endocrinol Metab. 2020;105(3):E575-E82.
9. Jackson SE, Kirschbaum C, Steptoe A. Hair cortisol and adiposity in a population-based sample of 2,527 men and women aged 54 to 87 years. Obesity. 2017;25(3):539-44.
10. Mazgelytė E, Karčiauskaitė D, Linkevičiūtė A, Mažeikienė A, Burokienė N, Matuzevičienė R, et al. Association of hair cortisol concentration with prevalence of major cardiovascular risk factors and Allostatic load. Med Sci Monit. 2019;25:3573-82.
11. Michaud DS, Feder K, Keith SE, Voicescu SA, Marro L, Than J, et al. Self-reported and measured stress related responses associated with exposure to wind turbine noise. J Acoust Soc Am. 2016;139(3):1467-79.
12. Papafotiou C, Christaki E, van den Akker ELT, Wester VL, Apostolakou F, Papassotiriou I, et al. Hair cortisol concentrations exhibit a positive association with salivary cortisol profiles and are increased in obese prepubertal girls. Stress. 2017;20(2):217-22.
13. Walther A, Ehlert U. Hair, nail or still saliva? Cortisol measurement in peripheral body substrates and its association with sex steroids and body composition in a sample of middle-aged and older men. Psychoneuroendocrinology. 2016;71S:74.
14. Wang C, Dai J, Li J. Mediating effects of hair cortisol on the mutual association of job burnout and insomnia: A retrospective exploratory study. J Psychiatr Res. 2019;117:62-7.
15. Wester VL, Staufenbiel SM, Veldhorst MAB, Visser JA, Manenschijn L, Koper JW, et al. Long-term cortisol levels measured in scalp hair of obese patients. Obesity. 2014;22(9):1956-8.
16. Genitsaridi SM, Karampatsou S, Papageorgiou I, Mantzou A, Papathanasiou C, Kassari P, et al. Hair Cortisol Concentrations in Overweight and Obese Children and Adolescents. Horm Res Paediatr. 2019:1-8.
17. Janssens H, Clays E, Fiers T, Verstraete AG, de Bacquer D, Braeckman L. Hair cortisol in relation to job stress and depressive symptoms. Occup Med (Lond). 2017;67(2):114-20.
18. van den Heuvel LL, Acker D, du Plessis S, Stalder T, Suliman S, Thorne MY, et al. Hair cortisol as a biomarker of stress and resilience in South African mixed ancestry females. Psychoneuroendocrinology. 2020;113:104543.

19. Ling J, Xu D, Robbins LB, Kao TSA. Obesity and Hair Cortisol: Relationships Varied Between Low-Income Preschoolers and Mothers. Matern Child Health J. 2020;24(12):1495-504.
20. Ince-Askan H, van den Akker ELT, de Rijke YB, van Rossum EFC, Hazes JMW, Dolhain R. Associations between antenatal prednisone exposure and long-term cortisol and cortisone concentrations in children born to women with rheumatoid arthritis: results from a nationwide prospective cohort study. RMD Open. 2019;5(1):e000852.
21. Mwanza C, Chen Z, Zhang Q, Chen S, Wang W, Deng H. Simultaneous HPLC-APCI-MS/MS quantification of endogenous cannabinoids and glucocorticoids in hair. J Chromatogr B Anal Technol Biomed Life Sci. 2016;1028:1-10.
22. Saleem M, Herrmann N, Swardfager W, Oh PI, Shammi P, Koren G, et al. Higher cortisol predicts less improvement in verbal memory performance after cardiac rehabilitation in patients with coronary artery disease. Cardiovasc Psychiatry Nerol. 2013;2013:340342.
23. Steudte-Schmiedgen S, Wichmann S, Stalder T, Hilbert K, Muehlhan M, Lueken U, et al. Hair cortisol concentrations and cortisol stress reactivity in generalized anxiety disorder, major depression and their comorbidity. J Psychiatr Res. 2017;84:184-90.
24. Van Manen MJG, Wester VL, Van Rossum EFC, Van Den Toorn LM, Dorst KY, De Rijke YB, et al. Scalp hair cortisol and testosterone levels in patients with sarcoidosis. PLOS ONE. 2019;14(6):e021576.
25. Wagner M, Kratzsch J, Vogel M, Peschel T, Gaudl A, Ceglarek U, et al. Hair Cortisol Concentration in Healthy Children and Adolescents Is Related to Puberty, Age, Gender, and Body Mass Index. Horm Res Paediatr. 2019:1-8.
26. Wu YK, Berry DC, Schwartz TA. Weight stigma and acculturation in relation to hair cortisol among Asian Americans with overweight and obesity: A cross-sectional study. Health Psychol Open. 2019;6(1):2055102919829275.
27. Aguiló S, García E, Arza A, Garzón-Rey JM, Aguiló J. Evaluation of chronic stress indicators in geriatric and oncologic caregivers: a cross-sectional study. Stress. 2018;21(1):36-42.
28. Bossé S, Stalder T, D'Antono B. Childhood Trauma, Perceived Stress, and Hair Cortisol in Adults with and Without Cardiovascular Disease. Psychosom Med. 2018;80(4):393-402.
29. Brianda ME, Roskam I, Mikolajczak M. Hair cortisol concentration as a biomarker of parental burnout. Psychoneuroendocrinology. 2020;117.
30. de Kruijff I, Noppe G, Kieviet N, Choenni V, Lambregtse-van den Berg MP, Begijn DGA, et al. LC-MS/MS-based reference intervals for hair cortisol in healthy children. Psychoneuroendocrinology. 2020;112:104539.
31. Dettenborn L, Tietze A, Bruckner F, Kirschbaum C. Higher cortisol content in hair among long-term unemployed individuals compared to controls. Psychoneuroendocrinology. 2010;35(9):1404-9.
32. Distel LML, Egbert AH, Bohnert AM, Santiago CD. Chronic Stress and Food Insecurity: Examining Key Environmental Family Factors Related to Body Mass Index Among Low-Income Mexican-Origin Youth. Fam Community Health. 2019;42(3):213-20.
33. Enge S, Fleischhauer M, Hadj-Abo A, Butt F, Kirschbaum C, Schmidt K, et al. Comparison of hair cortisol concentrations between self- and professionally-collected hair samples and the role of five-factor personality traits as potential moderators. Psychoneuroendocrinology. 2020;122:104859.
34. Gerber M, Endes K, Brand S, Herrmann C, Colledge F, Donath L, et al. In 6- to 8-year-old children, hair cortisol is associated with body mass index and somatic complaints, but not with stress, health-related quality of life, blood pressure, retinal vessel diameters, and cardiorespiratory fitness. Psychoneuroendocrinology. 2017;76:1-10.
35. Hollenbach JP, Gherlone N, Simoneau T, Sylvester F, Cloutier MM. Caregiver's hair cortisol is a potential biomarker of a child's asthma. Am J Respir Crit Care Med. 2018;197:A2016.
36. Larsen SC, Fahrenkrug J, Olsen NJ, Heitmann BL. Association between Hair Cortisol Concentration and Adiposity Measures among Children and Parents from the "Healthy Start" Study. PLOS ONE. 2016;11(9):e0163639.
37. McLennan SN, Ihle A, Steudte-Schmiedgen S. Hair cortisol and cognitive performance in working age adults. Psychoneuroendocrinology. 2016;67:100-3.

38. Menning S, de Ruiter MB, Veltman DJ, Koppelmans V, Kirschbaum C, Boogerd W, et al. Multimodal MRI and cognitive function in patients with breast cancer prior to adjuvant treatment--the role of fatigue. Neuroimage Clin. 2015;7:547-54.
39. Panter-Brick C, Wiley K, Sancilio A, Dajani R, Hadfield K. C-reactive protein, Epstein-Barr virus, and cortisol trajectories in refugee and non-refugee youth: Links with stress, mental health, and cognitive function during a randomized controlled trial. Brain Behav Immun. 2019;87:207-17.
40. Petimar J, Rifas-Shiman SL, Hivert MF, Fleisch AF, Tiemeier H, Oken E. Prenatal and childhood predictors of hair cortisol concentration in mid-childhood and early adolescence. PLOS ONE. 2020;15(2):e0228769.
41. Pulopulos MM, Hidalgo V, Almela M, Puig-Perez S, Villada C, Salvador A. Hair cortisol and cognitive performance in healthy older people. Psychoneuroendocrinology. 2014;44:100-11.
42. Stalder T, Steudte S, Alexander N, Miller R, Gao W, Dettenborn L, et al. Cortisol in hair, body mass index and stress-related measures. Biol Psychol. 2012;90(3):218-23.
43. Staufenbiel SM, Penninx BWJH, de Rijke YB, van den Akker ELT, van Rossum EFC. Determinants of hair cortisol and hair cortisone concentrations in adults. Psychoneuroendocrinology. 2015;60:182-94.
44. Sun Y, Fang J, Wan Y, Hu J, Xu Y, Tao F. Polygenic differential susceptibility to cumulative stress exposure and childhood obesity. Int J Obes. 2018;42(6):1177-84.
45. Van Dammen L, De Rooij SR, Behnsen PM, Huizink AC. Sex-specific associations between person and environment-related childhood adverse events and levels of cortisol and DHEA in adolescence. PLOS ONE. 2020;15(6):e023371.
46. Vehmeijer FOL, Santos S, Gaillard R, de Rijke YB, Voortman T, van den Akker ELT, et al. Associations of hair cortisol concentrations with general and organ fat measures in childhood. J Clin Endocrinol Metab. 2020;106(2):e551-e61.
47. Wells S, Tremblay PF, Flynn A, Russell E, Kennedy J, Rehm J, et al. Associations of hair cortisol concentration with self-reported measures of stress and mental health-related factors in a pooled database of diverse community samples. Stress. 2014;17(4):334-42.
48. Younge JO, Wester VL, van Rossum EF, Gotink RA, Wery MF, Utens EM, et al. Cortisol levels in scalp hair of patients with structural heart disease. Int J Cardiol. 2015;184:71-8.
49. Zai C, George J, Irwin D, Shaikh S, Tampakeras M, Sibony D, et al. Stress response genes and hair cortisol levels in first nation communities. Eur Neuropsychopharmacol. 2017;27:S320.
50. Engert V, Kok BE, Puhlmann LMC, Stalder T, Kirschbaum C, Apostolakou F, et al. Exploring the multidimensional complex systems structure of the stress response and its relation to health and sleep outcomes. Brain Behav Immun. 2018;73:390-402.
51. Abdulateef DS, Mahwi TO. Assessment of hair cortisol in euthyroid, hypothyroid, and subclinical hypothyroid subjects. Endocrine. 2019;63(1):131-9.
52. Berger M, Taylor S, Harriss L, Campbell S, Thompson F, Jones S, et al. Hair cortisol, allostatic load, and depressive symptoms in Australian Aboriginal and Torres Strait Islander people. Stress. 2019;22(3):312-20.
53. Boesch M, Sefidan S, Annen H, Ehlert U, Roos L, Van Uum S, et al. Hair cortisol concentration is unaffected by basic military training, but related to sociodemographic and environmental factors. Stress. 2014;18(1):35-41.
54. Castro-Vale I, van Rossum EFC, Staufenbiel SM, Severo M, Mota-Cardoso R, Carvalho D. Hair cortisol as a marker of intergenerational heritage of war? A study of veterans and their offspring. Psychiatry Investig. 2020;17(10):976-86.
55. Chen X, Gelaye B, Velez JC, Barbosa C, Pepper M, Andrade A, et al. Caregivers' hair cortisol: a possible biomarker of chronic stress is associated with obesity measures among children with disabilities. BMC Pediatr. 2015;15(15):9.
56. Chen Z, Li J, Zhang J, Xing X, Gao W, Lu Z, et al. Simultaneous determination of hair cortisol, cortisone and DHEAS with liquid chromatography-electrospray ionization-tandem mass spectrometry in negative mode. J Chromatogr B Analyt Technol Biomed Life Sci. 2013;929:187-94.

57. Davison B, Singh GR, McFarlane J. Hair cortisol and cortisone as markers of stress in Indigenous and non-Indigenous young adults. Stress. 2019;22(2):210-20.
58. Diebig M, Bormann KC, Rowold J. A double-edged sword: Relationship between full-range leadership behaviors and followers' hair cortisol level. The Leadership Quarterly. 2016;27:684-96.
59. Dowlati Y, Herrmann N, Swardfager W, Thomson S, Oh PI, Van Uum S, et al. Relationship between hair cortisol concentrations and depressive symptoms in patients with coronary artery disease. Neuropsychiatr Dis Treat. 2010;6:393-400.
60. Etwel F, Russell E, Rieder MJ, Van Uum SH, Koren G. Hair cortisol as a biomarker of stress in the 2011 Libyan war. Clin Invest Med. 2014;37(6):E403-E8.
61. Evans BE, Beijers R, Hagquist C, de Weerth C. Childhood urbanicity and hair steroid hormone levels in ten-year-old children. Psychoneuroendocrinology. 2019;102:53-7.
62. Feller S, Vigl M, Bergmann MM, Boeing H, Kirschbaum C, Stalder. Predictors of hair cortisol concentrations in older adults. Psychoneuroendocrinology. 2014;39:132-40.
63. Frisch N, Eichler A, Plank AC, Golub Y, Moll GH, Kratz O. Exploring Reference Values for Hair Cortisol: Hair Weight versus Hair Protein. Ther Drug Monit. 2020;42(9):902-8.
64. Gao W, Zhong P, Xie Q, Wang H, Jin J, Deng H, et al. Temporal features of elevated hair cortisol among earthquake survivors. Psychophysiology. 2014;51(4):319-26.
65. Garcia-Leon MA, Peralta-Ramirez MI, Arco-Garcia L, Romero-Gonzalez B, Caparros-Gonzalez RA, Saez-Sanz N, et al. Hair cortisol concentrations in a Spanish sample of healthy adults. PLOS ONE. 2018;13(9):e0204807.
66. Gidlow CJ, Randall J, Gillman J, Silk S. Hair cortisol and self-reported stress in healthy, working adults. Psychoneuroendocrinology. 2016;63:163-9.
67. Golub Y, Kuitunen-Paul S, Panaseth K, Stonawski V, Frey S, Steigleder R, et al. Salivary and hair cortisol as biomarkers of emotional and behavioral symptoms in 6–9year old children. Physiol Behav. 2019;209:112584.
68. Grass J, Kirschbaum C, Miller R, Gao W, Steudte-Schmiedgen S, Stalder T. Sweat-inducing physiological challenges do not result in acute changes in hair cortisol concentrations. Psychoneuroendocrinology. 2015;53:108-16.
69. Grunau RE, Cepeda IL, Chau CM, Brummelte S, Weinberg J, Lavoie PM, et al. Neonatal pain-related stress and NFKBIA genotype are associated with altered cortisol levels in preterm boys at school age. PLOS ONE. 2013;8(9):e73926.
70. Hunter SK, Hoffman MC, McCarthy L, D'Alessandro A, Wyrwa A, Noonan K, et al. Black American Maternal Prenatal Choline, Offspring Gestational Age at Birth, and Developmental Predisposition to Mental Illness. Schizophr Bull. 2020.
71. Kamps AW, Molenmaker M, Kemperman R, van der Veen BS, Bocca G, Veeger NJ. Children with asthma have significantly lower long-term cortisol levels in their scalp hair than healthy children. Acta Paediatr. 2014;103(9):957-61.
72. Kozik P, Hoppmann CA, Gerstorf D. Future time perspective: Opportunities and limitations are differentially associated with subjective well-being and hair cortisol concentration. Gerontology. 2015;61:166–74.
73. Kuehl LK, Hinkelmann K, Muhtz C, Dettenborn L, Wingenfeld K, Spitzer C, et al. Hair cortisol and cortisol awakening response are associated with criteria of the metabolic syndrome in opposite directions. Psychoneuroendocrinology. 2015;51:365-70.
74. Manenschijn L, Schaap L, Van Schoor NM, Van Der Pas S, Peeters GMEE, Lips P, et al. High long-term cortisol levels, measured in scalp hair, are associated with a history of cardiovascular disease. J Clin Endocrinol Metab. 2013;98(5):2078-83.
75. Michels N, Van De Wiele T, De Henauw S. Chronic Psychosocial Stress and Gut Health in Children: Associations with Calprotectin and Fecal Short-Chain Fatty Acids. Psychosom Med. 2017;79(8):927-35.
76. Murray CR, Simmons JG, Allen NB, Byrne ML, Mundy LK, Seal ML, et al. Associations between dehydroepiandrosterone (DHEA) levels, pituitary volume, and social anxiety in children. Psychoneuroendocrinology. 2016;64:31-9.

77. Nery SF, Paiva SPC, Vieira EL, Barbosa AB, Sant'Anna EM, Casalechi M, et al. Mindfulness-based program for stress reduction in infertile women: Randomized controlled trial. Stress Health. 2018;35(1):49-58.
78. Olstad DL, Ball K, Wright C, Abbott G, Brown E, Turner AI. Hair cortisol levels, perceived stress and body mass index in women and children living in socioeconomically disadvantaged neighborhoods: The READI study. Stress. 2016;19(2):158-67.
79. Ouellet-Morin I, Laurin M, Robitaille MP, Brendgen M, Lupien SJ, Boivin M, et al. Validation of an adapted procedure to collect hair for cortisol determination in adolescents. Psychoneuroendocrinology. 2016;70:58-62.
80. Ouellette SJ, Russell E, Kryski KR, Sheikh HI, Singh SM, Koren G, et al. Hair cortisol concentrations in higher- and lower-stress mother-daughter dyads: A pilot study of associations and moderators. Dev Psychobiol. 2015;57(5):519-34.
81. Pickett S, McCoy TP, Odetola L. The Influence of Chronic Stress and Emotions on Eating Behavior Patterns and Weight among Young African American Women. West J Nurs Res. 2020:193945919897541.
82. Pittner K, Buisman RSM, van den Berg LJM, Compier-de Block L, Tollenaar MS, Bakermans-Kranenburg MJ, et al. Not the Root of the Problem-Hair Cortisol and Cortisone Do Not Mediate the Effect of Child Maltreatment on Body Mass Index. Front Psychiatr. 2020;11:387.
83. Pyle Hennessey EM, Kepinska O, Haft SL, Chan M, Sunshine I, Jones C, et al. Hair cortisol and dehydroepiandrosterone concentrations: Associations with executive function in early childhood. Biol Psychol. 2020;155:107946.
84. Qi X, Zhang J, Liu Y, Ji S, Chen Z, Sluiter JK. Relationship between effort–reward imbalance and hair cortisol concentration in female kindergarten teachers. J Psychosom Res. 2014;76(4):329-32.
85. Radin RM, Mason AE, Laudenslager ML, Epel ES. Maternal caregivers have confluence of altered cortisol, high reward-driven eating, and worse metabolic health. PLOS ONE. 2019;14(5):e0216541.
86. Schalinski I, Elbert T, Steudte-Schmiedgen S, Kirschbaum C. The Cortisol Paradox of Trauma-Related Disorders: Lower Phasic Responses but Higher Tonic Levels of Cortisol Are Associated with Sexual Abuse in Childhood. PLOS ONE. 2015;10(8):e0136921.
87. Schalinski I, Teicher MH, Rockstroh B. Early neglect is a key determinant of adult hair cortisol concentration and is associated with increased vulnerability to trauma in a transdiagnostic sample. Psychoneuroendocrinology. 2019;108:35-42.
88. Schloß S, Ruhl I, Müller V, Becker K, Skoluda N, Nater UM, et al. Low hair cortisol concentration and emerging attention-deficit/hyperactivity symptoms in preschool age. Dev Psychobiol. 2018;60(6):722-9.
89. Serwinski B, Salavecz G, Kirschbaum C, Steptoe A. Associations between hair cortisol concentration, income, income dynamics and status incongruity in healthy middle-aged women. Psychoneuroendocrinology. 2016;67:182-8.
90. Skoluda N, Dettenborn L, Stalder T. Elevated hair cortisol concentrations in endurance athletes. Psychoneuroendocrinology. 2012;37:611-7.
91. Slopen N, Roberts AL, LeWinn KZ, Bush NR, Rovnaghi CR, Tylavsky F, et al. Maternal experiences of trauma and hair cortisol in early childhood in a prospective cohort. Psychoneuroendocrinology. 2018;98:168-76.
92. Smith JD, Johnson KA, Whittle S, Allen NB, Simmons JG. Measurement of cortisol, dehydroepiandrosterone, and testosterone in the hair of children: Preliminary results and promising indications. Dev Psychobiol. 2019;61(6):962-70.
93. Stalder T, Kirschbaum C, Heinze K, Steudte S, Foley P, Tietze A, et al. Use of hair cortisol analysis to detect hypercortisolism during active drinking phases in alcohol-dependent individuals. Biol Psychol. 2010;85(3):357-60.
94. Stalder T, Tietze A, Steudte S, Alexander N, Dettenborn L, Kirschbaum C. Elevated hair cortisol levels in chronically stressed dementia caregivers. Psychoneuroendocrinology. 2014;47:26-30.

95. Steudte S, Kirschbaum C, Gao W, Alexander N, Schönfeld S, Hoyer J, et al. Hair cortisol as a biomarker of traumatization in healthy individuals and posttraumatic stress disorder patients. Biol Psychiatry. 2013;74(9):639-46.
96. Steudte S, Kolassa IT, Stalder T, Pfeiffer A, Kirschbaum C, Elbert T. Increased cortisol concentrations in hair of severely traumatized Ugandan individuals with PTSD. Psychoneuroendocrinology. 2011;36(8):1193-2000.
97. Steudte S, Stalder T, Dettenborn L, Klumbies E, Foley P, Beesdo-Baum K, et al. Decreased hair cortisol concentrations in generalised anxiety disorder. Psychiatry Res. 2011;186(2-3):310-4.
98. Steudte-Schmiedgen S, Stalder T, Schönfeld S, Wittchen HU, Trautmann S, Alexander N, et al. Hair cortisol concentrations and cortisol stress reactivity predict PTSD symptom increase after trauma exposure during military deployment. Psychoneuroendocrinology. 2015;59:123-33.
99. Suijker I, Savas M, van Rossum EFC, Langendonk JG. Hair cortisol is elevated in patients with erythropoietic protoporphyria and correlates with body mass index and quality of life. Br J Dermatol. 2018;178(5):1209-10.
100. van Aken M, Oosterman J, van Rijn T, Ferdek M, Ruigt G, Kozicz T, et al. Hair cortisol and the relationship with chronic pain and quality of life in endometriosis patients. Psychoneuroendocrinology. 2018;89:216-22.
101. van den Heuvel LL, du Plessis S, Stalder T, Acker D, Kirschbaum C, Carr J, et al. Hair glucocorticoid levels in Parkinson's disease. Psychoneuroendocrinology. 2020;117:104704.
102. van den Heuvel LL, Stalder T, du Plessis S, Suliman S, Kirschbaum C, Seedat S. Hair cortisol levels in posttraumatic stress disorder and metabolic syndrome. Stress. 2020:1-36.
103. van der Valk ES, van der Voorn B, Iyer AM, van den Berg SAA, Savas M, de Rijke YB, et al. In adults with obesity, copeptin is linked with BMI but is not associated with long-term exposure to cortisol and cortisone. Eur J Endocrinol. 2020;183(6):669-76.
104. van Holland BJ, Frings-Dresen MH, Sluiter JK. Measuring short-term and long-term physiological stress effects by cortisol reactivity in saliva and hair. Int Arch Occup Environ Health. 2012;85(8):849-52.
105. Vepsäläinen H, Hautaniemi H, Sääksjärvi K, Leppänen MH, Nissinen K, Suhonen E, et al. Do stressed children have a lot on their plates? A cross-sectional study of long-term stress and diet among Finnish preschoolers. Appetite. 2021;157:104993.
106. Walton DM, Macdermid JC, Russell E, Koren G, Van Uum S. Hair-Normalized Cortisol Waking Response as a Novel Biomarker of Hypothalamic-Pituitary-Adrenal Axis Activity following Acute Trauma: A Proof-of-Concept Study with Pilot Results. Pain Res Treat. 2013;2013:876871.
107. Wester VL, Noppe G, Savas M, van den Akker ELT, de Rijke YB, van Rossum EFC. Hair analysis reveals subtle HPA axis suppression associated with use of local corticosteroids: The Lifelines cohort study. Psychoneuroendocrinology. 2017;80:1-6.
108. Wester VL, Reincke M, Koper JW, van den Akker ELT, Manenschijn L, Berr CM, et al. Scalp hair cortisol for diagnosis of Cushing's syndrome. Eur J Endocrinol. 2017;176(6):695-703.
109. Manenschijn L, Koper JW, Lamberts SWJ, Van Rossum EFC. Evaluation of a method to measure long term cortisol levels. Steroids. 2011;76(10):1032-6.
110. Stalder T, Kirschbaum C, Alexander N, Bornstein SR, Gao W, Miller R, et al. Cortisol in hair and the metabolic syndrome. J Clin Endocrinol Metab. 2013;98(6):2573-80.
111. Žekas V, Matuzevičiene R, Karčiauskaite D, Mažeikiene A, Burokiene N, Radzevičius M, et al. Chronic and oxidative stress association with total count of endothelial microvesicles in healthy young male plasma. Adv Clin Exp Med. 2019;28(5):683-92.
112. Lehto E, Ray C, Vepsäläinen H, Korkalo L, Lehto R, Kaukonen R, et al. Increased health and wellbeing in preschools (DAGIS) study—Differences in children's energy balance-related behaviors (EBRBs) and in long-term stress by parental educational level. Int J Environ Res Public Health. 2018;15(10):2313.
113. Lehrer HM, Goosby BJ, Dubois SK, Laudenslager ML, Steinhardt MA. Race moderates the association of perceived everyday discrimination and hair cortisol concentration. Stress. 2020;23(5):539-7.

114. Lanfear JH, Voegel CD, Binz TM, Paul RA. Hair cortisol measurement in older adults: Influence of demographic and physiological factors and correlation with perceived stress. Steroids. 2020;163:108712.
115. O'Brien KM, Tronick EZ, Moore CL. Relationship between hair cortisol and perceived chronic stress in a diverse sample. Stress Health. 2013;29(4):337-44.
116. White LO, Ising M, von Klitzing K, Sierau S, Michel A, Klein AM, et al. Reduced hair cortisol after maltreatment mediates externalizing symptoms in middle childhood and adolescence. J Child Psychol Psychiatry. 2017;58(9):998-1007.
117. Condon EM, Holland ML, Slade A, Redeker NS, Mayes LC, Sadler LS. Associations Between Maternal Caregiving and Child Indicators of Toxic Stress Among Multiethnic Urban Families. J Pediatr Health Care. 2019;33(4):425-36.
118. Föcker M, Stalder T, Kirschbaum C, Albrecht M, Adams F, de Zwaan M, et al. Hair Cortisol Concentrations in Adolescent Girls with Anorexia Nervosa are Lower Compared to Healthy and Psychiatric Controls. Eur Eating Disord Rev. 2016;24(6):531-5.
119. Smith L, Firth J, Grabovac I, Koyanagi A, Veronese N, Stubbs B, et al. The association of grip strength with depressive symptoms and cortisol in hair: A cross-sectional study of older adults. Scand J Med Sci Sports. 2019;29(10):1604-9.
120. Cedillo YE, Lomax RO, Fernandez JR, Moellering DR. Physiological Significance of Discrimination on Stress Markers, Obesity, and LDL Oxidation among a European American and African American Cohort of Females. Int J Behav Med. 2020;17(10):976-86.
