# Supplementary figures and images for: Cross‐sectional relation of long‐term glucocorticoids in hair with anthropometric measurements and their possible determinants: A systematic review and meta‐analysis
Source: Obes Rev. 2021 Nov 22;23(3):e13376. doi: 10.1111/obr.13376 (PMC9285618; doi:10.1111/obr.13376)

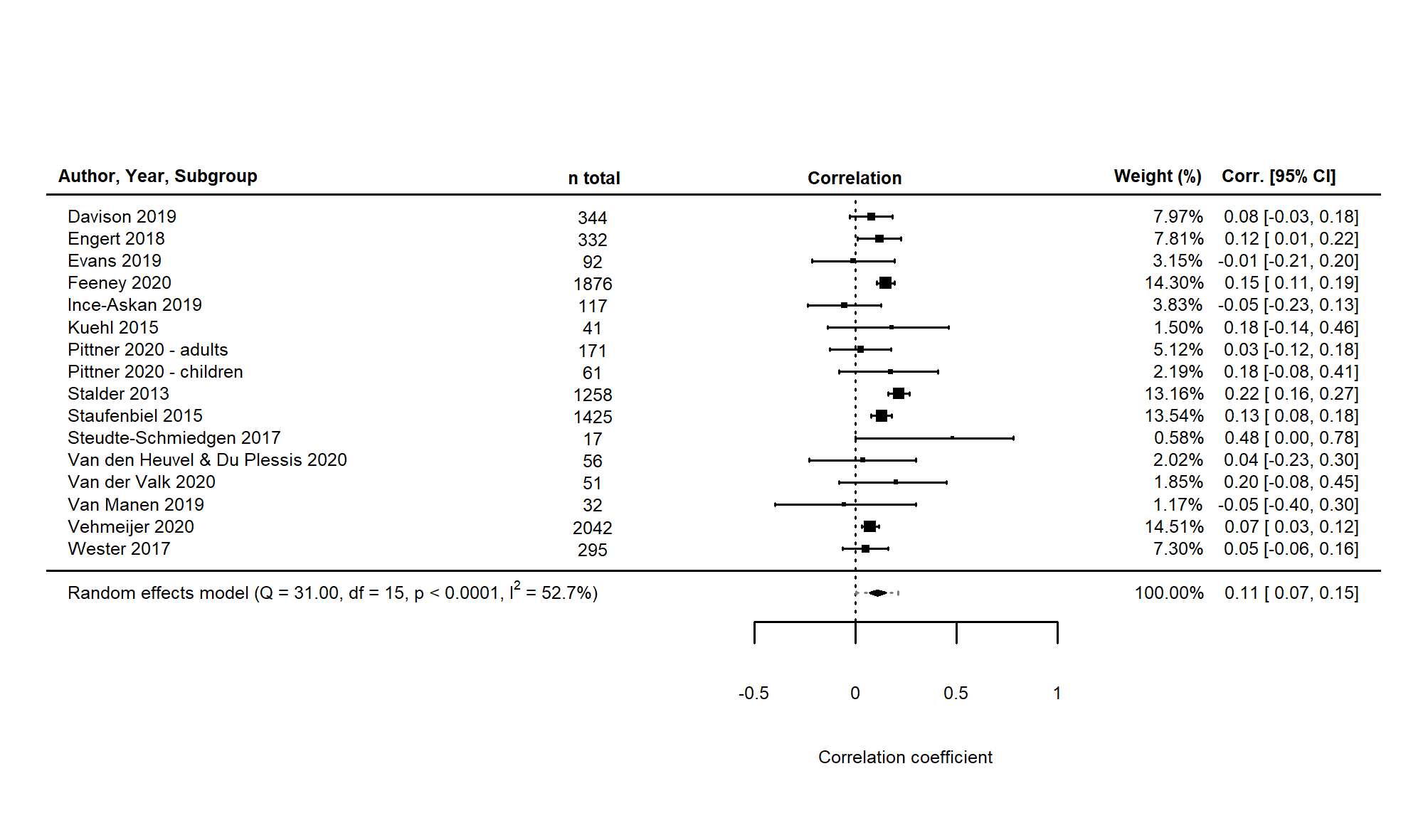

Supplement: Supplementary file 1 — Appendix S1. Search strategy. Table S1. Qualitative synthesis. Figure S1. Forest plot for the meta‐analysis of correlation coefficients between HairF and BMI. Figure S2. Forest plot for the meta‐analysis of correlation coefficients between HairF and BMI SDS. Figure S3. Forest plot for the meta‐analysis of correlation coefficients between HairF and WC. Figure S4. Forest plot for the meta‐analysis of correlation coefficients between HairF and WHR. Figure S5. Forest plot for the meta‐analysis of correlation coefficients between HairE and BMI. Figure S6. Forest plot for the meta‐analysis of correlation coefficients between HairE and WC. Figure S7. Bubble plot for the meta‐regression on proportion of males in the meta‐analysis of correlation coefficients between HairF and WC. Figure S8. Bubble plot for the meta‐regression on proportion of males in the meta‐analysis of correlations between HairF and WHR. Figure S9. Bubble plot for the meta‐regression on proportion of individuals with obesity in the meta‐analysis of correlations between HairF and BMI. Figure S10. Funnel plot for the meta‐analysis of correlation coefficients between HairF and BMI. Figure S11. Funnel plot for the meta‐analysis of correlation coefficients between HairF and BMI SDS. Figure S12. Funnel plot for the meta‐analysis of correlation coefficients between HairF and WC. Figure S13. Funnel plot for the meta‐analysis of correlation coefficients between HairF and WHR. Figure S14. Funnel plot for the meta‐analysis of correlation coefficients between HairE and BMI. Figure S15. Funnel plot for the meta‐analysis of correlation coefficients between HairE and WC. [file OBR-23-0-s001.zip › obr13376-sup-0002-Figure S5.tiff]

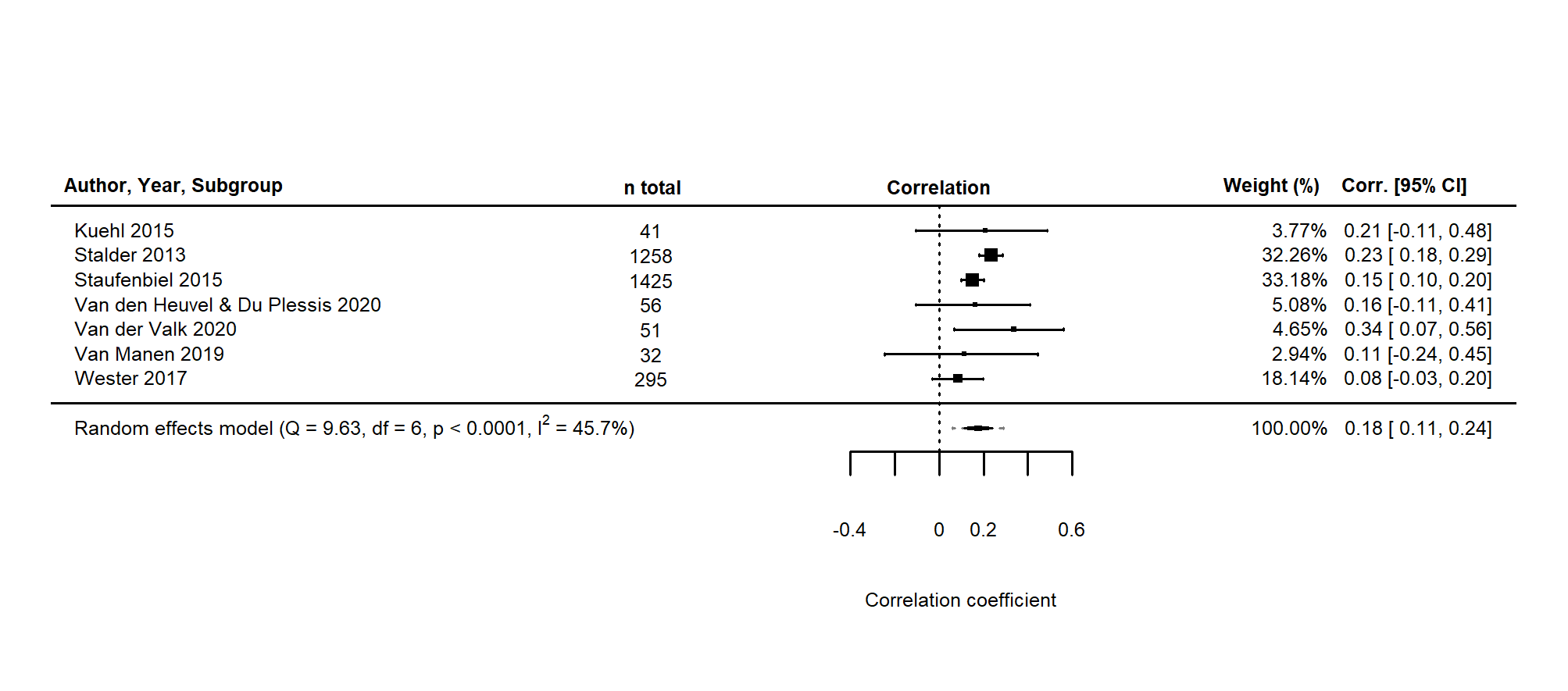

Supplement: Supplementary file 1 — Appendix S1. Search strategy. Table S1. Qualitative synthesis. Figure S1. Forest plot for the meta‐analysis of correlation coefficients between HairF and BMI. Figure S2. Forest plot for the meta‐analysis of correlation coefficients between HairF and BMI SDS. Figure S3. Forest plot for the meta‐analysis of correlation coefficients between HairF and WC. Figure S4. Forest plot for the meta‐analysis of correlation coefficients between HairF and WHR. Figure S5. Forest plot for the meta‐analysis of correlation coefficients between HairE and BMI. Figure S6. Forest plot for the meta‐analysis of correlation coefficients between HairE and WC. Figure S7. Bubble plot for the meta‐regression on proportion of males in the meta‐analysis of correlation coefficients between HairF and WC. Figure S8. Bubble plot for the meta‐regression on proportion of males in the meta‐analysis of correlations between HairF and WHR. Figure S9. Bubble plot for the meta‐regression on proportion of individuals with obesity in the meta‐analysis of correlations between HairF and BMI. Figure S10. Funnel plot for the meta‐analysis of correlation coefficients between HairF and BMI. Figure S11. Funnel plot for the meta‐analysis of correlation coefficients between HairF and BMI SDS. Figure S12. Funnel plot for the meta‐analysis of correlation coefficients between HairF and WC. Figure S13. Funnel plot for the meta‐analysis of correlation coefficients between HairF and WHR. Figure S14. Funnel plot for the meta‐analysis of correlation coefficients between HairE and BMI. Figure S15. Funnel plot for the meta‐analysis of correlation coefficients between HairE and WC. [file OBR-23-0-s001.zip › obr13376-sup-0003-Figure S6.tiff]

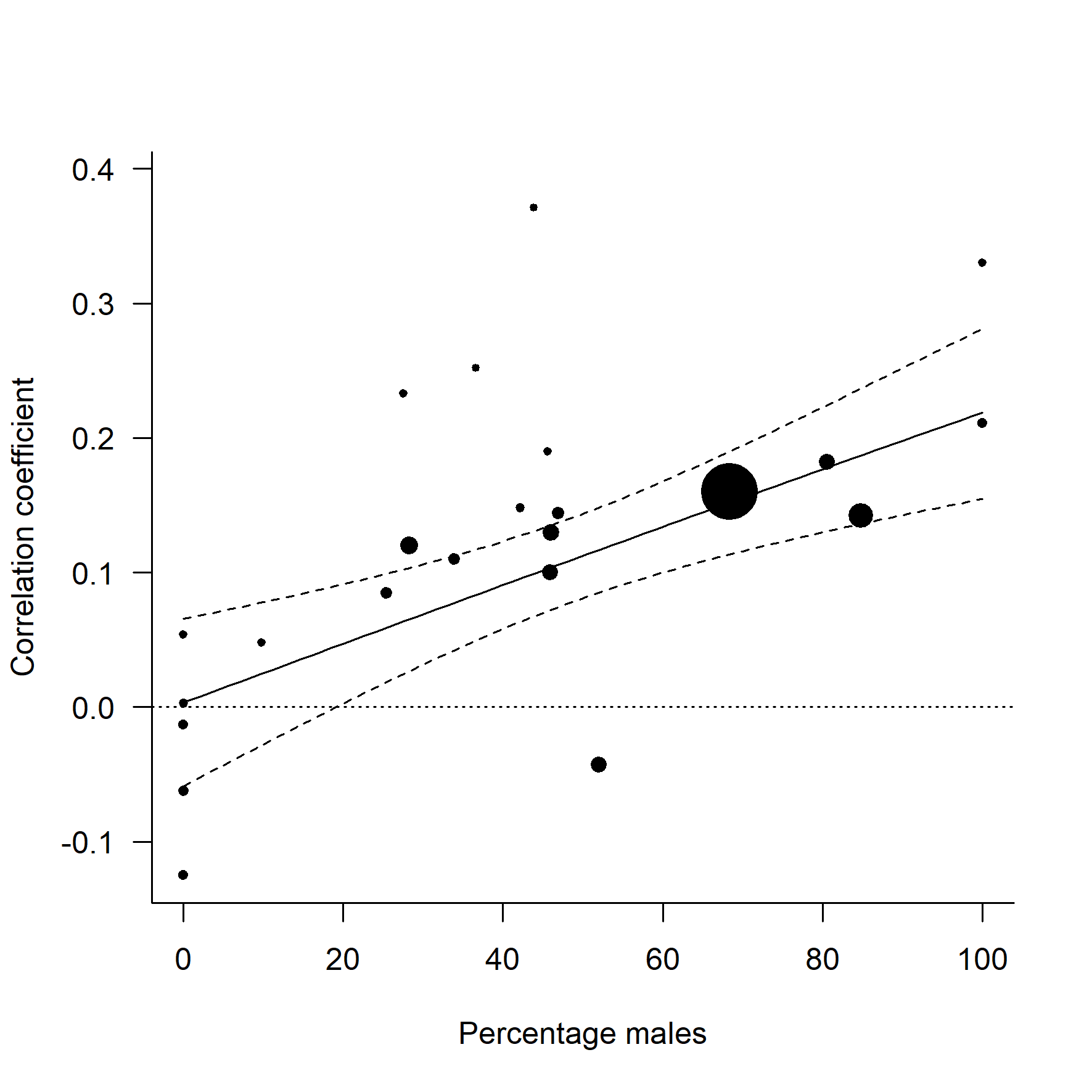

Supplement: Supplementary file 1 — Appendix S1. Search strategy. Table S1. Qualitative synthesis. Figure S1. Forest plot for the meta‐analysis of correlation coefficients between HairF and BMI. Figure S2. Forest plot for the meta‐analysis of correlation coefficients between HairF and BMI SDS. Figure S3. Forest plot for the meta‐analysis of correlation coefficients between HairF and WC. Figure S4. Forest plot for the meta‐analysis of correlation coefficients between HairF and WHR. Figure S5. Forest plot for the meta‐analysis of correlation coefficients between HairE and BMI. Figure S6. Forest plot for the meta‐analysis of correlation coefficients between HairE and WC. Figure S7. Bubble plot for the meta‐regression on proportion of males in the meta‐analysis of correlation coefficients between HairF and WC. Figure S8. Bubble plot for the meta‐regression on proportion of males in the meta‐analysis of correlations between HairF and WHR. Figure S9. Bubble plot for the meta‐regression on proportion of individuals with obesity in the meta‐analysis of correlations between HairF and BMI. Figure S10. Funnel plot for the meta‐analysis of correlation coefficients between HairF and BMI. Figure S11. Funnel plot for the meta‐analysis of correlation coefficients between HairF and BMI SDS. Figure S12. Funnel plot for the meta‐analysis of correlation coefficients between HairF and WC. Figure S13. Funnel plot for the meta‐analysis of correlation coefficients between HairF and WHR. Figure S14. Funnel plot for the meta‐analysis of correlation coefficients between HairE and BMI. Figure S15. Funnel plot for the meta‐analysis of correlation coefficients between HairE and WC. [file OBR-23-0-s001.zip › obr13376-sup-0004-Figure S7.png]

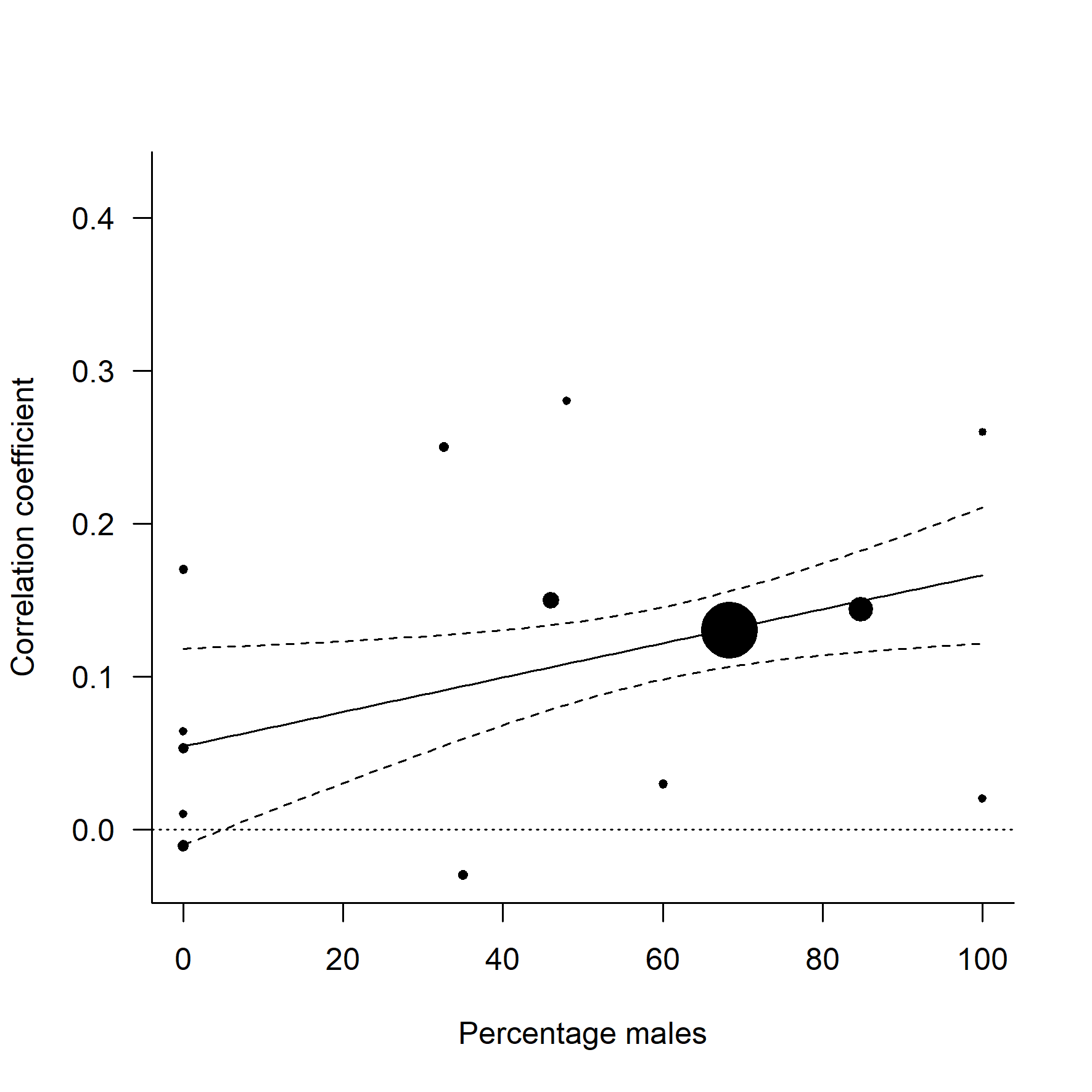

Supplement: Supplementary file 1 — Appendix S1. Search strategy. Table S1. Qualitative synthesis. Figure S1. Forest plot for the meta‐analysis of correlation coefficients between HairF and BMI. Figure S2. Forest plot for the meta‐analysis of correlation coefficients between HairF and BMI SDS. Figure S3. Forest plot for the meta‐analysis of correlation coefficients between HairF and WC. Figure S4. Forest plot for the meta‐analysis of correlation coefficients between HairF and WHR. Figure S5. Forest plot for the meta‐analysis of correlation coefficients between HairE and BMI. Figure S6. Forest plot for the meta‐analysis of correlation coefficients between HairE and WC. Figure S7. Bubble plot for the meta‐regression on proportion of males in the meta‐analysis of correlation coefficients between HairF and WC. Figure S8. Bubble plot for the meta‐regression on proportion of males in the meta‐analysis of correlations between HairF and WHR. Figure S9. Bubble plot for the meta‐regression on proportion of individuals with obesity in the meta‐analysis of correlations between HairF and BMI. Figure S10. Funnel plot for the meta‐analysis of correlation coefficients between HairF and BMI. Figure S11. Funnel plot for the meta‐analysis of correlation coefficients between HairF and BMI SDS. Figure S12. Funnel plot for the meta‐analysis of correlation coefficients between HairF and WC. Figure S13. Funnel plot for the meta‐analysis of correlation coefficients between HairF and WHR. Figure S14. Funnel plot for the meta‐analysis of correlation coefficients between HairE and BMI. Figure S15. Funnel plot for the meta‐analysis of correlation coefficients between HairE and WC. [file OBR-23-0-s001.zip › obr13376-sup-0005-Figure S8.png]

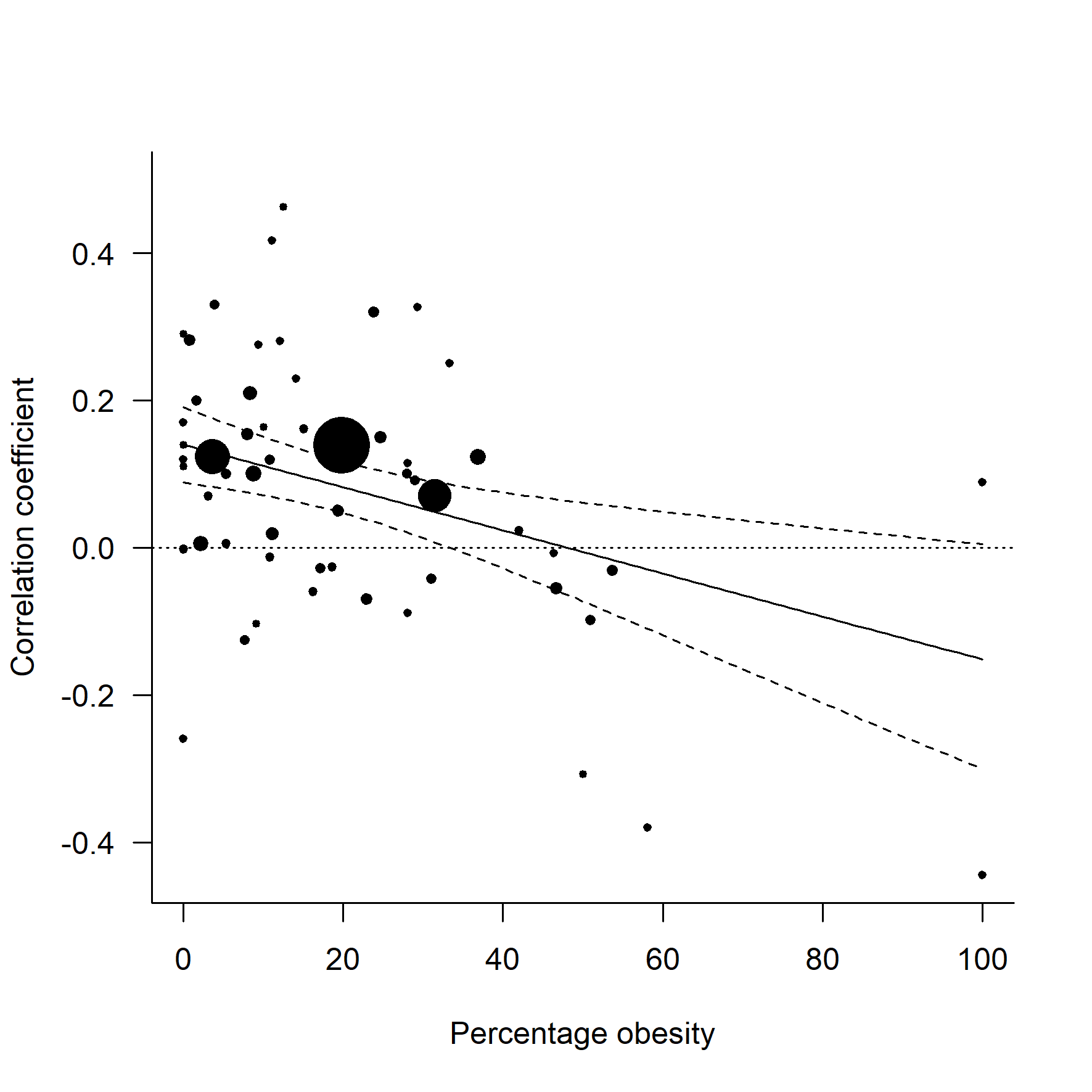

Supplement: Supplementary file 1 — Appendix S1. Search strategy. Table S1. Qualitative synthesis. Figure S1. Forest plot for the meta‐analysis of correlation coefficients between HairF and BMI. Figure S2. Forest plot for the meta‐analysis of correlation coefficients between HairF and BMI SDS. Figure S3. Forest plot for the meta‐analysis of correlation coefficients between HairF and WC. Figure S4. Forest plot for the meta‐analysis of correlation coefficients between HairF and WHR. Figure S5. Forest plot for the meta‐analysis of correlation coefficients between HairE and BMI. Figure S6. Forest plot for the meta‐analysis of correlation coefficients between HairE and WC. Figure S7. Bubble plot for the meta‐regression on proportion of males in the meta‐analysis of correlation coefficients between HairF and WC. Figure S8. Bubble plot for the meta‐regression on proportion of males in the meta‐analysis of correlations between HairF and WHR. Figure S9. Bubble plot for the meta‐regression on proportion of individuals with obesity in the meta‐analysis of correlations between HairF and BMI. Figure S10. Funnel plot for the meta‐analysis of correlation coefficients between HairF and BMI. Figure S11. Funnel plot for the meta‐analysis of correlation coefficients between HairF and BMI SDS. Figure S12. Funnel plot for the meta‐analysis of correlation coefficients between HairF and WC. Figure S13. Funnel plot for the meta‐analysis of correlation coefficients between HairF and WHR. Figure S14. Funnel plot for the meta‐analysis of correlation coefficients between HairE and BMI. Figure S15. Funnel plot for the meta‐analysis of correlation coefficients between HairE and WC. [file OBR-23-0-s001.zip › obr13376-sup-0006-Figure S9.png]

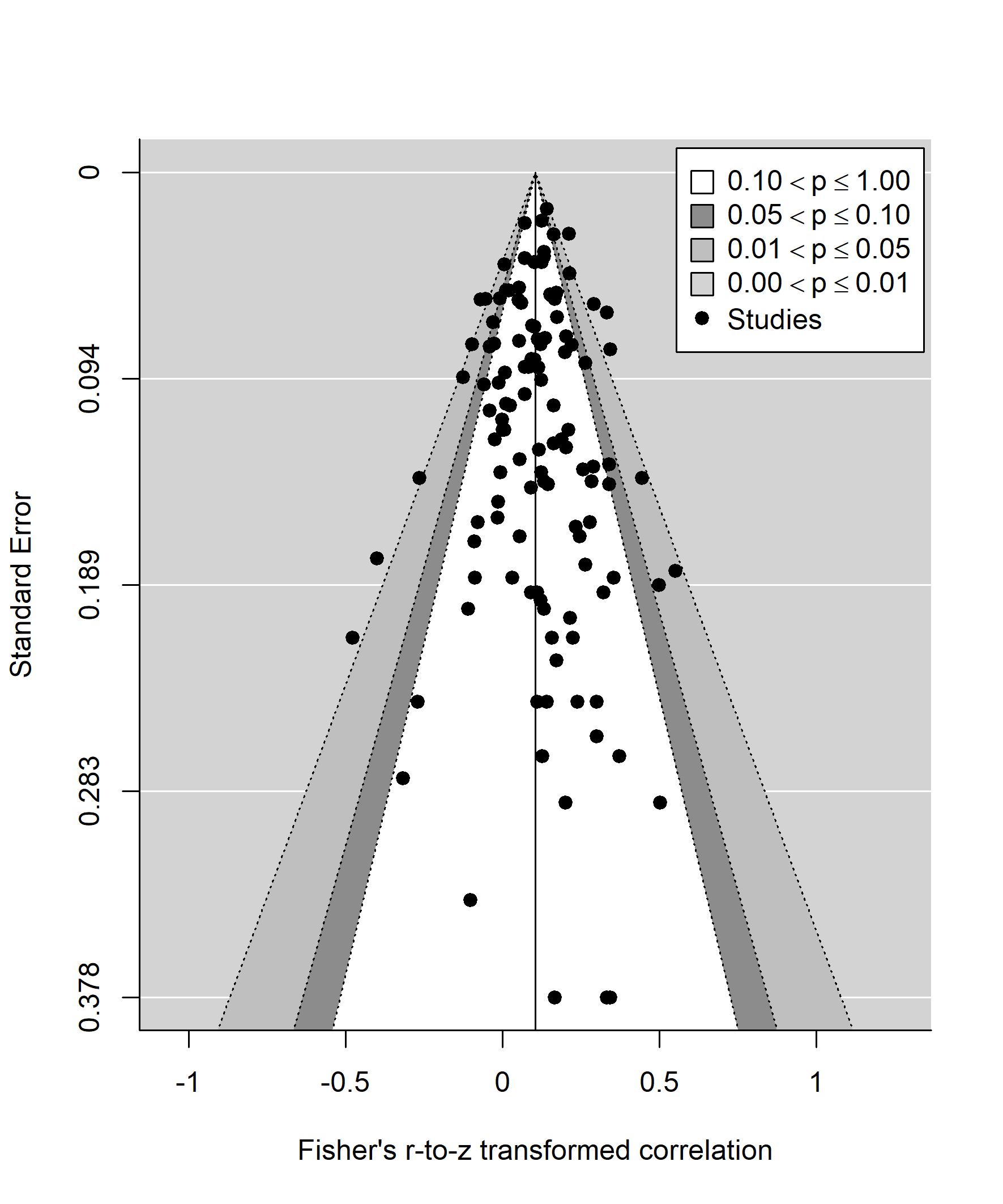

Supplement: Supplementary file 1 — Appendix S1. Search strategy. Table S1. Qualitative synthesis. Figure S1. Forest plot for the meta‐analysis of correlation coefficients between HairF and BMI. Figure S2. Forest plot for the meta‐analysis of correlation coefficients between HairF and BMI SDS. Figure S3. Forest plot for the meta‐analysis of correlation coefficients between HairF and WC. Figure S4. Forest plot for the meta‐analysis of correlation coefficients between HairF and WHR. Figure S5. Forest plot for the meta‐analysis of correlation coefficients between HairE and BMI. Figure S6. Forest plot for the meta‐analysis of correlation coefficients between HairE and WC. Figure S7. Bubble plot for the meta‐regression on proportion of males in the meta‐analysis of correlation coefficients between HairF and WC. Figure S8. Bubble plot for the meta‐regression on proportion of males in the meta‐analysis of correlations between HairF and WHR. Figure S9. Bubble plot for the meta‐regression on proportion of individuals with obesity in the meta‐analysis of correlations between HairF and BMI. Figure S10. Funnel plot for the meta‐analysis of correlation coefficients between HairF and BMI. Figure S11. Funnel plot for the meta‐analysis of correlation coefficients between HairF and BMI SDS. Figure S12. Funnel plot for the meta‐analysis of correlation coefficients between HairF and WC. Figure S13. Funnel plot for the meta‐analysis of correlation coefficients between HairF and WHR. Figure S14. Funnel plot for the meta‐analysis of correlation coefficients between HairE and BMI. Figure S15. Funnel plot for the meta‐analysis of correlation coefficients between HairE and WC. [file OBR-23-0-s001.zip › obr13376-sup-0007-Figure S10.tiff]

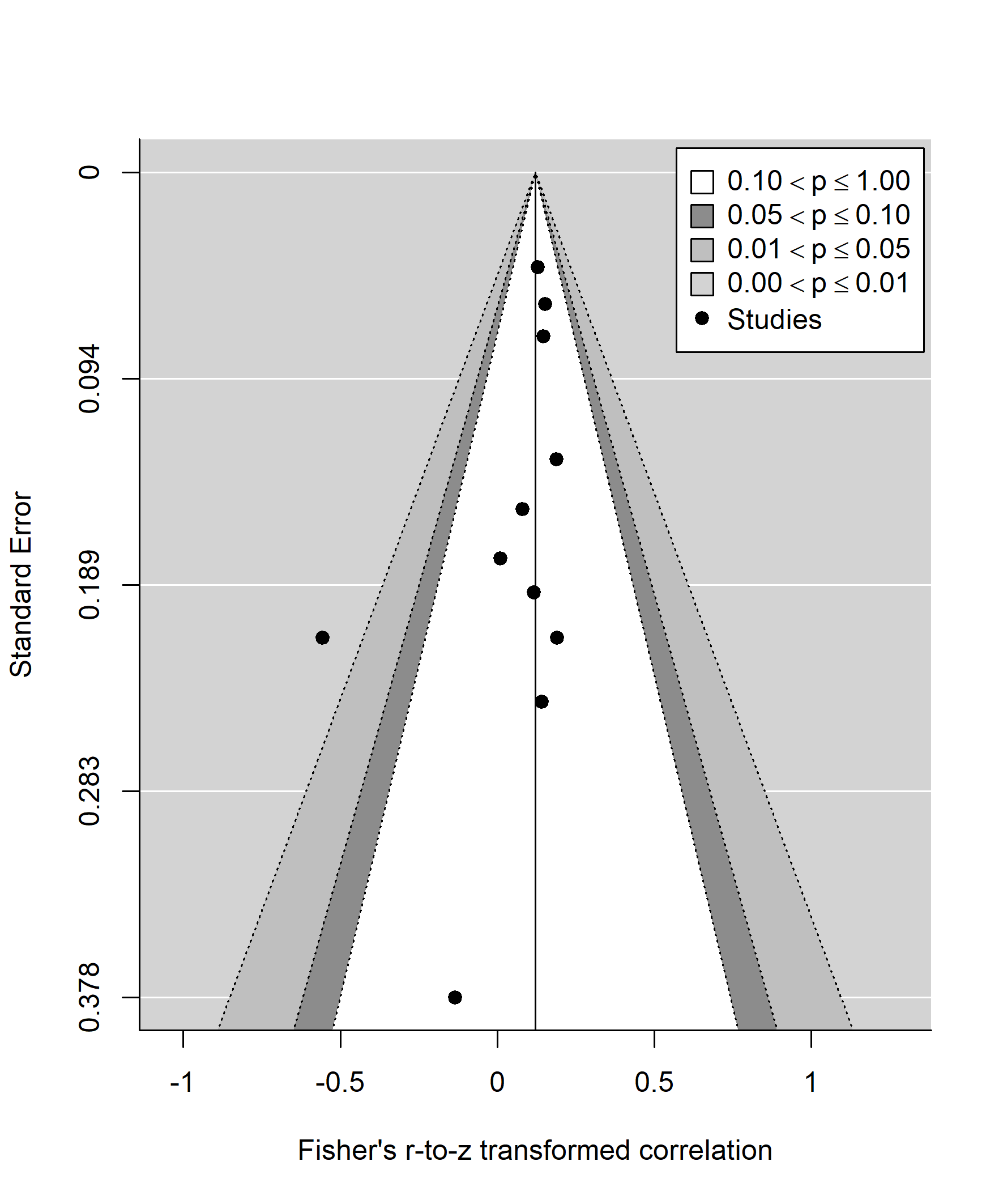

Supplement: Supplementary file 1 — Appendix S1. Search strategy. Table S1. Qualitative synthesis. Figure S1. Forest plot for the meta‐analysis of correlation coefficients between HairF and BMI. Figure S2. Forest plot for the meta‐analysis of correlation coefficients between HairF and BMI SDS. Figure S3. Forest plot for the meta‐analysis of correlation coefficients between HairF and WC. Figure S4. Forest plot for the meta‐analysis of correlation coefficients between HairF and WHR. Figure S5. Forest plot for the meta‐analysis of correlation coefficients between HairE and BMI. Figure S6. Forest plot for the meta‐analysis of correlation coefficients between HairE and WC. Figure S7. Bubble plot for the meta‐regression on proportion of males in the meta‐analysis of correlation coefficients between HairF and WC. Figure S8. Bubble plot for the meta‐regression on proportion of males in the meta‐analysis of correlations between HairF and WHR. Figure S9. Bubble plot for the meta‐regression on proportion of individuals with obesity in the meta‐analysis of correlations between HairF and BMI. Figure S10. Funnel plot for the meta‐analysis of correlation coefficients between HairF and BMI. Figure S11. Funnel plot for the meta‐analysis of correlation coefficients between HairF and BMI SDS. Figure S12. Funnel plot for the meta‐analysis of correlation coefficients between HairF and WC. Figure S13. Funnel plot for the meta‐analysis of correlation coefficients between HairF and WHR. Figure S14. Funnel plot for the meta‐analysis of correlation coefficients between HairE and BMI. Figure S15. Funnel plot for the meta‐analysis of correlation coefficients between HairE and WC. [file OBR-23-0-s001.zip › obr13376-sup-0008-Figure S11.tiff]

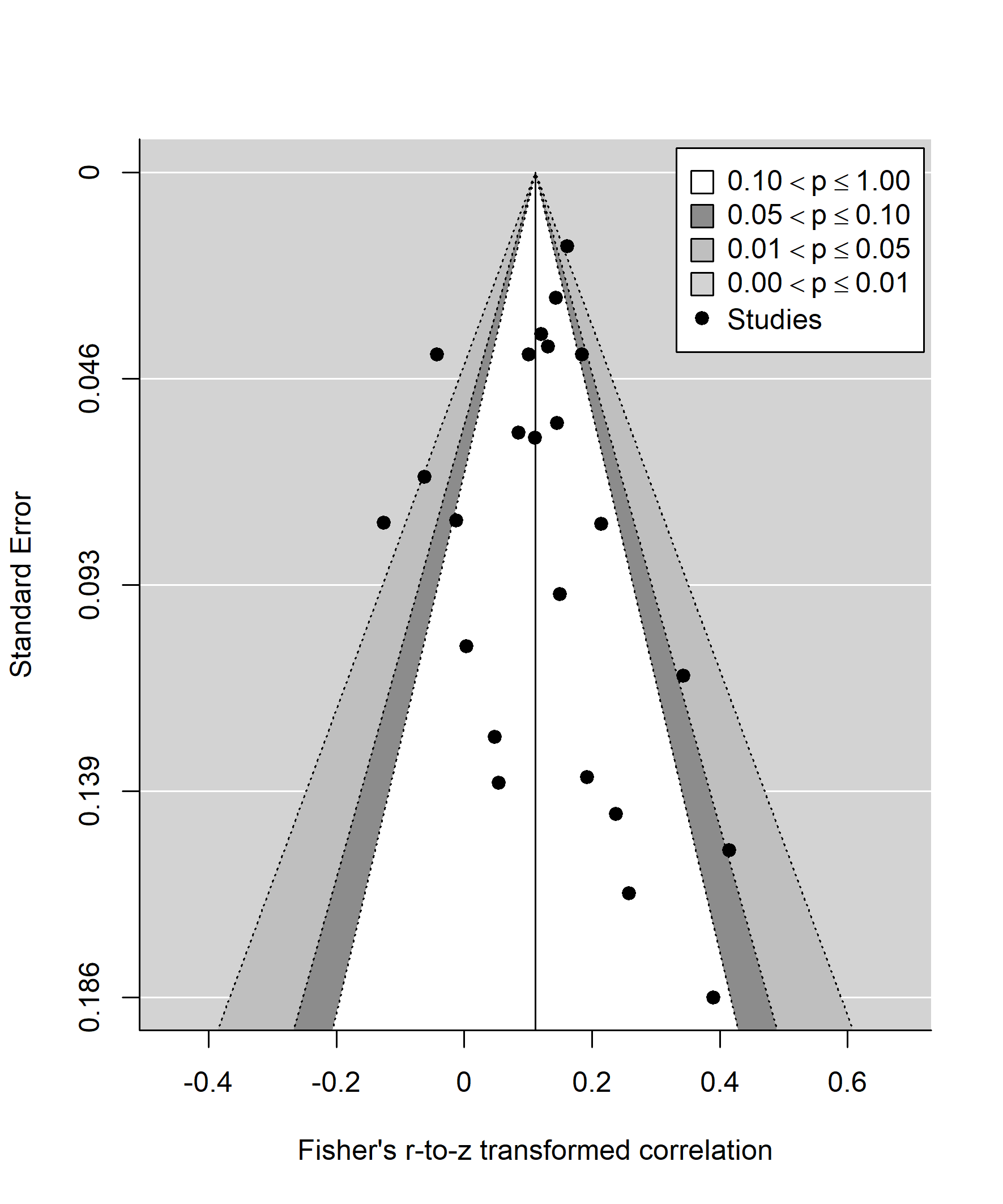

Supplement: Supplementary file 1 — Appendix S1. Search strategy. Table S1. Qualitative synthesis. Figure S1. Forest plot for the meta‐analysis of correlation coefficients between HairF and BMI. Figure S2. Forest plot for the meta‐analysis of correlation coefficients between HairF and BMI SDS. Figure S3. Forest plot for the meta‐analysis of correlation coefficients between HairF and WC. Figure S4. Forest plot for the meta‐analysis of correlation coefficients between HairF and WHR. Figure S5. Forest plot for the meta‐analysis of correlation coefficients between HairE and BMI. Figure S6. Forest plot for the meta‐analysis of correlation coefficients between HairE and WC. Figure S7. Bubble plot for the meta‐regression on proportion of males in the meta‐analysis of correlation coefficients between HairF and WC. Figure S8. Bubble plot for the meta‐regression on proportion of males in the meta‐analysis of correlations between HairF and WHR. Figure S9. Bubble plot for the meta‐regression on proportion of individuals with obesity in the meta‐analysis of correlations between HairF and BMI. Figure S10. Funnel plot for the meta‐analysis of correlation coefficients between HairF and BMI. Figure S11. Funnel plot for the meta‐analysis of correlation coefficients between HairF and BMI SDS. Figure S12. Funnel plot for the meta‐analysis of correlation coefficients between HairF and WC. Figure S13. Funnel plot for the meta‐analysis of correlation coefficients between HairF and WHR. Figure S14. Funnel plot for the meta‐analysis of correlation coefficients between HairE and BMI. Figure S15. Funnel plot for the meta‐analysis of correlation coefficients between HairE and WC. [file OBR-23-0-s001.zip › obr13376-sup-0009-Figure S12.tiff]

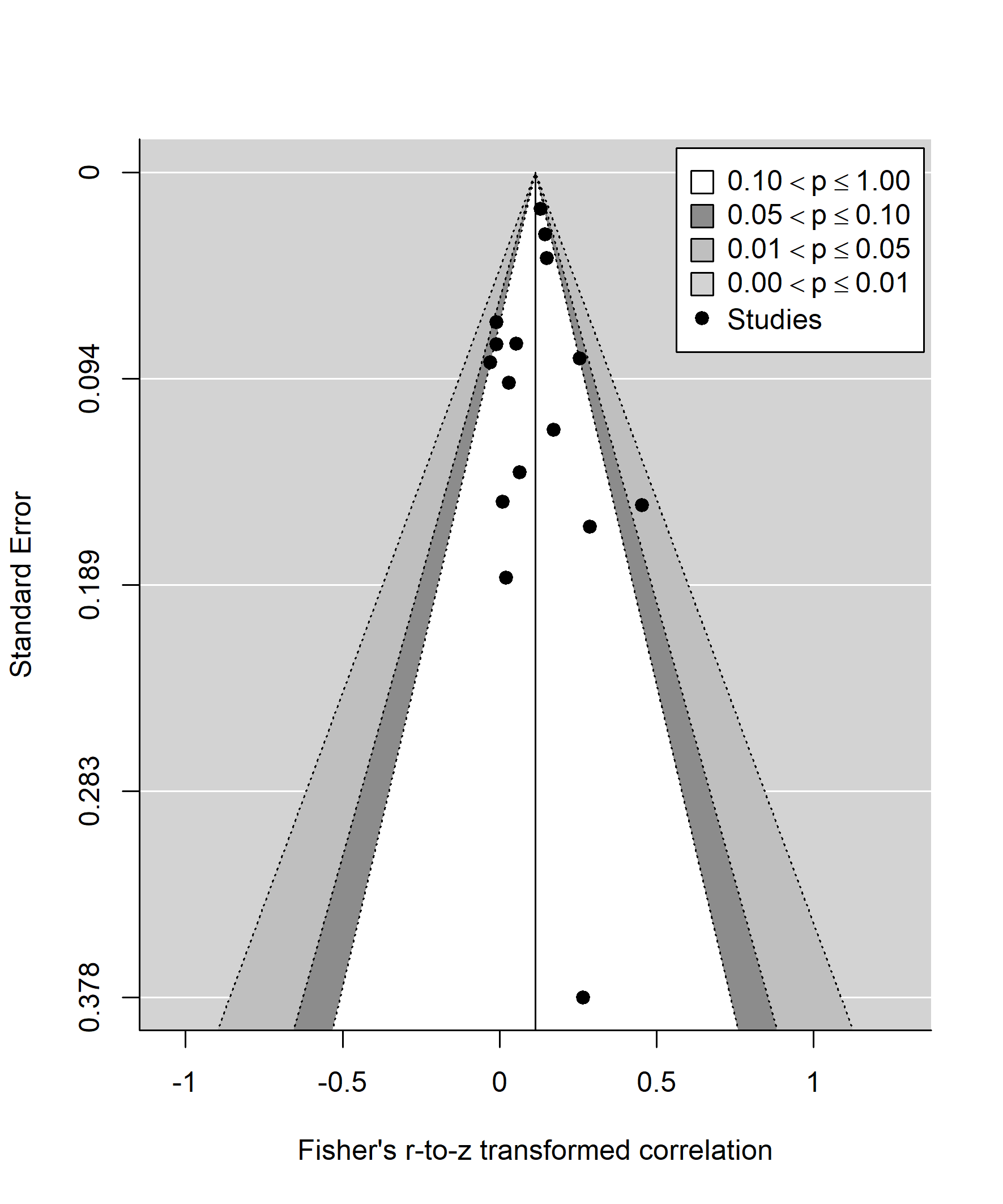

Supplement: Supplementary file 1 — Appendix S1. Search strategy. Table S1. Qualitative synthesis. Figure S1. Forest plot for the meta‐analysis of correlation coefficients between HairF and BMI. Figure S2. Forest plot for the meta‐analysis of correlation coefficients between HairF and BMI SDS. Figure S3. Forest plot for the meta‐analysis of correlation coefficients between HairF and WC. Figure S4. Forest plot for the meta‐analysis of correlation coefficients between HairF and WHR. Figure S5. Forest plot for the meta‐analysis of correlation coefficients between HairE and BMI. Figure S6. Forest plot for the meta‐analysis of correlation coefficients between HairE and WC. Figure S7. Bubble plot for the meta‐regression on proportion of males in the meta‐analysis of correlation coefficients between HairF and WC. Figure S8. Bubble plot for the meta‐regression on proportion of males in the meta‐analysis of correlations between HairF and WHR. Figure S9. Bubble plot for the meta‐regression on proportion of individuals with obesity in the meta‐analysis of correlations between HairF and BMI. Figure S10. Funnel plot for the meta‐analysis of correlation coefficients between HairF and BMI. Figure S11. Funnel plot for the meta‐analysis of correlation coefficients between HairF and BMI SDS. Figure S12. Funnel plot for the meta‐analysis of correlation coefficients between HairF and WC. Figure S13. Funnel plot for the meta‐analysis of correlation coefficients between HairF and WHR. Figure S14. Funnel plot for the meta‐analysis of correlation coefficients between HairE and BMI. Figure S15. Funnel plot for the meta‐analysis of correlation coefficients between HairE and WC. [file OBR-23-0-s001.zip › obr13376-sup-0010-Figure S13.tiff]

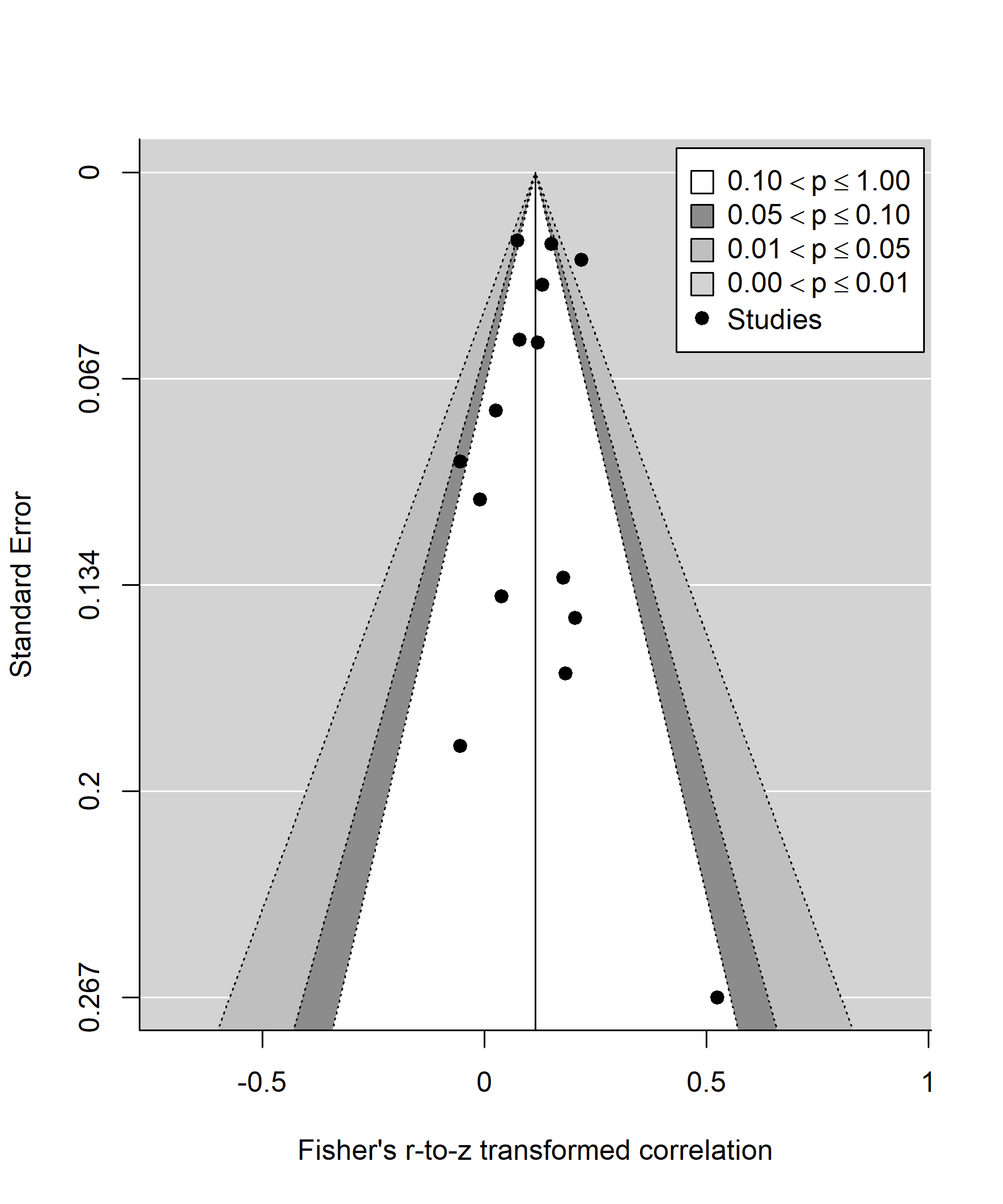

Supplement: Supplementary file 1 — Appendix S1. Search strategy. Table S1. Qualitative synthesis. Figure S1. Forest plot for the meta‐analysis of correlation coefficients between HairF and BMI. Figure S2. Forest plot for the meta‐analysis of correlation coefficients between HairF and BMI SDS. Figure S3. Forest plot for the meta‐analysis of correlation coefficients between HairF and WC. Figure S4. Forest plot for the meta‐analysis of correlation coefficients between HairF and WHR. Figure S5. Forest plot for the meta‐analysis of correlation coefficients between HairE and BMI. Figure S6. Forest plot for the meta‐analysis of correlation coefficients between HairE and WC. Figure S7. Bubble plot for the meta‐regression on proportion of males in the meta‐analysis of correlation coefficients between HairF and WC. Figure S8. Bubble plot for the meta‐regression on proportion of males in the meta‐analysis of correlations between HairF and WHR. Figure S9. Bubble plot for the meta‐regression on proportion of individuals with obesity in the meta‐analysis of correlations between HairF and BMI. Figure S10. Funnel plot for the meta‐analysis of correlation coefficients between HairF and BMI. Figure S11. Funnel plot for the meta‐analysis of correlation coefficients between HairF and BMI SDS. Figure S12. Funnel plot for the meta‐analysis of correlation coefficients between HairF and WC. Figure S13. Funnel plot for the meta‐analysis of correlation coefficients between HairF and WHR. Figure S14. Funnel plot for the meta‐analysis of correlation coefficients between HairE and BMI. Figure S15. Funnel plot for the meta‐analysis of correlation coefficients between HairE and WC. [file OBR-23-0-s001.zip › obr13376-sup-0011-Figure S14.tiff]

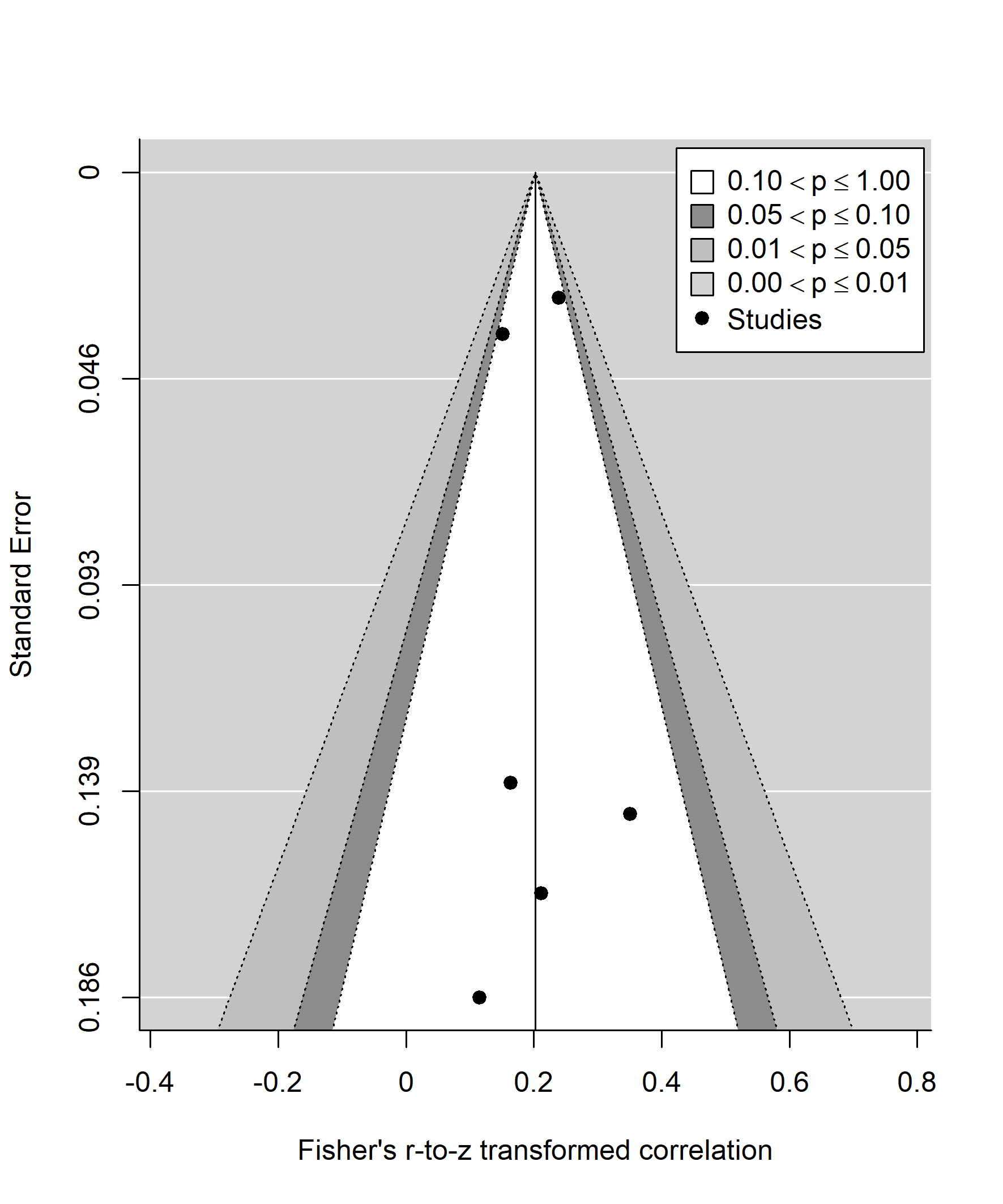

Supplement: Supplementary file 1 — Appendix S1. Search strategy. Table S1. Qualitative synthesis. Figure S1. Forest plot for the meta‐analysis of correlation coefficients between HairF and BMI. Figure S2. Forest plot for the meta‐analysis of correlation coefficients between HairF and BMI SDS. Figure S3. Forest plot for the meta‐analysis of correlation coefficients between HairF and WC. Figure S4. Forest plot for the meta‐analysis of correlation coefficients between HairF and WHR. Figure S5. Forest plot for the meta‐analysis of correlation coefficients between HairE and BMI. Figure S6. Forest plot for the meta‐analysis of correlation coefficients between HairE and WC. Figure S7. Bubble plot for the meta‐regression on proportion of males in the meta‐analysis of correlation coefficients between HairF and WC. Figure S8. Bubble plot for the meta‐regression on proportion of males in the meta‐analysis of correlations between HairF and WHR. Figure S9. Bubble plot for the meta‐regression on proportion of individuals with obesity in the meta‐analysis of correlations between HairF and BMI. Figure S10. Funnel plot for the meta‐analysis of correlation coefficients between HairF and BMI. Figure S11. Funnel plot for the meta‐analysis of correlation coefficients between HairF and BMI SDS. Figure S12. Funnel plot for the meta‐analysis of correlation coefficients between HairF and WC. Figure S13. Funnel plot for the meta‐analysis of correlation coefficients between HairF and WHR. Figure S14. Funnel plot for the meta‐analysis of correlation coefficients between HairE and BMI. Figure S15. Funnel plot for the meta‐analysis of correlation coefficients between HairE and WC. [file OBR-23-0-s001.zip › obr13376-sup-0012-Figure S15.tiff]
